# Unusual dimeric tetrahydroxanthone derivatives from *Aspergillus lentulus* and the determination of their axial chiralities

**DOI:** 10.1038/srep38958

**Published:** 2016-12-12

**Authors:** Tian-Xiao Li, Ming-Hua Yang, Ying Wang, Xiao-Bing Wang, Jun Luo, Jian-Guang Luo, Ling-Yi Kong

**Affiliations:** 1 State Key Laboratory of Natural Medicines, Department of Natural Medicinal Chemistry, China Pharmaceutical University, 24 Tong Jia Xiang, Nanjing 210009, People’s Republic of China

## Abstract

The research on secondary metabolites of *Aspergillus lentulus* afforded eight unusual heterodimeric tetrahydroxanthone derivatives, lentulins A−H (**2**−**9**), along with the known compound neosartorin (**1**). Compounds **1**−**6** exhibited potent antimicrobial activities especially against methicillin-resistant *Staphylococci*. Their absolute configurations, particularly the axial chiralities, were unambiguously demonstrated by a combination of electronic circular dichroism (ECD), Rh_2_(OCOCF_3_)_4_-induced ECD experiments, modified Mosher methods, and chemical conversions. Interestingly, compounds **1**–**4** were the first samples of atropisomers within the dimeric tetrahydroxanthone class. Further investigation of the relationships between their axial chiralities and ECD Cotton effects led to the proposal of a specific CD Exciton Chirality rule to determine the axial chiralities in dimeric tetrahydroxanthones and their derivatives.

Dimeric tetrahydroxanthones and their derivatives like xanthone−chromanone and chromanone dimers, with attractive antibacterial^1^,^2^, antifungal^3^ and antimalarial activities^4^, are a class of structurally interesting natural products produced by multiple fungal and lichenous species^5^. More than 70 members with 2,2′-, 4,4′- or 2,4′-axial linkages have been isolated and identified since 1958^5^. Biogenetically, most dimers are formed by two units from the same path^6^. Two paths derived heterodimers are rare and only four such molecules have been reported, namely eumitrins A1, A2, B, and neosartorin^7^,^8^.

All the dimeric tetrahydroxanthone analogues structurally involve axially chiral issues, which, different from the classic central chiralities, are interesting phenomena associated with the hindered rotation of a single bond^9^. Generally, a rotary energy barrier greater than 23.3 kcal/mol will prevent atropisomeric racemization and lead to stable axially chiral compounds^10^,^11^. Due to the presence of axial chiralities and their complicated spatial structures, the absolute configuration determinations of these compounds were quite challenging and mostly unsettled^1^,^4^,^12^,^13^. Electronic circular dichroism (ECD) calculation was the commonly used method. However, it was time-consuming to find the suitable conformers as well as choose the right calculation method. Sometimes, improper method could even lead to incorrect results^14^,^15^.

Recently, eight unusual heterodimeric tetrahydroxanthone derivatives, lentulins A−H (**2**−**9**), and the known neosartorin (**1**), were obtained during our metabolite research of the endophytic fungus *Aspergillus lentulus* (Fig. 1). Compounds **1**–**4** were the first samples of dimeric tetrahydroxanthone atropisomers, which along with other derivatives, gave useful samples to reveal the domination of axial chiralities in ECD Cotton effects (CEs). Based on the well determined absolute configurations and theoretical studies, the CD Exciton Chirality method was introduced to their axially chiral demonstration. In addition, these compounds exhibited potent antimicrobial activities especially against methicillin-resistant *Staphylococci*. Herein, we describe the structural elucidations, axially chiral researches and bioactive studies of compounds **1**−**9**.

## Result and Discussion

### Structural elucidation

**Figure 1:**
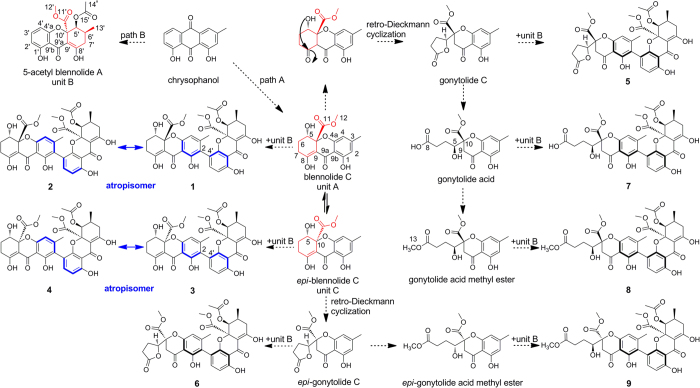
Structures and biogenesis relationships of 1–9.

The crude extract of *Aspergillus lentulus*, obtained from its rice cultures, was purified through silica gel and ODS column chromatography (CC), as well as preparative HPLC to yield compounds **1**–**9** (Fig. 1).

**Figure 2:**
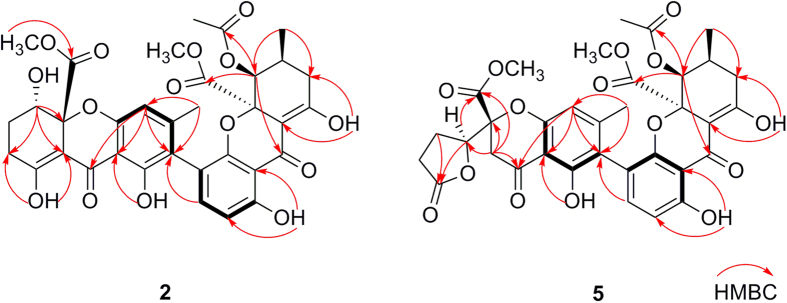
Selected HMBC correlations of 2 and 5.

Lentulin A (**2**) was obtained as a yellow powder. Its molecular formula, C_34_H_32_O_15_, was established from the positive HRESIMS ion at *m*/*z* 703.1631 (calculated for 703.1633, C_34_H_32_O_15_Na). UV absorption at 337 nm suggested the presence of a long conjugated moiety and IR absorptions at 3451 and 1740 cm^−1^ indicated the existence of hydroxy and carbonyl groups. The typically doubled NMR data (Supporting Information Tables S1 and S2), especially the presence of two conjugated ketone carbonyls (*δ*_C_ 187.8, 187.2) and two enolic groups (*δ*_H_ 14.11, 1 H, s and *δ*_C_ 178.4, 101.1; *δ*_H_ 14.02, 1 H, s and *δ*_C_ 179.3, 100.2), revealed the dimeric tetrahydroxanthone skeleton of **2**. Further comparison of its 1D NMR data and HMBC correlations (Fig. 2) with those of neosartorin (**1**)^16^ denoted the identical planar structure between them (Fig. 1), possessing 2,4′-linked blennolide C (unit A)^17^ and 5-acetyl blennolide A (unit B)^18^.

**Figure 3:**
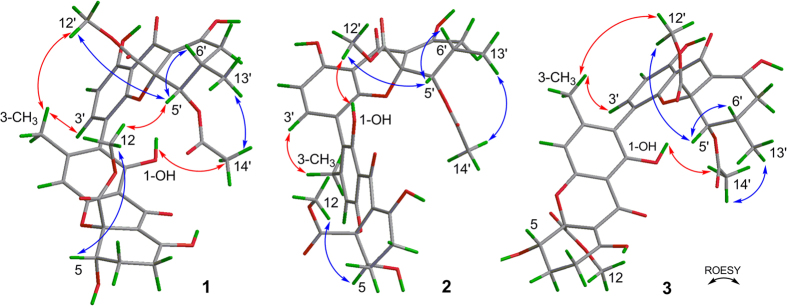
Key ROESY correlations of 1–3.

The relative configurations of units A and B in **2** were confirmed the same as those in **1** (Fig. 3), due to the similar NOEs of H-5/OCH_3_-12, H-5′/OCH_3_-12′, H-5′/H-6′, OCH_3_-12′/H-6′, and CH_3_-14′/CH_3_-13′^16^. However, the contrary optical rotations (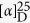 –210.2 for **1**; 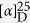 +276.1 for **2**) suggested the distinction of their axial chiralities^19^,^20^. More importantly, the different long range NOE correlations between units A and B (Fig. 3), especially the observed signal of OH-1/OCH_3_-12′ and the missing signals of OH-1/CH_3_-14′ and OCH_3_-12/H-5′, identified **2** as the atropisomer of **1** with aS* configuration, rather than the aR* in **1**^16^.

Lentulin B (**3**), a yellow powder, had the same molecular formula C_34_H_32_O_15_ as neosartorin (**1**). A comprehensive analysis of their NMR data (Supporting Information Tables S1 and S2) indicated that **3** was an epimer of **1**. Unlike neosartorin, the large coupling constants between H-5 and H-6 (^3^*J*_5–6ax_ = 12.4 Hz,^3^*J*_5–6eq_ = 4.8 Hz)^18^ suggested H-5 was in the axial position (Fig. 3). The lacked NOE signal between OCH_3_-12 and H-5 revealed their *trans* configuration and the absent ROESY correlation of OCH_3_-12/H-5′ denoted the *α*-orientation of COOCH_3_-10. In addition, the similar ROESY cross-peaks of OH-1/CH_3_-14′, CH_3_-3/H-3′, and CH_3_-3/OCH_3_-12′ (Fig. 3), established the same aR* configuration as neosartorin.

Lentulin C (**4**) had the same molecular formula C_34_H_32_O_15_ (HRESIMS ion at *m*/*z* 703.1637) as **3**, and was deduced to be its isomer from their similar 1D NMR data (Supporting Information Tables S1 and S2). The aS* configuration was also demonstrated by the NOE correlations of OH-1/OCH_3_-12′ and CH_3_-3/H-3′.

Lentulins D (**5**) and E (**6**) were another pair of neosartorin derivatives. NMR data analysis verified their structural similarities with above compounds. However, the lack of enolic signals indicated the absence of olefinic bond (Δ^8(9)^). And the presence of ester carbonyls (*δ*_C_ 175.6 in **5**, *δ*_C_ 175.8 in **6**) and the additional methylenes (*δ*_H_ 3.21, 2.97, each 1 H, and *δ*_C_ 39.4 in **5**; *δ*_H_ 3.55, 3.07, each 1 H, and *δ*_C_ 40.3 in **6**) revealed the *γ*-lactone moieties^18^, that were further confirmed by the HMBC correlations from H-5 to C-8, and from H-9 to C-5, C-9a, C-10, and C-11. As derived from **1** and **3** respectively, they were epimers with differently oriented COOCH_3_-10, which were proved by the present NOE cross-peak of OCH_3_-12/H-5′ in **5** but absent in **6**. Similar to **1**, the aR* configurations of **5** and **6** were confirmed by the long ranged NOE signals of OH-1/CH_3_-14′, CH_3_-3/H-3′, and CH_3_-3/OCH_3_-12′.

HRESIMS (*m/z* 721.1736 [M + Na]^+^) gave the molecular formula C_34_H_34_O_16_ for lentulin F (**7**), with one more H_2_O unit than **5**. An intensive comparison of its NMR data (Supporting Information Tables S1 and S2) with those of **5** confirmed the *γ*-hydroxy butyric acid moiety in **7**^17^, notably through one degree loss of unsaturation and the lack of HMBC correlation from H-5 to C-8. The relative configuration of **7** was confirmed same as **5** from their identical NOE correlations.

**8** and **9** were the derivatives of **5** and **6** with the cleavages of *γ*-lactones and the formations of *γ*-hydroxy butyric acid methyl esters, which were denoted by the decreases of unsaturation and HMBC correlations from the additional methoxyls to C-8. Similar to **5** and **6**, the relative configurations of COOCH_3_-10 in **8** and **9** were confirmed as *β*- and *α*-orientation respectively. Their axial configurations were also assigned same as those of **5** and **6**.

**Figure 4:**
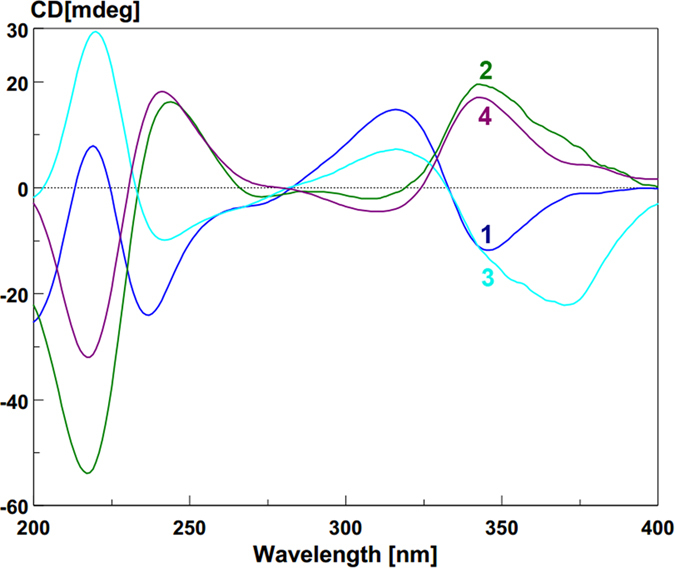
ECD spectra of 1–4 (in MeOH).

The absolute configuration (a*R*, 5*S*, 10*R*, 5′*S*, 6′*S*, 10′*R*) of neosartorin was determined by ECD calculation, which also predicted the approximate mirror image CEs of its atropisomer^16^. In the ECD spectrum of **2** (Fig. 4), the CEs well matched the calculation for neosartorin atropisomer with a*S*, 5*S*, 10*R*, 5′*S*, 6′*S*, 10′*R* configuration, proving that their CEs were governed by axial chiralities. The similar mirror images CEs were also found for **3** and **4** (Fig. 4). The identical CEs between **3** and **1** indicated the a*R* configuration in **3**, while **4** was assigned as the atropisomer of **3**. A Rh_2_(OCOCF_3_)_4_-induced ECD experiment (Supporting Information Figure S1) suggested the 5*S* configuration in **4** according to the Bulkiness rule^21^, confirming its absolute configuration as a*S*, 5*S*, 10*S*, 5′*S*, 6′*S*, 10′*R*. Interestingly, although **3** was the epimer of **1** with different central chiralities at C-10, the signs of their CEs were still the same, also suggesting the domination of axial chiralities in ECD spectra. So were compounds **4** and **2**.

**Figure 5:**
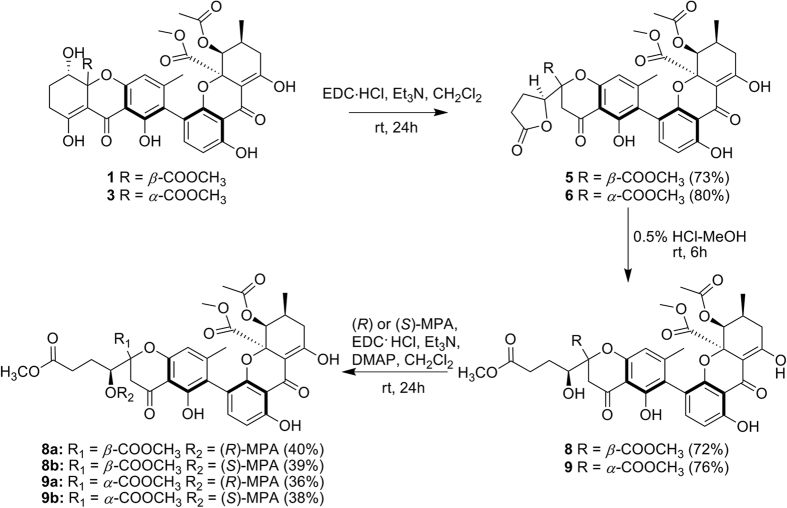
Chemical conversions of 1 and 3.

**Figure 6:**
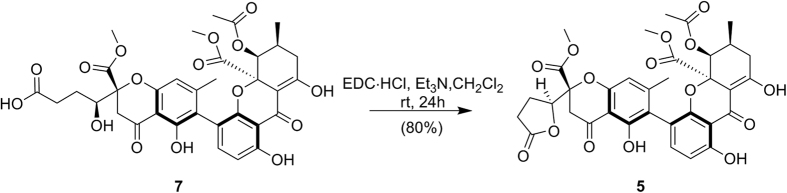
Chemical Conversion of 7 into 5.

**Figure 7:**
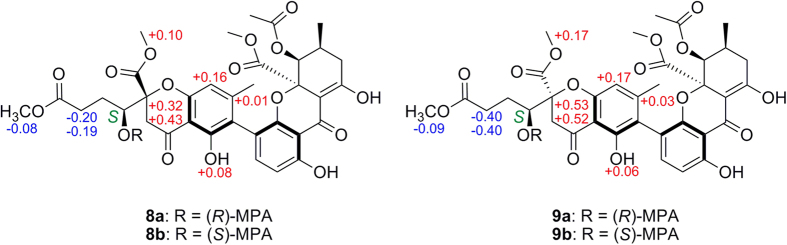
Δ*δ*_H(*R*−*S*)_ values of MPA esters for 8 and 9.

To avoid any ambiguity and demonstrate the absolute configurations of other derivatives, series of chemical conversions from **1** into **8**, **3** into **9**, and **7** into **5** were performed (Figs 5 and 6)^17^,^22^. Succeeding derivations of **8** and **9** by (*R*)- and (*S*)-methoxyphenylacetic acid (MPA) allowed the determination of their absolute configurations^22^. The negative Δ*δ*_H(*R*-*S*)_ values of OCH_3_-13 and H-7 as well as the positive Δ*δ*_H(*R*-*S*)_ values of OCH_3_-12 and H-9 (Fig. 7) confirmed 5*S* absolute configurations in both **8** and **9**. In combination with above chemical conversions, the 5*S* configurations in the other compounds were also revealed.

A plausible biosynthetic pathway was shown in Fig. 1. The oxidization and reduction of chrysophanol from the two paths gave units A and B^6^,^23^. Unit C (*epi*-blennolide C) was afforded via ring open and closure of unit A and both of them could convert into *γ*-lactone products through the retro-Dieckmann cyclization, which further yielded other ring cleavage intermediates^5^,^6^,^17^. Interestingly, all the biosynthetic modifications were occured on monomeric unit A, while the derivatization of unit B was limited to 5-acetylation that could prevent the further retro-Dieckmann cyclization. Meanwhile, the dimers were regularly formed by unit B with diverse path A units. These patterned dimerisations together with the specific monomeric modifications, indicated the enzymatic nature of these reactions, rather than the radical coupling^24^,^25^.

### CD Exciton Chirality method

**Figure 8:**
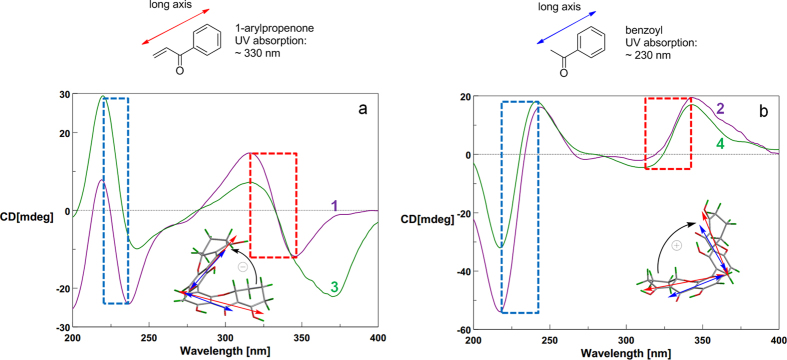
CD Exciton Chiralities of 1–4.

Tetrahydroxanthone monomers possessed two distinct kinds of chromophores (Fig. 8), namely 1-arylpropenone (330 nm) and benzoyl (230 nm). Their corresponding ECD CEs at above wavelengths were not split^18^. But for the axially linked dimers, their obviously split CEs suggested the two chromophores interacted with each other, and the opposite but not mirror image ECD spectrums (Fig. 4) revealed axial chiralities governed chromophore spatial positions to affect ECD CEs^26^,^27^.

**Figure 9:**
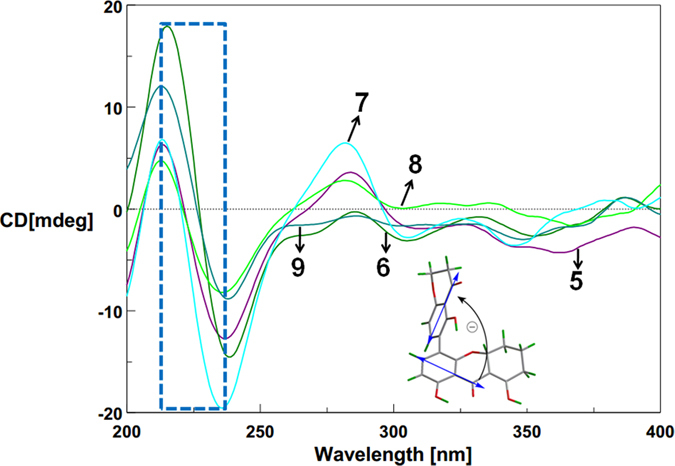
CD Exciton Chiralities of 5–9.

When watching parallel to the chiral axes, the two chromophores’ rotary manners were identical to those of the CD Exciton Chirality rule. In particular, the anticlockwise manner of two 1-arylpropenone chromophores^16^ led to negative exciton couplets centered at around 330 nm like **1** and **3** (Fig. 8a), otherwise positive like **2** and **4** (Fig. 8b). This deduction was simultaneously proved by the split CEs at 230 nm caused by two benzoyl chromophores in the same rotary manners. Interestingly, due to the cleavages of one 1-arylpropenone chromophore in **5**–**9**, their exciton couplets at 330 nm disappeared (Fig. 9), also suggesting the domination of chromophore interactions in ECD CEs. For further verification, different dimeric tetrahydroxanthone analogues were checked, which accurately revealed the same result as determined by X-ray analysis, chemical conversions or ECD calculations (Supporting Information Table S3)^15^,^17^,^28^,^29^.

The CD Exciton Chirality method has been applied to some axially chiral determinations before, such as those of binaphthyl, biarylic dihydronaphthopyranone^30^,^31^, and bis(naphtho-*γ*-pyrone)^32^. However, a very few exceptions were reported due to their complicated chromophores and non-negligible intense magnetic transition dipole moments^33^,^34^.

Theoretically, the rotational strengths ***R***^α^ and ***R***^β^ of *α* and *β* excited states of one exciton couplet can be defined as Equations (1) and (2)^33^,^35^ (**μ**, electric transition dipole moments; **m**, magnetic transition dipole moments):


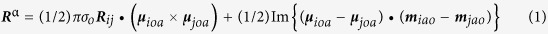



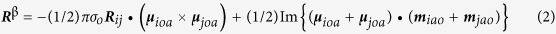


The first terms (**μμ** terms) were the rotational strengths caused by electric transition dipole moments **μ** and the second terms (**μm** terms) described the rotational strengths obtained by the combined electric and origin-independent magnetic transition moments. For the common π−π* excitation chromophores such as *p*-substituted benzoates, naphthoates, and anthroates, their internal magnetic transition moments (***m***_*iao*_, ***m***_*jao*_) were small and negligible, so were the **μm** terms^35^,^36^. The remaining terms (**μμ** terms) were the theoretical quantitative parameters of the CD Exciton Chirality rule^26^. Thus, based on the undisturbed benzoyl and 1-arylpropenone chromophores, the CD Exciton Chirality method could be used for the axial chiralities of dimeric tetrahydroxanthones and their derivatives, such as xanthone−chromanone and chromanone dimers.

### Antimicrobial Assays

Using broth microdilution method^37^, compounds **1**–**9** were evaluated for their antimicrobial activities against a panel of pathogenic microbes, including multidrug resistant clinical strains. As collated in Table 1, compounds **1**–**6** showed moderate to significant antibacterial activities against four strains of Gram-positive and three strains of Gram-negative bacteria. **3** was the most potent antibiotic among these compounds, especially against methicillin-resistant *Staphylococcus aureus* (MRSA) and methicillin-resistant *S. epidermidis* (MRSE). In view of the weakly active **5** and **6**, as well as the inactive ring-opening derivatives (**7**–**9**), 8-OH might be important for their antibacterial activities.

### Conclusion

In summary, our study on fungal metabolites afforded nine rare 2,4′-linked heterodimeric tetrahydroxanthone derivatives with attractive antibacterial activities. Compounds **1**–**4** were first isolated as two pairs of atropisomers, which along with other derivatives, provided great samples to reveal the dominant role of axial chiralities in CEs. The specific CD Exciton Chirality method was therefore proposed to determine the axial chiralities in dimeric tetrahydroxanthones and their derivatives.

## Methods

### General experimental procedures

Optical rotations were measured on a Jasco P-1020 polarimeter (Jasco, Tokyo, Japan). A UV-2450 spectrophotometer (Shimadzu, Tokyo, Japan) was applied for the measurement of UV spectra. ECD and IR data were collected on Jasco J-810 spectrometer and Bruker Tensor 27 spectrometer (Bruker, Karlsruhe, Germany), respectively. Preparative HPLC was performed on a Shimadzu LC-8A system equipped with Shim-pack RP-C_18_ column (10 *μ*m, 200 mm × 20 mm i.d., Shimadzu, Tokyo, Japan), using a binary channel UV detector at the wavelengths of 210 and 330 nm, respectively. With TMS as internal standard, NMR data were recorded on a Bruker AVIII-500 NMR instrument (^1^H NMR, 500 MHz; ^13^C NMR, 125 MHz). HRESIMS data were obtained using Agilent 6520B Q-TOF mass instrument and Agilent 1100 series LC/MSD-Trap-SL mass analyzer (Agilent Technologies, Santa Clara, USA). The extracts were fractionated through CCs with multiple packing materials such as silica gel (Qingdao Marine Chemical Co. Ltd., Qingdao, China), Sephadex LH-20 (Pharmacia, Stockholm, Sweden) and ODS (40−63 *μ*m, Fuji, Tokyo, Japan). After fluorescence observation under UV light (254 and 356 nm), silica gel GF_254_ plates were sprayed with vanillin−sulfuric acid to visualize the spots. Optical density (OD) values were determined by a microplate reader (Tecan, Mannedorf, Switzerland).

### Fungal material

The tubers of *Pinellia ternata* (Araceae) were collected from the suburb of Nanjing, Jiangsu, People’s Republic of China in May, 2014. After surface sterilization with 75% ethanol and 1% NaClO, the tubers were cut into small pieces, which were put on the potato dextrose agar to afford the title strain. From morphological and microscopic characteristics, this fungus was identified as *Aspergillus lentulus*, which was further reinforced by its internal transcribed spacer (ITS) and 18*S* rDNA sequences with 100% identity to the reported one (GenBank accession No. HE578064.1). The fungus was cultivated on potato dextrose agar (PDA) at 28 °C for 7 days. Then 16 pieces of the agar were transformed into four 250 mL Erlenmeyer flasks (containing 80 mL potato dextrose liquid medium), which were incubated at 28 °C, and 120 rpm for 6 days to prepare seed culture. A total of 15 Erlenmeyer flasks (500 mL), each containing 80 g of rice and 120 mL of tap water, were used for solid fermentation. After being sterilized at 115 °C for 30 minutes, the flasks were incubated with 20 mL of seed cultures to cultivate at 28 °C for 30 days.

### Extraction and isolation

The crude extract (15.2 g), obtained by extracting the rice cultures with EtOAc three times and removing EtOAc under reduced pressure, had obvious antibacterial activity with an MIC value of 162 *μ*g/mL for *S. aureus* ATCC 25923. With a gradient elution of petroleum ether−EtOAc from 20:1 to 1:2, the extract was fractionated through silica gel CC, giving fractions A−H. Bioactive fraction C (3.5 g, MIC = 78 *μ*g/mL for *S. aureus* ATCC 25923) was further submitted to ODS CC with MeOH−H_2_O as the mobile phase to afford 11 subfractions C1−C11 and fraction C9 was purified by Sephadex LH-20 CC (MeOH−CH_2_Cl_2_, 1:1) to gave six subfractions. With Shimadzu LC-8A preparative HPLC system using MeOH−H_2_O (75:25, 0.1% HCOOH) as the mobile phase, compounds **8** (42.3 mg, *t*_R_ 31.8 min), **3** (55.6 mg, *t*_R_ 33.7 min), **2** (13.5 mg, *t*_R_ 35.6 min), **1** (650.3 mg, *t*_R_ 38.8 min), and **4** (7.8 mg, *t*_R_ 48.2 min) were isolated from the third subfraction. Fraction C8 was purified likewise by Sephadex LH-20 CC and preparative HPLC using acetonitrile−H_2_O (61:39, 0.1% HCOOH) to give **7** (14.9 mg, *t*_R_ 14.7 min), **9** (4.7 mg, *t*_R_ 26.6 min), **6** (14.6 mg, *t*_R_ 31.4 min), and **5** (128.5 mg, *t*_R_ 33.1 min), respectively.

### Spectroscopic data

Neosartorin (**1**): Yellow powder; 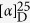 –210.2 (*c* 0.16, MeOH); UV (MeOH) *λ*_max_ (log *ε*) 208 (4.42), 334 (4.43) nm; ECD (3.9 × 10^−4^ M, MeOH) *λ* (Δ*ε*) 200 (−23.1), 219 (+7.9), 237 (−24.1), 316 (+14.7), 346 (−11.8) nm; ^1^H NMR (500 MHz, CDCl_3_) *δ* 13.91 (1 H, s, 8-OH), 13.78 (1 H, s, 8′-OH), 11.54 (1 H, s, 1-OH), 11.37 (1 H, s, 1′-OH), 7.12 (1 H, d, *J* = 8.5 Hz, H-3′), 6.58 (1 H, d, *J* = 8.5 Hz, H-2′), 6.47 (1 H, s, H-4), 5.26 (1 H, d, *J* = 1.3 Hz, H-5′), 4.36 (1 H, dd, *J* = 4.0, 1.5 Hz, H-5), 3.79 (3 H, s, OCH_3_-12), 3.65 (3 H, s, OCH_3_-12′), 2.84 (1 H, ddd, *J* = 18.6, 11.2, 6.9 Hz, H-7a), 2.39 (1 H, m, H-7b), 2.37 (2 H, m, H-7′), 2.31 (1 H, m, H-6′), 2.16 (1 H, m, H-6a), 2.08 (3 H, s, CH_3_-3), 1.98 (1 H, m, H-6b), 1.92 (3 H, s, CH_3_-14′), 0.92 (1 H, d, *J* = 5.8 Hz, CH_3_-13′); ^13^C NMR (125 MHz, CDCl_3_) *δ* 187.7 (C-9′a), 187.4 (C-9a), 178.7 (C-8), 177.7 (C-8′), 171.2 (C-11), 170.6 (C-11′), 169.0 (C-15′), 161.7 (C-1′), 159.8 (C-1), 156.7 (C-4a), 155.5 (C-4a′), 148.5 (C-3), 139.9 (C-3′), 118.6 (C-2), 114.6 (C-4′), 110.2 (C-2′), 108.9 (C-4), 106.8 (C-9′b), 104.8 (C-9b), 100.5 (C-9), 100.2 (C-9′), 83.9 (C-10), 82.1 (C-10′), 69.4 (C-5′), 67.0 (C-5), 53.6 (C-12), 53.4 (C-12′), 32.8 (C-7′), 27.8 (C-6′), 24.3 (C-7), 23.1 (C-6), 21.1 (3-CH_3_), 20.3 (C-14′), 17.0 (C-13′); ESIMS *m*/*z* 681.30 [M + H]^+^.

Lentulin A (**2**): Yellow powder; 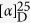 +276.1 (*c* 0.09, MeOH); UV (MeOH) *λ*_max_ (log *ε*) 205 (4.61), 337 (4.54) nm; ECD (3.9 × 10^−4^ M, MeOH) *λ* (Δ*ε*) 200 (−22.1), 217 (−53.9), 246 (+15.7), 306 (−2.0), 342 (+19.5) nm; IR (KBr) *ν*_max_ 3451, 1740, 1622, 1453, 1222 cm^−1^; ^1^H NMR and ^13^C NMR data, see Supporting Information Tables S1 and S2; ESIMS *m*/*z* 681.20 [M + H]^+^; HRESIMS *m*/*z* 703.1631 [M + Na]^+^ (calcd for 703.1633, C_34_H_32_O_15_Na).

Lentulin B (**3**): Yellow powder; 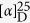 −319.4 (*c* 0.14, MeOH); UV (MeOH) *λ*_max_ (log *ε*) 205 (5.04), 334 (5.03) nm; ECD (3.9 × 10^−4^ M, MeOH) *λ* (Δ*ε*) 200 (−1.8), 220 (+29.5), 242 (−9.9), 316 (+7.3), 370 (−22.2) nm; IR (KBr) *ν*_max_ 3451, 2955, 1743, 1622, 1452, 1366, 1218 cm^−1^; ^1^H NMR and ^13^C NMR data, see Supporting Information Tables S1 and S2; ESIMS *m*/*z* 681.20 [M + H]^+^; HRESIMS *m*/*z* 703.1632 [M + Na]^+^ (calcd for 703.1633, C_34_H_32_O_15_Na).

Lentulin C (**4**): Yellow powder; 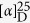 +69.0 (*c* 0.09, MeOH); UV (MeOH) *λ*_max_ (log *ε*) 203 (4.80), 335 (4.69) nm; ECD (3.9 × 10^−4^ M, MeOH) *λ* (Δ*ε*) 200 (−2.9), 217 (−32.0), 241 (+18.2), 310 (−4.5), 343 (+17.1) nm; IR (KBr) *ν*_max_ 3458, 1735, 1625, 1458, 1384, 1220 cm^−1^; ^1^H NMR and ^13^C NMR data, see Supporting Information Tables S1 and S2; ESIMS *m*/*z* 681.11 [M + H]^+^; HRESIMS *m*/*z* 703.1637 [M + Na]^+^ (calcd for 703.1633, C_34_H_32_O_15_Na).

Lentulin D (**5**): Yellow powder; 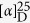 −114.3 (*c* 0.10, MeOH); UV (MeOH) *λ*_max_ (log *ε*) 205 (4.92), 278 (4.57), 336 (4.55) nm; ECD (3.9 × 10^−4^ M, MeOH) *λ* (Δ*ε*) 200 (−7.4), 213 (+6.3), 237 (−12.7), 284 (+3.6), 363 (−4.3) nm; IR (KBr) *ν*_max_ 3455, 2959, 1791, 1746, 1628, 1453, 1368, 1218 cm^−1^; ^1^H NMR and ^13^C NMR data, see Supporting Information Tables S1 and S2; ESIMS *m*/*z* 681.30 [M + H]^+^; HRESIMS *m*/*z* 703.1636 [M + Na]^+^ (calcd for 703.1633, C_34_H_32_O_15_Na).

Lentulin E (**6**): Yellow powder; 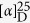 −211.4 (*c* 0.10, MeOH); UV (MeOH) *λ*_max_ (log *ε*) 205 (5.00), 278 (4.65), 338 (4.67) nm; ECD (3.9 × 10^−4^ M, MeOH) *λ* (Δ*ε*) 200 (−0.3), 215 (+18.0), 238 (−14.5), 352 (−2.6), 387 (+1.1) nm; IR (KBr) *ν*_max_ 3451, 2962, 1791, 1750, 1630, 1592, 1479, 1385, 1220 cm^−1^; ^1^H NMR and ^13^C NMR data, see Supporting Information Tables S1 and S2; ESIMS *m*/*z* 698.09 [M + NH_4_]^+^; HRESIMS *m*/*z* 703.1627 [M + Na]^+^ (calcd for 703.1633, C_34_H_32_O_15_Na).

Lentulin F (**7**): Yellow powder; 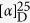 −111.7 (*c* 0.13, MeOH); UV (MeOH) *λ*_max_ (log *ε*) 205 (5.05), 278 (4.70), 335 (4.72) nm; ECD (3.9 × 10^−4^ M, MeOH) *λ* (Δ*ε*) 200 (−8.5), 213 (+6.8), 236 (−19.5), 282 (+6.5), 345 (−3.6), 379 (+0.9) nm; IR (KBr) *ν*_max_ 3455, 1743, 1631, 1478, 1385, 1220 cm^−1^; ^1^H NMR and ^13^C NMR data, see Supporting Information Tables S1 and S2; ESIMS *m*/*z* 699.04 [M + H]^+^; HRESIMS *m*/*z* 721.1736 [M + Na]^+^ (calcd for 721.1739, C_34_H_34_O_16_Na).

Lentulin G (**8**): Yellow powder; 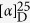 −159.5 (*c* 0.16, MeOH); UV (MeOH) *λ*_max_ (log *ε*) 205 (4.82), 276 (4.45), 346 (4.38) nm; ECD (3.9 × 10^−4^ M, MeOH) *λ* (Δ*ε*) 200 (−4.1), 213 (+4.8), 236 (−8.2), 282 (+2.8), 368 (−1.6) nm; IR (KBr) *ν*_max_ 3461, 2957, 1741, 1625, 1418, 1367, 1217 cm^−1^; ^1^H NMR and ^13^C NMR data, see Supporting Information Tables S1 and S2; ESIMS *m*/*z* 713.20 [M + H]^+^; HRESIMS *m*/*z* 735.1893 [M + Na]^+^ (calcd for 735.1896, C_35_H_36_O_16_Na).

Lentulin H (**9**): Yellow powder; 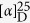 −161.5 (*c* 0.07, MeOH); UV (MeOH) *λ*_max_ (log *ε*) 205 (4.88), 277 (4.52), 347 (4.51) nm; ECD (3.9 × 10^−4^ M, MeOH) *λ* (Δ*ε*) 213 (+12.1), 238 (−8.8), 286 (−0.7), 349 (−3.0), 387 (+1.1) nm; IR (KBr) *ν*_max_ 3446, 2956, 1741, 1625, 1588, 1452, 1368, 1217 cm^−1^; ^1^H NMR and ^13^C NMR data, see Supporting Information Tables S1 and S2; ESIMS *m*/*z* 713.08 [M + H]^+^; HRESIMS *m*/*z* 735.1899 [M + Na]^+^ (calcd for 735.1896, C_35_H_36_O_16_Na).

### Absolute configuration of 5-secondary alcohol in 4

To a CH_2_Cl_2_ solution of **4** (0.5 mg/mL), 0.8 mg of Rh_2_(OCOCF_3_)_4_ was added and the first induced ECD spectrum was recorded immediately. The following spectra were measured every 5 minutes until reaching a stationary state. The absolute configuration of 5-sencondary alcohol was determined by the induced CE at around 350 nm.

### Conversion of neosartorin (1) and lentulin B (3) into lentulin D (5) and lentulin E (6)

To solutions of **1** and **3** (each 1.5 mg, 0.0022 mmol) in 2 mL CH_2_Cl_2_, triethylamine (10 *μ*L, 0.072 mmol) and 1-(3-dimethylaminopropyl)-3-ethylcarbodiimide hydrochloride (EDC·HCl, 2.0 mg, 0.010 mmol) were added separately. The solutions were stirred at room temperature for 24 h. The reactions were quenched by 2 mL of 0.2 M HCl and the mixtures were extracted with EtOAc three times. Then, the EtOAc layers were washed by water and brine. After being evaporated under gas flow, the residues were purified over Shimadzu LC-8A preparative HPLC system with acetonitrile−H_2_O (61:39, 0.1% HCOOH) as the mobile phase. Synthetic **5** and **6** were obtained at *t*_R_ 33.1 min and *t*_R_ 31.4 min, respectively. All the spectral data of the synthetic products were the same as the natural products including their optical rotations {synthetic **5**, 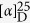 −108.6 (*c* 0.11, MeOH); natural **5**, 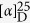 −114.9 (*c* 0.13, MeOH); synthetic **6**, 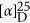 −207.0 (*c* 0.12, MeOH); natural **6**, 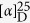 −211.4 (*c* 0.10, MeOH)}.

### Methanolysis of lentulin D (5) and lentulin E (6)

Compounds **5** (2.0 mg, 0.0029 mmol) and **6** (1.5 mg, 0.0022 mmol) were dissolved in 0.5% HCl−MeOH (1.0 mL) and were stirred at room temperature for 6 h, respectively^22^. The residues were obtained by evaporating the solutions under gas flow, and were chromatographied by preparative HPLC to give synthetic **8** (1.5 mg, 0.0021 mmol, 72%, *t*_R_ 31.8 min in 75% MeOH with 0.1% HCOOH) and synthetic **9** (1.2 mg, 0.0017 mmol, 76%, *t*_R_ 26.6 min in 61% acetonitrile with 0.1% HCOOH), respectively. All the spectral data of the synthetic **8** and **9** were the same as the natural products {synthetic **8**, 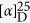 −148.3 (*c* 0.08, MeOH); natural **8**, 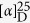 −159.5 (*c* 0.16, MeOH); synthetic **9**, 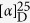 −174.5 (*c* 0.07, MeOH); natural **9**, 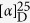 −161.5 (*c* 0.07, MeOH)}.

### Mosher esterfication of lentulins G (8) and H (9)

To 2 mL of CH_2_Cl_2_ solution of **8** (2.1 mg, 0.0029 mmol), triethylamine (10 *μ*L, 0.072 mmol), DMAP (0.2 mg, 0.0016 mmol), EDC·HCl (4.6 mg, 0.024 mmol), and (*R*)-MPA (3.5 mg, 0.021 mmol) were added respectively^22^. The mixture was poured into 2 mL of 0.2 M HCl after being stirred for 24 h at room temperature and was extracted with EtOAc three times. After washing by water and brine, the EtOAc layer was evaporated to give a residue, which was finally purified using preparative HPLC with MeOH−H_2_O (83:17, 0.1% HCOOH) to yield **8a** (1.0 mg, 0.0012 mmol, 40%, *t*_R_ 22.1 min). In the same manner, **8b** (0.8 mg, 0.00093 mmol, 39%, *t*_R_ 23.7 min) was obtained by preparative HPLC (83% MeOH with 0.1% HCOOH) from 1.7 mg of **8**. Subsequently, **9a** (0.4 mg, 0.00046 mmol, 36%, *t*_R_ 25.2 min) and **9b** (0.5 mg, 0.00058 mmol, 38%, *t*_R_ 26.3 min) were yielded from 1.0 mg of **9** using preparative HPLC (81% MeOH with 0.1% HCOOH).

Data for **lentulin G (*****R*****)-MPA ester 8a:** δ_H_ 13.820 (1 H, s, 8′-OH), 11.720 (1 H, s, 1-OH), 11.387 (1 H, s, 1′-OH), 7.317−7.438 (5 H, aromatic), 7.120 (1 H, d, *J* = 7.7 Hz, H-3′), 6.591 (1 H, d, *J* = 8.1 Hz, H-2′), 6.433 (1 H, s, H-4), 5.506 (1 H, dd, *J* = 9.3, 2.3 Hz, H-5), 5.269 (1 H, br d, H-5′), 4.777 (1 H, s, H-MPA), 3.803 (3 H, s, OCH_3_-12), 3.643 (3 H, s, OCH_3_-12′), 3.616 (3 H, s, OCH_3_-13), 3.420 (3 H, s, OCH_3_-MPA), 3.158 (1 H, d, *J* = 16.9 Hz, H-9a), 3.023 (1 H, d, *J* = 17.0 Hz, H-9b), 2.353 (2 H, m, H-7′), 2.312 (1 H, m, H-6′), 2.175 (1 H, d, *J* = 5.4 Hz, H-7a), 2.162 (1 H, d, *J* = 7.6 Hz, H-7b), 2.071 (3 H, s, CH_3_-3), 1.936 (3 H, s, CH_3_-14′), 1.923−1.907 (2 H, overlap, H-6), 0.923 (1 H, d, *J* = 6.6 Hz, CH_3_-13′); ESI-MS: m/z 878.09 [M + NH_4_]^+^, m/z 895.11 [M + Cl]^−^.

Data for **lentulin G (*****S*****)-MPA ester 8b:** δ_H_ 13.792 (1 H, s, 8′-OH), 11.637 (1 H, s, 1-OH), 11.385 (1 H, s, 1′-OH), 7.320−7.385 (5 H, aromatic), 7.131 (1 H, d, *J* = 7.7 Hz, H-3′), 6.603 (1 H, d, *J* = 8.2 Hz, H-2′), 6.275 (1 H, s, H-4), 5.520 (1 H, dd, *J* = 9.6, 2.3 Hz, H-5), 5.245 (1 H, br d, H-5′), 4.797 (1 H, s, H-MPA), 3.703 (3 H, s, OCH_3_-12), 3.696 (3 H, s, OCH_3_-13), 3.641 (3 H, s, OCH_3_-12′), 3.425 (3 H, s, OCH_3_-MPA), 2.837 (1 H, d, *J* = 16.8 Hz, H-9a), 2.594 (1 H, d, *J* = 17.0 Hz, H-9b), 2.371 (1 H, d, *J* = 7.2 Hz, H-7a), 2.350 (2 H, m, H-7′), 2.349 (1 H, d, *J* = 6.6 Hz, H-7b), 2.308 (1 H, m, H-6′), 2.058 (3 H, s, CH_3_-3), 1.882 (3 H, s, CH_3_-14′), 2.047−1.994 (2 H, overlap, H-6), 0.908 (1 H, d, *J* = 6.0 Hz, CH_3_-13′); ESI-MS: m/z 878.09 [M + NH_4_]^+^, m/z 895.03 [M + Cl]^−^.

Data for **lentulin H (*****R*****)-MPA ester 9a:** δ_H_ 13.938 (1 H, s, 8′-OH), 11.674 (1 H, s, 1-OH), 11.402 (1 H, s, 1′-OH), 7.340−7.408 (5 H, aromatic), 7.155 (1 H, d, *J* = 7.7 Hz, H-3′), 6.594 (1 H, d, *J* = 8.8 Hz, H-2′), 6.461 (1 H, s, H-4), 5.425 (1 H, dd, *J* = 8.4, 2.9 Hz, H-5), 5.297 (1 H, br d, H-5′), 4.856 (1 H, s, H-MPA), 3.687 (3 H, s, OCH_3_-12), 3.636 (3 H, s, OCH_3_-12′), 3.612 (3 H, s, OCH_3_-13), 3.432 (3 H, s, OCH_3_-MPA), 3.421 (1 H, d, *J* = 16.4 Hz, H-9a), 3.304 (1 H, d, *J* = 16.4 Hz, H-9b), 3.093 (2 H, m, H-7), 2.390 (2 H, m, H-7′), 2.342 (1 H, m, H-6′), 2.053 (3 H, s, CH_3_-3), 2.014 (3 H, s, CH_3_-14′), 1.956−1.815 (2 H, overlap, H-6), 0.938 (1 H, d, *J* = 6.0 Hz, CH_3_-13′); ESI-MS: m/z 861.19 [M + H]^+^.

Data for **lentulin H (*****S*****)-MPA ester 9b:** δ_H_ 13.919 (1 H, s, 8′-OH), 11.613 (1 H, s, 1-OH), 11.392 (1 H, s, 1′-OH), 7.340−7.537 (5 H, aromatic), 7.131 (1 H, d, *J* = 9.3 Hz, H-3′), 6.579 (1 H, d, *J* = 8.5 Hz, H-2′), 6.294 (1 H, s, H-4), 5.427 (1 H, dd, *J* = 9.8, 3.9 Hz, H-5), 5.295 (1 H, br d, H-5′), 4.815 (1 H, s, H-MPA), 3.701 (3 H, s, OCH_3_-13), 3.636 (3 H, s, OCH_3_-12′), 3.515 (3 H, s, OCH_3_-12), 3.494 (2 H, m, H-7), 3.443 (3 H, s, OCH_3_-MPA), 2.890 (1 H, d, *J* = 15.7 Hz, H-9a), 2.784 (1 H, d, *J* = 15.7 Hz, H-9b), 2.381 (2 H, m, H-7′), 2.360 (1 H, m, H-6′), 2.022 (3 H, s, CH_3_-3), 1.978 (3 H, s, CH_3_-14′), 1.964−1.879 (2 H, overlap, H-6), 0.946 (1 H, d, *J* = 6.0 Hz, CH_3_-13′); ESI-MS: m/z 861.20 [M + H]^+^.

### Conversion of lentulin F (7) into lentulin D (5)

A sample of **7** (1.8 mg, 0.0026 mmol) was dissolved in 2 mL of CH_2_Cl_2_, followed by addition of triethylamine (10 *μ*L, 0.072 mmol) and EDC·HCl (2.0 mg, 0.010 mmol)^17^. The other experimental details were the same as above. Using Shimadzu LC-8A preparative HPLC system with acetonitrile−H_2_O (61:39, 0.1% HCOOH) as the mobile phase, synthetic **5** (1.4 mg, 0.0021 mmol, 80%) was obtained at 33.1 min. All the spectral data of the product were the same as natural **5** including their optical rotation {synthetic **5**, 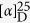 −104.7 (*c* 0.14, MeOH); natural **5**, 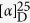 −114.9 (*c* 0.13, MeOH)}.

### Antimicrobial assays

Using broth microdilution method, the antimicrobial activities were measured in sterile 96-well plates (Thermo, USA) against five strains of Gram-positive bacteria (*S. aureus* ATCC 25923, *S. aureus* clinical strain, MRSA, MRSE, *Bacillus subtilis* ATCC 6633), four strains of Gram-negative bacteria (*Pseudomonas aeruginosa* ATCC 9027, *Escherichia coli* ATCC 25922, *Klebsiella pneumonia* clinical strain, *Acinetobacter baumannii* clinical strain), as well as one fungal strain (*Candida albicans* ATCC 24433). MIC values were determined by comparing OD values with the growth control through a microplate reader, using penicillin G, streptomycin, tigecycline, and fluconazole as the positive controls.

## Additional Information

**How to cite this article**: Li, T.-X. *et al*. Unusual dimeric tetrahydroxanthone derivatives from *Aspergillus lentulus* and the determination of their axial chiralities. *Sci. Rep.* **6**, 38958; doi: 10.1038/srep38958 (2016).

**Publisher's note:** Springer Nature remains neutral with regard to jurisdictional claims in published maps and institutional affiliations.

## Supplementary Material

Supplementary Information

## Figures and Tables

**Table 1:** MIC values (*μ*g/mL) of compounds against a panel of bacteria.

Compound^a^	*Sa*1^c^	*Sa*2^c^	MRSA^c^	MRSE^c^	*Pa*^c^	*Kp*^c^	*Ab*^c^
**1**	6.8 ± 0.6	>100	38.5 ± 4.3	31.5 ± 3.7	>100	62.8 ± 4.0	63.2 ± 6.3
**2**	4.7 ± 0.6	84.1 ± 9.9	65.8 ± 3.9	65.7 ± 6.0	35.8 ± 4.5	>100	>100
**3**	5.5 ± 0.4	3.1 ± 0.3	29.3 ± 2.3	31.3 ± 3.4	8.0 ± 0.6	59.1 ± 5.1	60.1 ± 4.2
**5**	13.3 ± 1.5	>100	62.5 ± 5.3	>100	>100	>100	>100
**6**	10.6 ± 0.4	40.2 ± 0.9	>100	>100	19.3 ± 0.6	>100	>100
penicillin^b^	0.5 ± 0.05	71.2 ± 6.0					
streptomycin^b^					0.6 ± 0.03		
tigecycline^b^			0.5 ± 0.03	0.7 ± 0.04		0.5 ± 0.05	0.6 ± 0.03

^a^The compounds were inactive when MICs > 100 *μ*g/mL, ±SD values were calculated on three individual experiments.

^b^Positive control.

^c^*Sa*1, *Staphylococcus aureus* ATCC 25923; *Sa*2, *S. aureus* clinical strain; *Pa*, *Pseudomonas aeruginosa* ATCC 9027; *Kp, Klebsiella pneumonia* clinical strain; *Ab, Acinetobacter baumannii* clinical strain.

## References

[b1] Stewart M. . Rugulotrosins A and B: two new antibacterial metabolites from an Australian isolate of a *Penicillium* sp. J Nat Prod 67, 728–730 (2004). 15104517 10.1021/np034038b

[b2] Rezanka T. & Sigler K. Hirtusneanoside, an unsymmetrical dimeric tetrahydroxanthone from the lichen *Usnea hirta*. J Nat Prod 70, 1487–1491 (2007). 17822296 10.1021/np070079m

[b3] Chutrakul C. . Ascherxanthone B from *Aschersonia luteola*, a new antifungal compound active against rice blast pathogen *Magnaporthe grisea*. J Appl Microbial 107, 1624–1631 (2009). 10.1111/j.1365-2672.2009.04349.x 19457038

[b4] Isaka M., Palasarn S., Kocharin K. & Saenboonrueng J. A cytotoxic xanthone dimer from the entomopathogenic fungus *Aschersonia* sp. BCC 8401. J Nat Prod 68, 945–946 (2005). 15974626 10.1021/np058028h

[b5] Masters K.-S. & Bräse S. Xanthones from fungi, lichens, and bacteria: the natural products and their synthesis. Chem Rev 112, 3717–3776 (2012). 22617028 10.1021/cr100446h

[b6] Tabata N., Tomoda H., Matsuzaki K. & Omura S. Structure and biosynthesis of xanthoquinodins, anticoccidial antibiotics. J Am Chem Soc 115, 8558–8564 (1993).

[b7] Yang D.-M., Takeda N., Iitaka Y., Sankawa V. & Shibata S. The structures of eumitrins A 1, A 2 and B: the yellow pigments of the lichen, *Usnea bayleyi* (Stirt.) Zahlbr. Tetrahedron 29, 519–528 (1973).

[b8] Proksa B., Uhrin D., Liptaj T. & Šturdíková M. Neosartorin, an ergochrome biosynthesized by *Neosartorya fischeri*. Phytochemistry 48, 1161–1164 (1998).

[b9] Smyth J. E., Butler N. M. & Keller P. A. A twist of nature–the significance of atropisomers in biological systems. Nat Prod Rep 32, 1562–1583 (2015). 26282828 10.1039/c4np00121d

[b10] Bringmann G., Gulder T., Gulder T. A. & Breuning M. Atroposelective total synthesis of axially chiral biaryl natural products. Chem Rev 111, 563–639 (2010). 20939606 10.1021/cr100155e

[b11] LaPlante S. R. . Assessing atropisomer axial chirality in drug discovery and development. J Med Chem 54, 7005–7022 (2011). 21848318 10.1021/jm200584g

[b12] Wagenaar M. M. & Clardy J. Dicerandrols, new antibiotic and cytotoxic dimers produced by the fungus *Phomopsis longicolla* isolated from an endangered mint. J Nat Prod 64, 1006–1009 (2001). 11520215 10.1021/np010020u

[b13] Isaka M. . Phomoxanthones A and B, novel xanthone dimers from the endophytic fungus *Phomopsis* species. J Nat Prod 64, 1015–1018 (2001). 11520217 10.1021/np010006h

[b14] Elsässer B. . X‐ray structure determination, absolute configuration and biological activity of phomoxanthone A. Eur J Org Chem 2005, 4563–4570 (2005).

[b15] Rönsberg D. . Pro-apoptotic and immunostimulatory tetrahydroxanthone dimers from the endophytic fungus *Phomopsis longicolla*. J Org Chem 78, 12409–12425 (2013). 24295452 10.1021/jo402066b

[b16] Ola A. R. . Absolute configuration and antibiotic activity of neosartorin from the endophytic fungus *Aspergillus fumigatiaffinis*. Tetrahedron Lett 55, 1020–1023 (2014).

[b17] Kikuchi H., Isobe M., Kurata S., Katou Y. & Oshima Y. New dimeric and monomeric chromanones, gonytolides D–G, isolated from the fungus *Gonytrichum* sp. Tetrahedron 68, 6218–6223 (2012). 10.1021/ol2018449 21827134

[b18] Zhang W. . New mono‐and dimeric members of the secalonic acid family: blennolides A–G isolated from the fungus *Blennoria* sp. Chem Eur J 14, 4913–4923 (2008). 18425741 10.1002/chem.200800035

[b19] Qin T. . Atropselective syntheses of (−) and (+) rugulotrosin A utilizing point-to-axial chirality transfer. Nat Chem 7, 234–240 (2015). 25698333 10.1038/nchem.2173 PMC4339264

[b20] Bara R. . Atropisomeric dihydroanthracenones as inhibitors of multiresistant *Staphylococcus aureus*. J Med Chem 56, 3257–3272 (2013). 23534483 10.1021/jm301816a

[b21] Gerards M. & Snatzke G. Circular dichroism, XCIII determination of the absolute configuration of alcohols, olefins, epoxides, and ethers from the CD of their “*in situ*” complexes with [Rh_2_(COOCF_3_)_4_]. Tetrahedron: Asymmetry 1, 221–236 (1990).

[b22] Kikuchi H. . Structures of the dimeric and monomeric chromanones, gonytolides A–C, isolated from the fungus *Gonytrichum* sp. and their promoting activities of innate immune responses. Org Lett 13, 4624–4627 (2011). 21827134 10.1021/ol2018449

[b23] Krick A. . Potential cancer chemopreventive *in vitro* activities of monomeric xanthone derivatives from the marine algicolous fungus *Monodictys putredinis*. J Nat Prod 70, 353–360 (2007). 17291041 10.1021/np060505o

[b24] Wezeman T., Bräse S. & Masters K. S. Xanthone dimers: a compound family which is both common and privileged. Nat Prod Rep 32, 6–28 (2015). 25226564 10.1039/c4np00050a

[b25] Bringmann G., Günther C., Ochse M., Schupp O. & Tasler S. Prog Chem Org Nat Prod 1–249 (Springer, 2001). 10.1007/978-3-7091-6227-9_1 11892255

[b26] Harada N. & Nakanishi K. Exciton chirality method and its application to configurational and conformational studies of natural products. Accounts Chem Res 5, 257–263 (1972).

[b27] Zhang H.-J., Zhang Y.-M., Luo J.-G., Luo J. & Kong L.-Y. Anti-inflammatory diterpene dimers from the root barks of *Aphanamixis grandifolia*. Org Biomol Chem 13, 7452–7458 (2015). 26062044 10.1039/c5ob00674k

[b28] Wu G.-W. . Versixanthones A–F, cytotoxic xanthone–chromanone dimers from the marine-derived fungus *Aspergillus versicolor* HDN1009. J Nat Prod 78, 2691–2698 (2015). 26506221 10.1021/acs.jnatprod.5b00636

[b29] Shim S. H., Baltrusaitis J., Gloer J. B. & Wicklow D. T. Phomalevones A−C: dimeric and pseudodimeric polyketides from a fungicolous Hawaiian isolate of *Phoma* sp.(Cucurbitariaceae). J Nat Prod 74, 395–401 (2011). 21247198 10.1021/np100791b

[b30] Bode S. E., Drochner D. & Müller M. Synthesis, biosynthesis, and absolute configuration of vioxanthin. Angew Chem 119, 6020–6024 (2007). 10.1002/anie.200701014 17607794

[b31] Isaka M., Yangchum A., Rachtawee P., Komwijit S. & Lutthisungneon A. Hopane-type triterpenes and binaphthopyrones from the scale insect pathogenic fungus *Aschersonia paraphysata* BCC 11964. J Nat Prod 73, 688–692 (2010). 20364867 10.1021/np1000363

[b32] Ohkawa Y. . Antiangiogenic metabolites from a marine-derived fungus, Hypocrea vinosa. J Nat Prod 73, 579–582 (2010). 20192239 10.1021/np900698p

[b33] Bruhn T. . Axially chiral BODIPY DYEmers: an apparent exception to the exciton chirality rule. Angew Chem. 53, 14592–14595 (2014). 25354119 10.1002/anie.201408398

[b34] Jurinovich S., Guido C. A., Bruhn T., Pescitelli G. & Mennucci B. The role of magnetic–electric coupling in exciton-coupled ECD spectra: the case of bis-phenanthrenes. Chem Commun 51, 10498–10501 (2015). 10.1039/c5cc03167b 26033039

[b35] Harada N., Nakanishi K. & Berova N. Electronic CD exciton chirality method: principles and applications. Compr Chiropt Spectrosc 2, 115–166 (2012).

[b36] Berova N., Di Bari L. & Pescitelli G. Application of electronic circular dichroism in configurational and conformational analysis of organic compounds. Chem Soc Rev 36, 914–931 (2007). 17534478 10.1039/b515476f

[b37] Li T.-X., Yang M.-H., Wang X.-B., Wang Y. & Kong L.-Y. Synergistic antifungal meroterpenes and dioxolanone derivatives from the endophytic Fungus *Guignardia* sp. J Nat Prod 78, 2511–2520 (2015). 26577190 10.1021/acs.jnatprod.5b00008

