# Supplementary material for: Unusual dimeric tetrahydroxanthone derivatives from Aspergillus lentulus and the determination of their axial chiralities
Source: Sci Rep. 2016 Dec 12;6:38958. doi: 10.1038/srep38958 (PMC5150534; doi:10.1038/srep38958)
Supplement: Supplementary Information [file srep38958-s1.pdf]

Supporting Information

Unusual dimeric tetrahydroxanthone derivatives from *Aspergillus lentulus* and the determination of their axial chiralities

Tian-Xiao Li,+ Ming-Hua Yang,+ Ying Wang, Xiao-Bing Wang, Jun Luo, Jian-Guang Luo & Ling-Yi Kong*

State Key Laboratory of Natural Medicines, Department of Natural Medicinal Chemistry, China Pharmaceutical University, 24 Tong Jia Xiang, Nanjing 210009, People's Republic of China

*Corresponding author.

Tel/Fax: +86-25-8327-1405.

E-mail address: cpu_lykong@126.com.

+These authors contributed equally to this work.

Content

Table S1. ^{13}C NMR (125 MHz) spectroscopic data for **2**–**9** 1

Table S2. ^{1}H NMR (500 MHz, *J* in Hz) spectroscopic data for **2**–**9** 2

Table S3. ECD CEs of reported dimeric tetrahydroxanthone analogues 3

Figure S1. $Rh_2(OCOCF_3)_4$-induced ECD spectrum of lentulin C (**4**) (in CH_2Cl_2) 4

Figure S2. ^{1}H NMR spectrum (500 MHz, $CDCl_3$) of neosartorin (**1**) 5

Figure S3. ^{13}C NMR spectrum (125 MHz, $CDCl_3$) of neosartorin (**1**) 5

Figure S4. ROESY spectrum ($CDCl_3$) of neosartorin (**1**) 6

Figure S5. UV spectrum (MeOH) of neosartorin (**1**) 6

Figure S6. HRESIMS spectrum of lentulin A (**2**) 7

Figure S7. ^{1}H NMR spectrum (500 MHz, $CDCl_3$) of lentulin A (**2**) 7

Figure S8. ^{13}C NMR spectrum (125 MHz, $CDCl_3$) of lentulin A (**2**) 8

Figure S9. HSQC spectrum ($CDCl_3$) of lentulin A (**2**) 8

Figure S10. HMBC spectrum ($CDCl_3$) of lentulin A (**2**) 9

Figure S11. ROESY spectrum ($CDCl_3$) of lentulin A (**2**) 9

Figure S12. UV spectrum (MeOH) of lentulin A (**2**) 10

Figure S13. HRESIMS spectrum of lentulin B (**3**) 10

Figure S14. ^{1}H NMR spectrum (500 MHz, $CDCl_3$) of lentulin B (**3**) 11

Figure S15. ^{13}C NMR spectrum (125 MHz, $CDCl_3$) of lentulin B (**3**) 11

Figure S16. HSQC spectrum ($CDCl_3$) of lentulin B (**3**) 12

Figure S17. HMBC spectrum ($CDCl_3$) of lentulin B (**3**) 12

Figure S18. ROESY spectrum ($CDCl_3$) of lentulin B (**3**) 13

Figure S19. UV spectrum (MeOH) of lentulin B (**3**) 13

Figure S20. HRESIMS spectrum of lentulin C (**4**) 14

Figure S21. ^{1}H NMR spectrum (500 MHz, $CDCl_3$) of lentulin C (**4**) 14

Figure S22. ^{13}C NMR spectrum (125 MHz, $CDCl_3$) of lentulin C (**4**) 15

Figure S23. HSQC spectrum ($CDCl_3$) of lentulin C (**4**) 15

Figure S24. HMBC spectrum ($CDCl_3$) of lentulin C (**4**) 16

Figure S25. ROESY spectrum ($CDCl_3$) of lentulin C (**4**) 16

Figure S26. UV spectrum (MeOH) of lentulin C (**4**) 17

Figure S27. HRESIMS spectrum of lentulin D (**5**) 17

Figure S28. ^{1}H NMR spectrum (500 MHz, $CDCl_3$) of lentulin D (**5**) 18

Figure S29. ^{13}C NMR spectrum (125 MHz, $CDCl_3$) of lentulin D (**5**) 18

Figure S30. HSQC spectrum ($CDCl_3$) of lentulin D (**5**) 19

Figure S31. HMBC spectrum ($CDCl_3$) of lentulin D (**5**) 19

Figure S32. ROESY spectrum ($CDCl_3$) of lentulin D (**5**) 20

Figure S33. UV spectrum (MeOH) of lentulin D (**5**) 20

Figure S34. HRESIMS spectrum of lentulin E (**6**) 21

Figure S35. ^{1}H NMR spectrum (500 MHz, $CDCl_3$) of lentulin E (**6**) 21

Figure S36. ^{13}C NMR spectrum (125 MHz, $CDCl_3$) of lentulin E (**6**) 22

Figure S37. HSQC spectrum ($CDCl_3$) of lentulin E (**6**) 22

Figure S38. HMBC spectrum ($CDCl_3$) of lentulin E (**6**) 23

Figure S39. ROESY spectrum ($CDCl_3$) of lentulin E (**6**) 23

Figure S40. UV spectrum (MeOH) of lentulin E (**6**) 24

Figure S41. HRESIMS spectrum of lentulin F (**7**) 24

Figure S42. ^{1}H NMR spectrum (500 MHz, Acetone-d_6) of lentulin F (**7**) 25

Figure S43. ^{13}C NMR spectrum (125 MHz, Acetone-d_6) of lentulin F (**7**) 25

Figure S44. HSQC spectrum (Acetone-d_6) of lentulin F (**7**) 26

Figure S45. HMBC spectrum (Acetone-d_6) of lentulin F (**7**) 26

Figure S46. ROESY spectrum (Acetone-d_6) of lentulin F (**7**) 27

Figure S47. UV spectrum (MeOH) of lentulin F (**7**) 27

Figure S48. HRESIMS spectrum of lentulin G (**8**) 28

Figure S49. ^{1}H NMR spectrum (500 MHz, $CDCl_3$) of lentulin G (**8**) 28

Figure S50. ^{13}C NMR spectrum (125 MHz, $CDCl_3$) of lentulin G (**8**) 29

Figure S51. HSQC spectrum ($CDCl_3$) of lentulin G (**8**) 29

Figure S52. HMBC spectrum ($CDCl_3$) of lentulin G (**8**) 30

Figure S53. ROESY spectrum ($CDCl_3$) of lentulin G (**8**) 30

Figure S54. UV spectrum (MeOH) of lentulin G (**8**) 31

Figure S55. ^{1}H NMR spectrum (500 MHz, $CDCl_3$) of lentulin G (*R*)-MPA ester **8a** 31

Figure S56. ^{1}H NMR spectrum (500 MHz, $CDCl_3$) of lentulin G (*S*)-MPA ester **8b** 32

Figure S57. HRESIMS spectrum of lentulin H (**9**) 32

Figure S58. ^{1}H NMR spectrum (500 MHz, $CDCl_3$) of lentulin H (**9**) 33

Figure S59. ^{13}C NMR spectrum (125 MHz, $CDCl_3$) of lentulin H (**9**) 33

Figure S60. HSQC spectrum ($CDCl_3$) of lentulin H (**9**) 34

Figure S61. HMBC spectrum ($CDCl_3$) of lentulin H (**9**) 34

Figure S62. ROESY spectrum ($CDCl_3$) of lentulin H (**9**) 35

Figure S63. UV spectrum (MeOH) of lentulin H (**9**) 35

Figure S64. ^{1}H NMR spectrum (500 MHz, $CDCl_3$) of lentulin H (*R*)-MPA ester **9a** 36

Figure S65. ^{1}H NMR spectrum (500 MHz, $CDCl_3$) of lentulin H (*S*)-MPA ester **9b** 36

Table S1. ^{13}C **NMR (125 MHz) Spectroscopic Data for 2–9**

position	**2**[a]	**3**[a]	**4**[a]	**5**[a]	**6**[a]	**7**[b]	**8**[a]	**9**[a]
1	159.5	159.9	159.5	159.9	159.8	161.0	159.8	159.7
2	118.6	118.1	118.1	118.0	117.8	118.8	118.0	117.4
3	149.6	149.0	149.5	149.8	149.5	150.6	149.4	149.4
4	108.7	109.1	108.8	108.7	108.7	109.9	108.5	108.5
4a	156.8	157.7	157.8	158.1	158.0	160.3	158.1	158.3
5	67.1	71.9	72.0	81.0	79.7	74.9	74.0	74.7
6	23.1	23.9	23.9	22.1	21.8	27.5	25.8	26.5
7	24.4	27.5	27.7	27.7	27.6	31.5	30.6	30.5
8	179.3	177.5	177.6	175.6	175.8	175.3	173.8	174.0
9	100.2	101.2	101.9	39.4	40.3	40.0	38.3	40.3
9a	187.2	186.8	187.0	193.8	194.4	198.1	195.7	195.4
9b	104.9	104.6	104.9	105.6	105.4	106.9	105.5	105.5
10	83.9	84.4	84.6	84.2	84.7	88.7	86.5	86.1
11	171.3	170.2	170.9	169.2	169.2	171.8	170.3	170.3
12	53.4	53.1	53.1	53.7	53.6	54.0	53.5	53.2
13							51.8	51.8
3-CH_3	21.0	21.3	21.1	21.2	21.3	22.0	21.2	21.2
1'	161.7	161.8	161.6	161.8	161.8	162.9	161.8	161.8
2'	111.0	110.5	110.8	110.4	110.4	111.1	110.3	110.4
3'	140.1	139.8	140.2	139.7	139.5	141.8	139.9	139.8
4'	115.0	114.8	114.9	114.5	114.6	116.7	114.6	114.7
4'a	154.6	155.2	154.8	155.3	155.3	157.3	155.5	155.3
5'	70.7	70.0	70.3	69.6	69.9	71.2	69.6	70.0
6'	27.8	27.7	28.0	27.7	27.6	29.3	27.8	27.7
7'	33.2	33.0	33.0	32.9	33.0	34.2	32.9	32.9
8'	178.4	178.0	178.1	178.0	178.3	180.1	177.8	177.9
9'	101.1	100.3	100.9	100.3	100.5	101.9	100.3	100.3
9'a	187.8	187.7	188.0	187.7	187.7	189.3	187.8	187.8
9'b	107.2	106.8	107.2	106.8	106.9	108.2	106.9	106.9
10'	82.7	82.3	82.4	82.3	82.5	84.0	82.2	82.3
11'	170.5	170.5	170.2	170.5	170.7	171.6	170.6	170.5
12'	53.6	53.3	53.4	53.3	53.3	54.4	53.3	53.3
13'	17.2	17.1	17.2	17.1	17.0	17.8	17.1	17.0
14'	20.8	20.6	20.5	20.4	20.5	21.0	20.3	20.5
15'	169.4	169.6	168.9	169.0	169.3	170.5	169.1	170.1

[a]measured in $CDCl_3$, [b]measured in Acetone-d_6

Table S2. ^{1}H NMR (500 MHz, *J* in Hz) Spectroscopic Data for 2–9

position	**2**[a]	**3**[a]	**4**[a]	**5**[a]	**6**[a]	**7**[b]	**8**[a]	**9**[a]
4	6.47, s	6.53, s	6.49, s	6.52, s	6.49, s	6.44, s	6.48, s	6.52, s
5	4.34, dd (4.0, 1.5)	4.35, dd (12.4, 4.8)	4.34, br s	4.89, dd (7.6, 6.3)	4.81, dd (8.5, 4.4)	4.12, d (10.1)	4.09, d (10.4)	3.98, d (8.4)
6	2.17, m	2.20, m	2.23, m	2.44, m	2.53, m	1.92, m	1.89, m	1.99, m
	1.98, m	2.11, m	2.09, m	2.44, m	2.34, m	1.73, m	1.75, m	1.95, m
7	2.86, m	2.65, m	2.67, m	2.71, ddd (17.0, 9.9, 6.7)	2.82, ddd (17.8,10.2, 6.8)	2.61, dt (17.0, 7.0)	2.61, dt (16.7, 6.9)	2.59, m
	2.40, m	2.65, m	2.67, m	2.60, ddd (17.2, 10.0, 6.9)	2.58, ddd (17.8, 10.2, 5.2)	2.51, dt (16.6, 7.5)	2.56, dt (16.7, 6.7)	2.59, m
				3.21, d (16.9)	3.55, d (16.9)	3.28, d (17.0)	3.20, d (17.2)	3.41, d (16.8)
				2.97, d (16.9)	3.07, d (16.9)	3.14, d (17.0)	3.15, d (17.2)	3.09, d (16.9)
12	3.74, s	3.74, s	3.80, s	3.81, s	3.76, s	3.79, s	3.83, s	3.77, s
13							3.70, s	3.71, s
1-OH	11.51, s	11.42, s	11.36, s	11.70, s	11.66, s	11.86, s	11.83, s	11.75, s
3-CH_3	1.97, s	2.04, s	1.97, s	2.08, s	2.06, s	2.10, s	2.08, s	2.05, s
8-OH	14.02, s	13.87, s	13.85, s					
2'	6.62, d (8.5)	6.59, d (8.5)	6.62, br s	6.58, d (8.5)	6.59, d (8.5)	6.55, d (7.6)	6.58, d (8.5)	6.60, d (8.5)
3'	7.19, d (8.5)	7.15, d (8.5)	7.20, br s	7.13, d (8.5)	7.17, d (8.5)	7.21, d (7.7)	7.12, d (8.5)	7.17, d (8.4)
5'	5.39, d (1.3)	5.34, br s	5.34, s	5.25, br s	5.29, d (1.2)	5.35, br s	5.28, d (1.1)	5.29, s
6'	2.15, m	2.24, m	2.15, m	2.26, m	2.24, m	2.36, m	2.29, m	2.24, m
7'	2.44, m	2.42, dd (18.8, 6.6)	2.40, m	2.38, m	2.38, m	2.42, m	2.37, m	2.38, m
	2.33, m	2.34, dd (18.8, 10.8)	2.32, m	2.38, m	2.38, m	2.42, m	2.37, m	2.38, m
12'	3.63, s	3.64, s	3.59, s	3.64, s	3.63, s	3.66, s	3.65, s	3.64, s
13'	0.94, d (6.7)	0.93, d (6.6)	0.93, br s	0.91, d (6.5)	0.92, d (6.7)	0.93, d (3.2)	0.92, d (6.3)	0.93, d (6.2)
14'	2.07, s	2.04, s	2.01, s	1.95, s	1.99, s	1.93, s	1.95, s	1.98, s
1'-OH	11.40, s	11.42, s	11.36, s	11.38, s	11.39, s		11.39, s	11.38, s
8'-OH	14.11, s	13.93, s	14.02, s	13.85, s	13.93, s		13.82, s	13.88, s

[a]measured in $CDCl_3$, [b]measured in Acetone-d_6

Table S3. ECD CEs of Reported Dimeric Tetrahydroxanthone Analogues

compound	axial linkage	chromophore	exciton chirality	1st[$\Delta\varepsilon$(nm)]	2nd[$\Delta\varepsilon$(nm)]	axial chirality	ref
phomoxanthone A	4,4′	1-arylpropenone	(−)	−7.8(341)	+17.7(316)	a*R*	1
gonytolide F	4,4′	benzoyl	(+)	+3.1(235)	−19.0(215)	a*S*	2
versixanthone A	2,4′	benzoyl	(−)	−128.1(253)	+135.5(219)	a*R*	3
phomalevone A	2,2′	benzoyl	(+)	+25.0(236)	−90.0(213)	a*S*	4
phomalevone B	2,2′	benzoyl	(+)	+12.0(242)	-41.0(216)	a*S*	4
phomalevone C	2,2′	benzoyl	(+)	+38.0(235)	−106.0(213)	a*S*	4

1. Rönsberg, D. *et al.* Pro-apoptotic and immunostimulatory tetrahydroxanthone dimers from the endophytic fungus *Phomopsis longicolla*. *J Org Chem* **78**, 12409-12425 (2013).
2. Kikuchi, H., Isobe, M., Kurata, S., Katou, Y. & Oshima, Y. New dimeric and monomeric chromanones, gonytolides D–G, isolated from the fungus *Gonytrichum* sp.. *Tetrahedron* **68**, 6218-6223 (2012).
3. Wu, G.-W. *et al.* Versixanthones A–F, cytotoxic xanthone–chromanone dimers from the marine-derived fungus *Aspergillus versicolor* HDN1009. *J Nat Prod* **78**, 2691-2698 (2015).
4. Shim, S. H., Baltrusaitis, J., Gloer, J. B. & Wicklow, D. T. Phomalevones A−C: dimeric and pseudodimeric polyketides from a fungicolous Hawaiian isolate of *Phoma* sp.(Cucurbitariaceae). *J Nat Prod* **74**, 395-401 (2011).

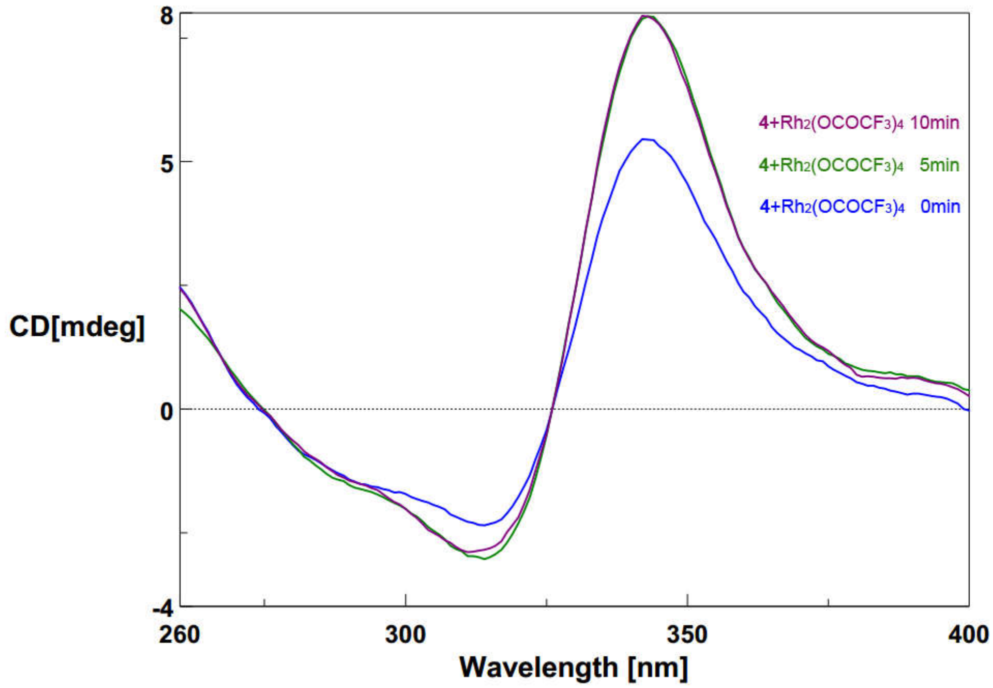

Figure S1. $Rh_2(OCOCF_3)_4$-induced ECD spectrum of lentulin C (**4**) (in CH_2Cl_2)

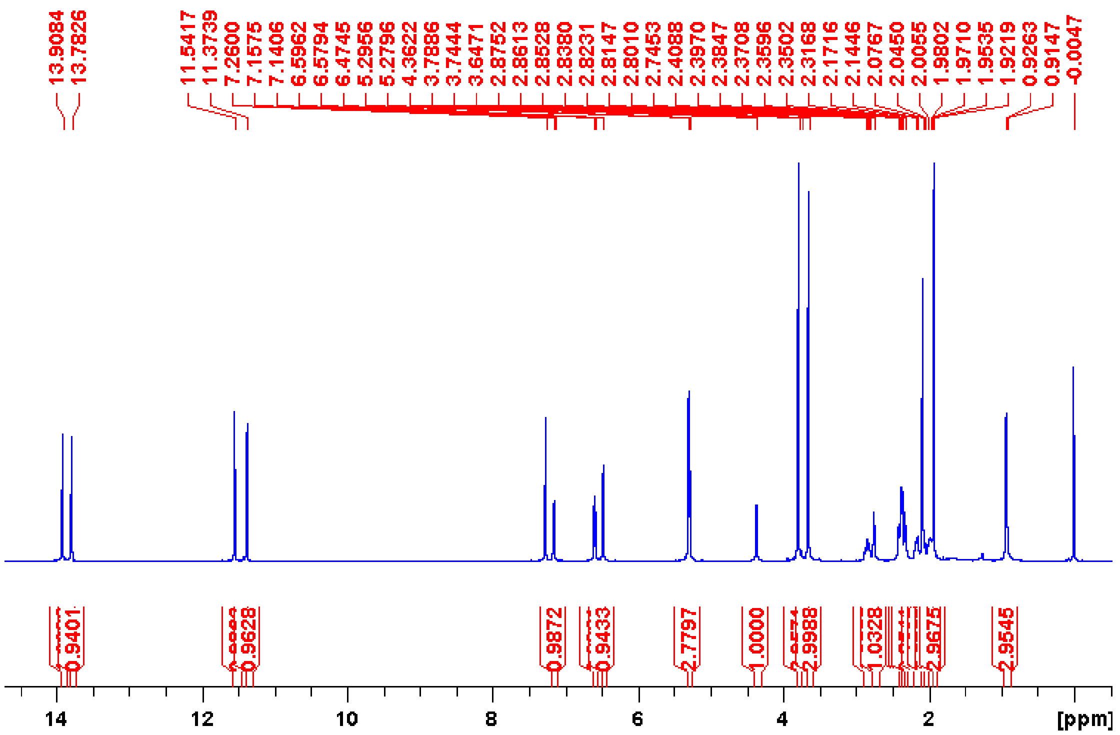

Figure S2. ^{1}H NMR spectrum (500 MHz, $CDCl_3$) of neosartorin (**1**)

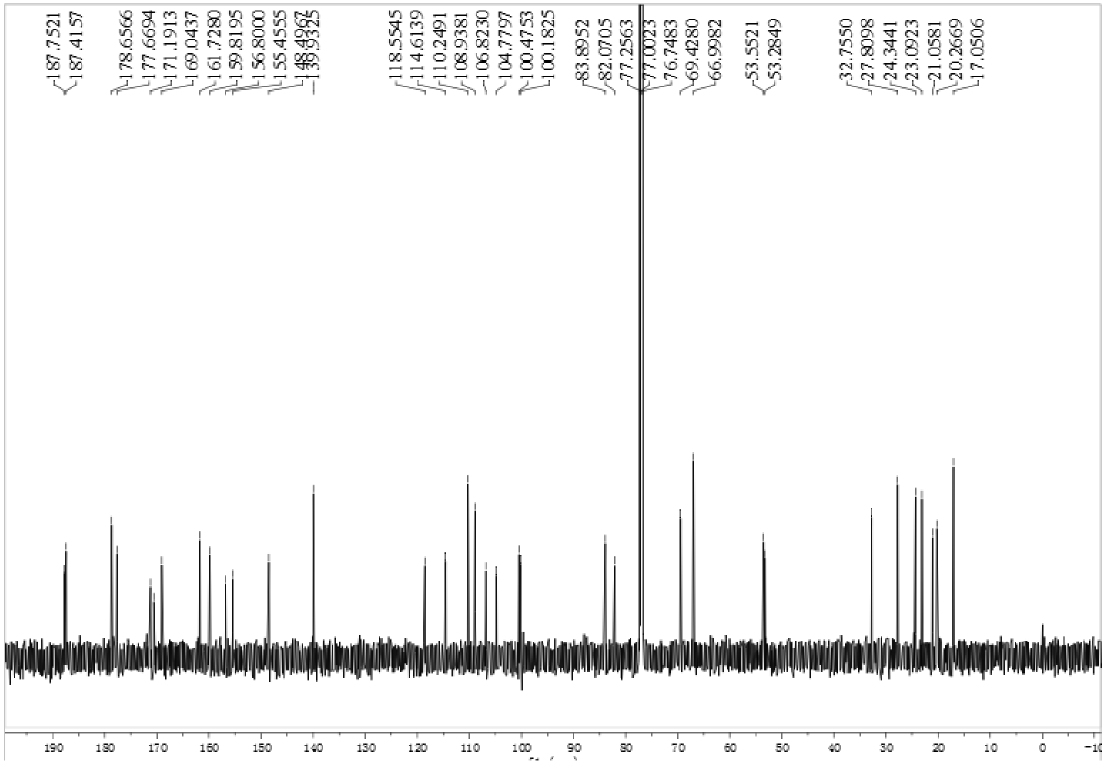

Figure S3. ^{13}C NMR spectrum (125 MHz, $CDCl_3$) of neosartorin (**1**)

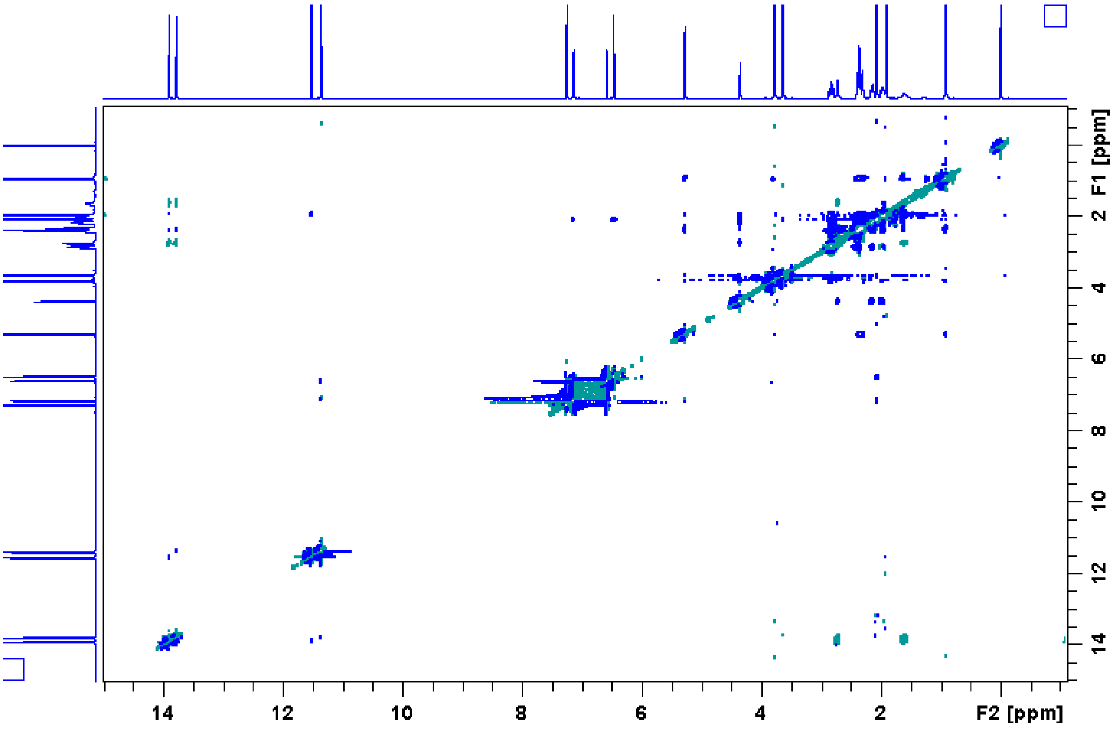

Figure S4. ROESY spectrum ($CDCl_3$) of neosartorin (**1**)

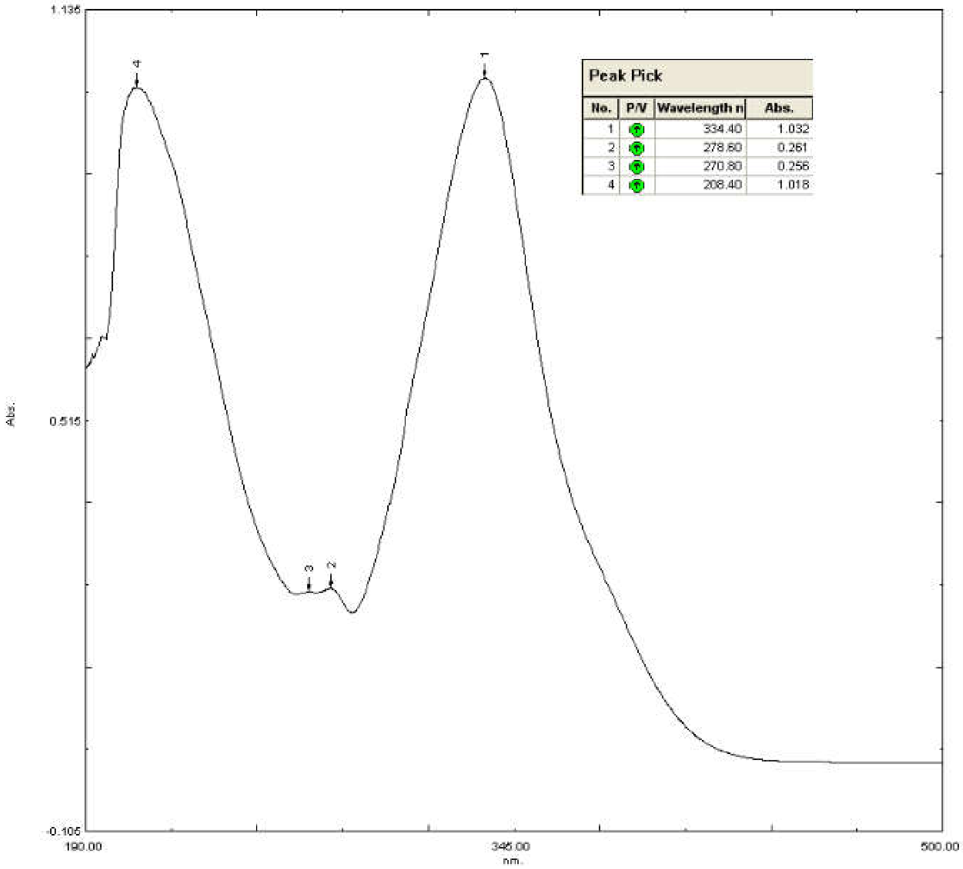

Figure S5. UV spectrum (MeOH) of neosartorin (**1**)

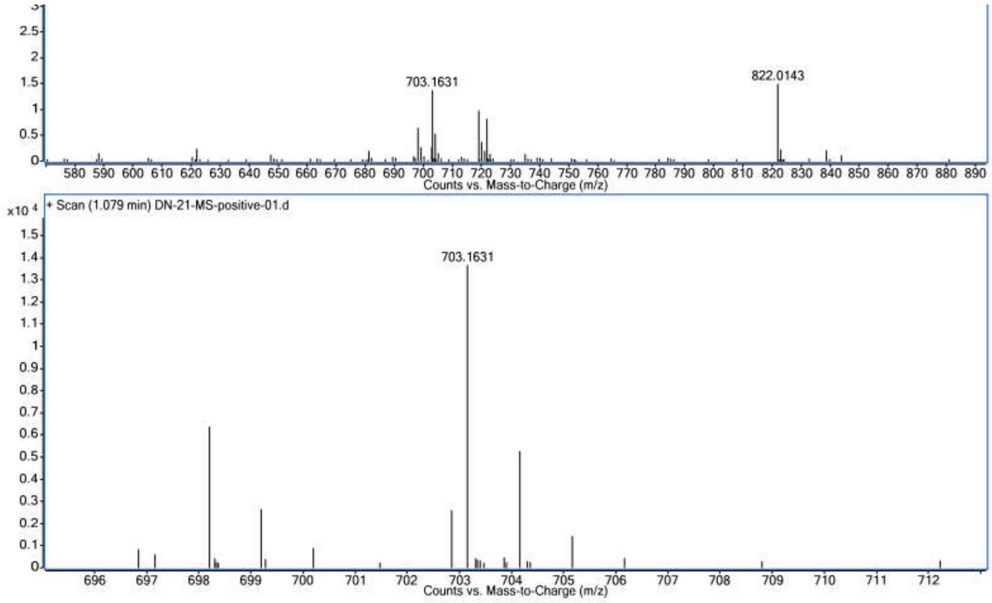

Elemental Composition Calculator

Target m/z:	703.1631	Result type:	Positive ions	Species:	[M+Na]+
Elements:		C (0-80); H (0-120); O (0-30); N(0-10); Na (0-5)			
Ion Formula		Calcalated m/z		PPM Error	
C34H32NaO15		703.1633		0.29	

Figure S6. HRESIMS spectrum of lentulin A (**2**)

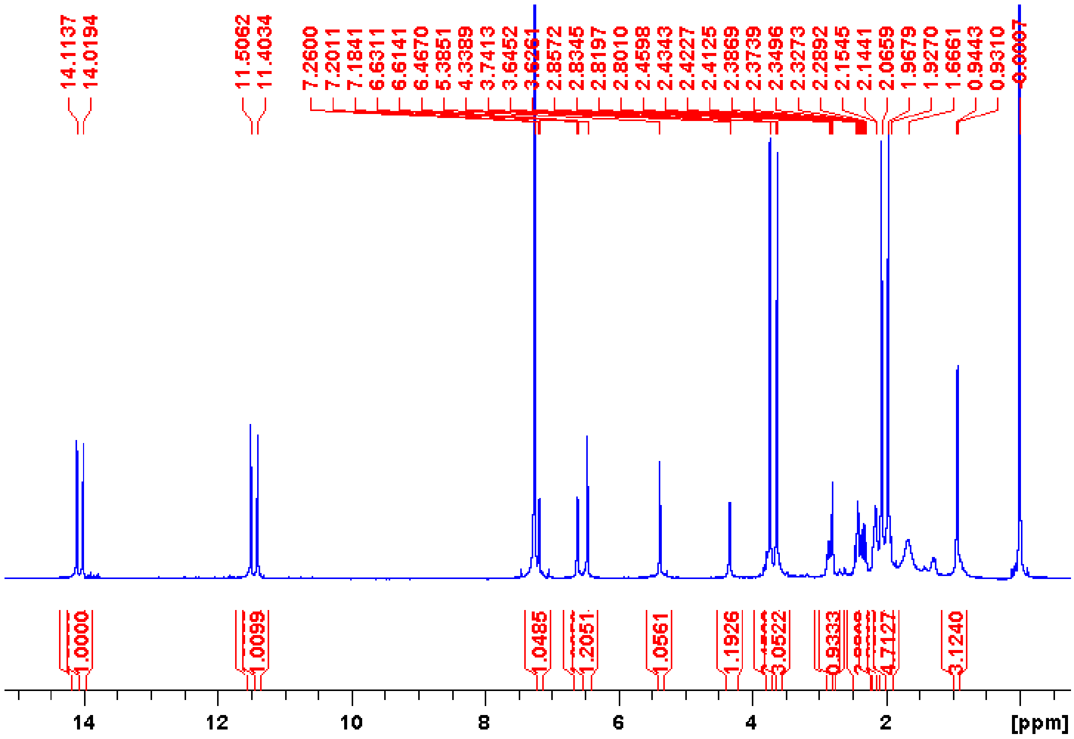

Figure S7. ^{1}H NMR spectrum (500 MHz, $CDCl_3$) of lentulin A (**2**)

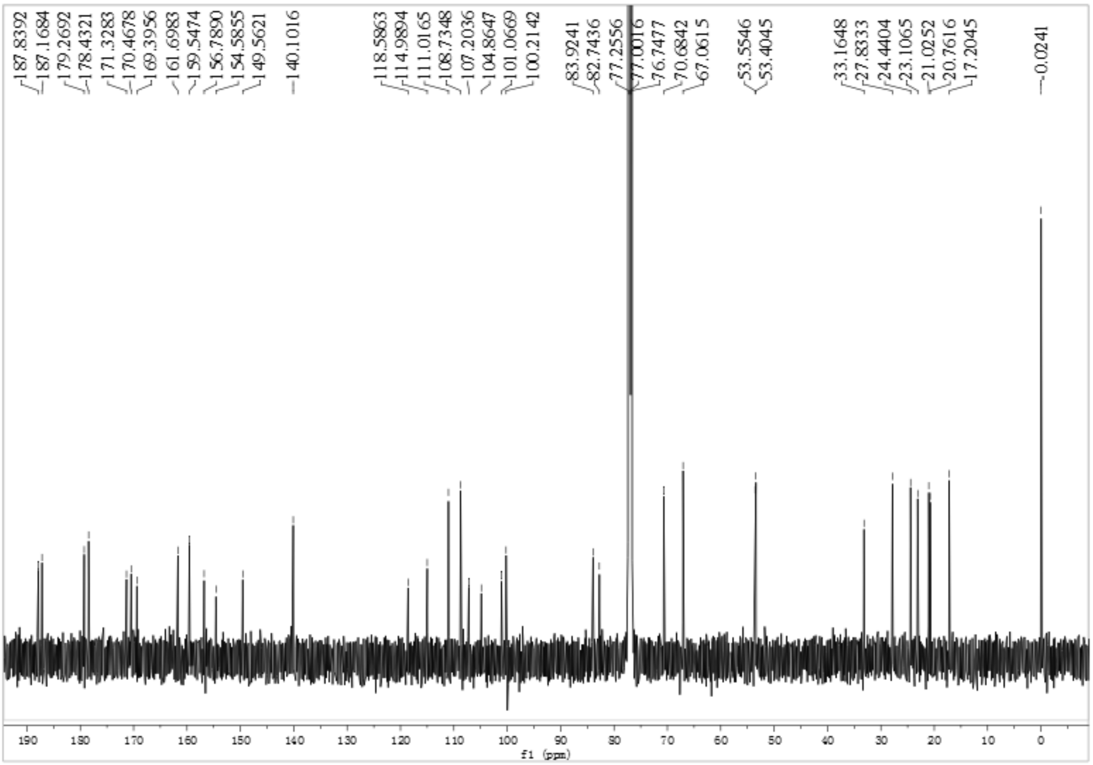

Figure S8. ^{13}C NMR spectrum (125 MHz, $CDCl_3$) of lentulin A (**2**)

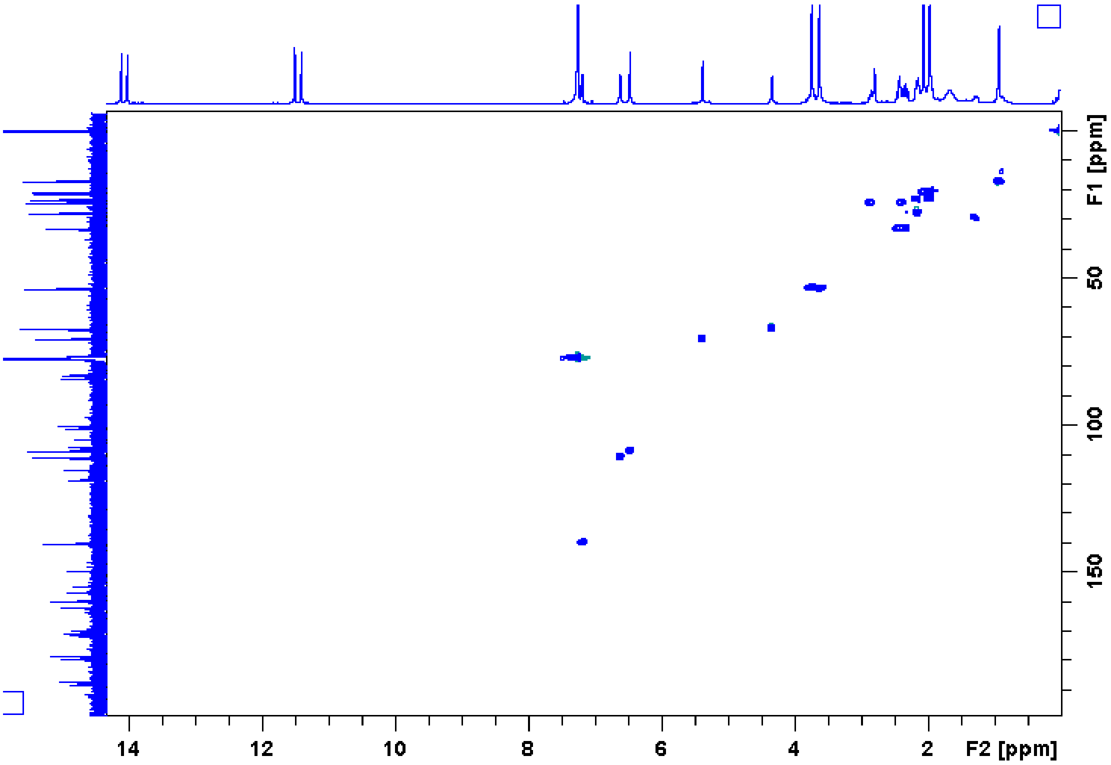

Figure S9. HSQC spectrum ($CDCl_3$) of lentulin A (**2**)

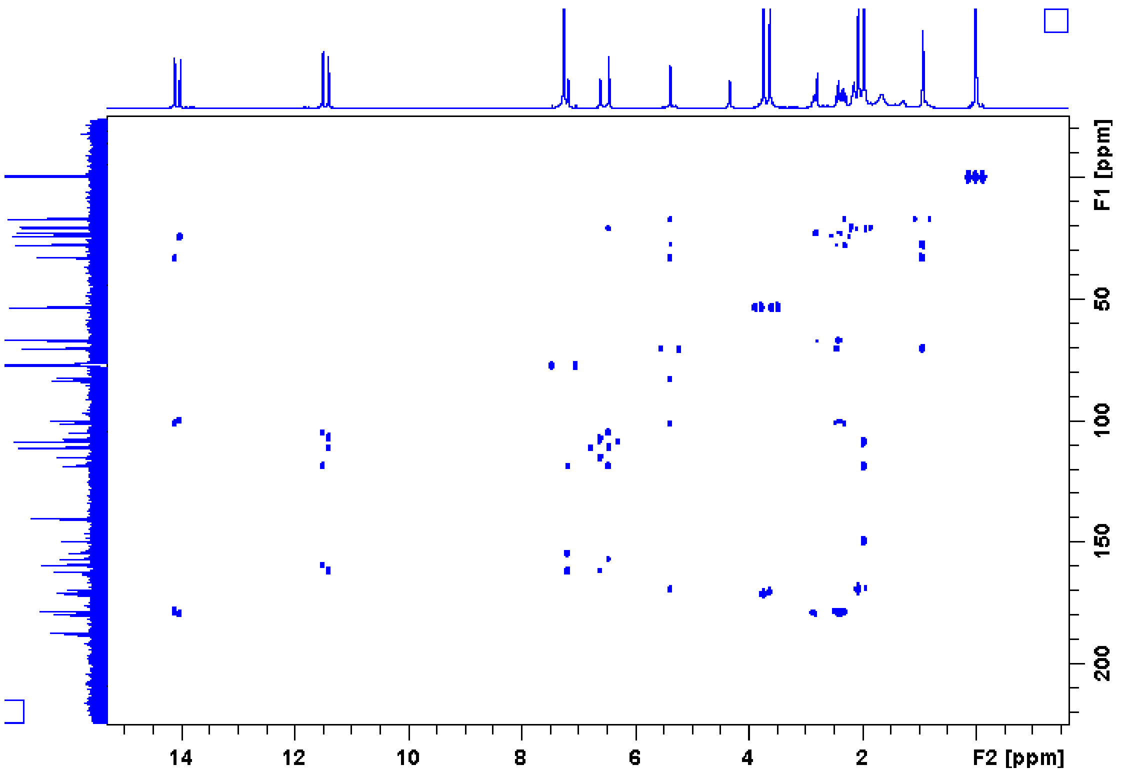

Figure S10. HMBC spectrum ($CDCl_3$) of lentulin A (**2**)

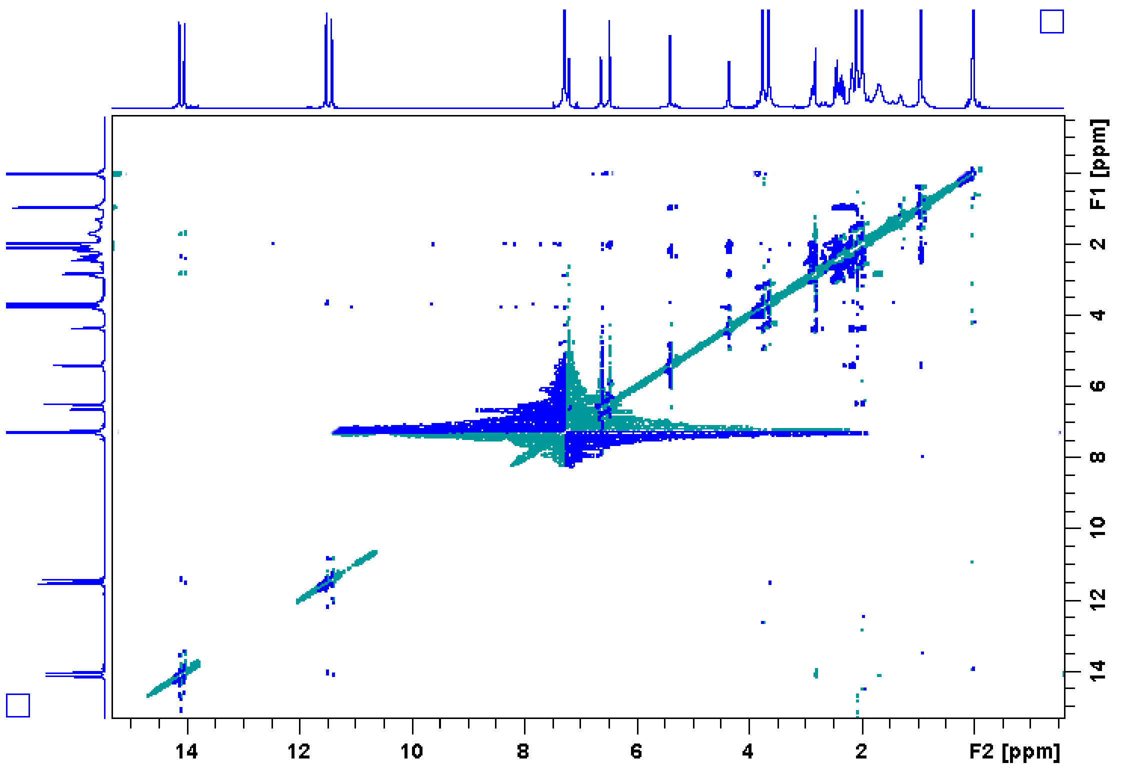

Figure S11. ROESY spectrum ($CDCl_3$) of lentulin A (**2**)

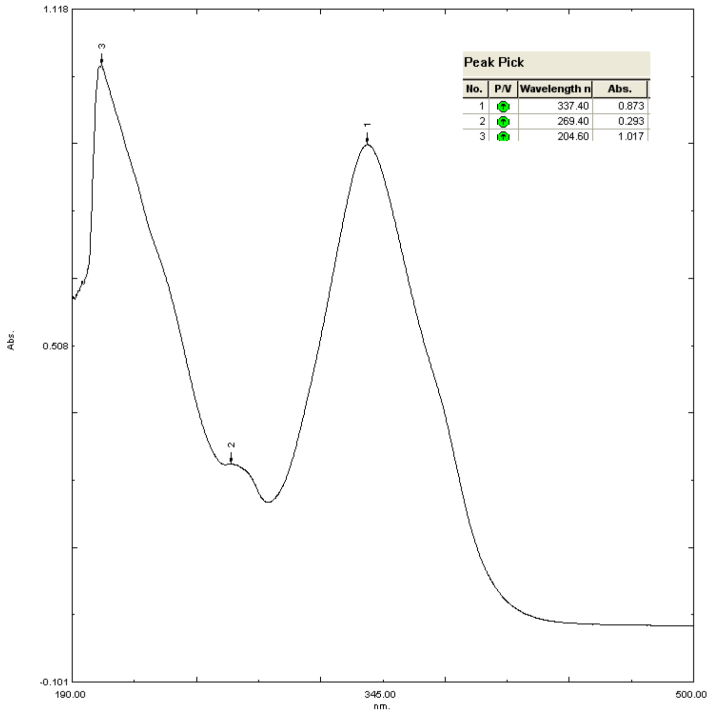

Figure S12. UV spectrum (MeOH) of lentulin A (**2**)

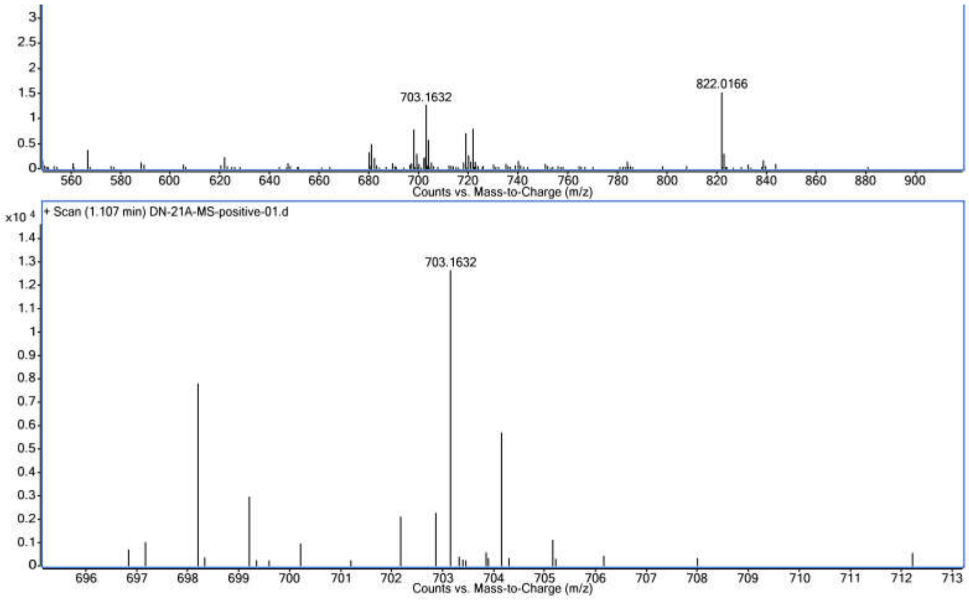

Elemental Composition Calculator

**Target m/z:**	703.1632	**Result type:**	Positive ions	**Species:**	$[M+Na]^+$
**Elements:**		C (0-80); H (0-120); O (0-30); N(0-10); Na (0-5)			
**Ion Formula**		**Calcalated m/z**		**PPM Error**	
C34H32NaO15		703.1633		0.14	

Figure S13. HRESIMS spectrum of lentulin B (**3**)

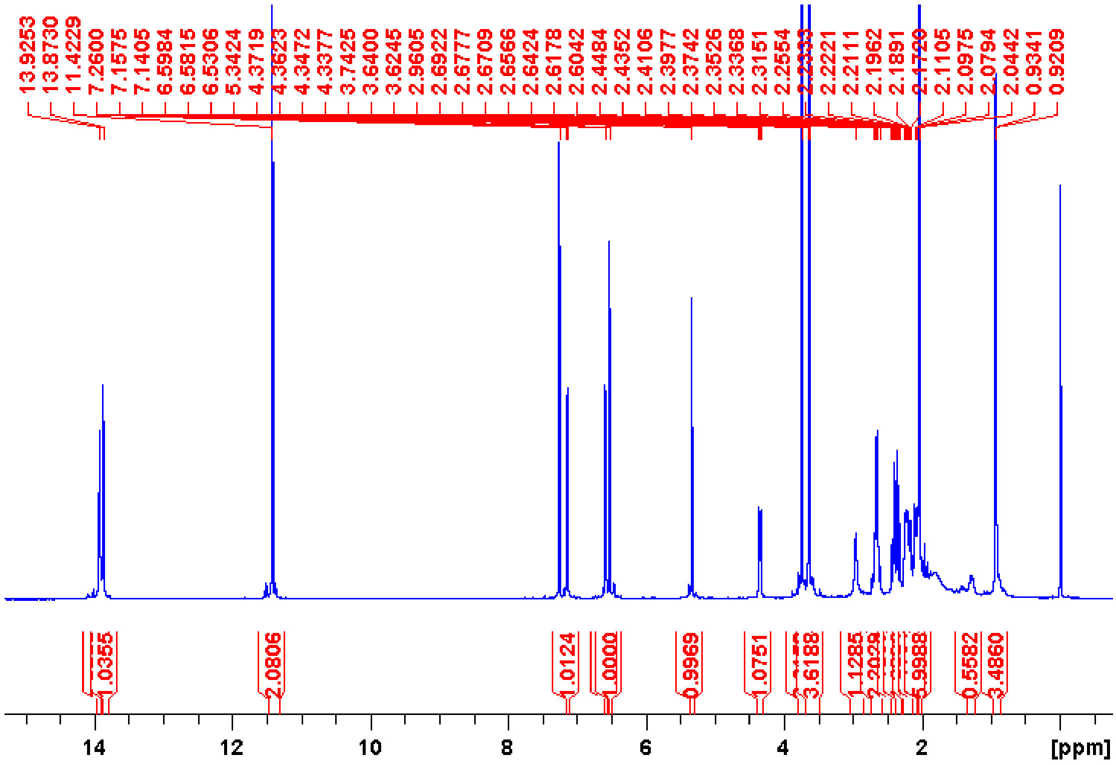

Figure S14. ^{1}H NMR spectrum (500 MHz, $CDCl_3$) of lentulin B (**3**)

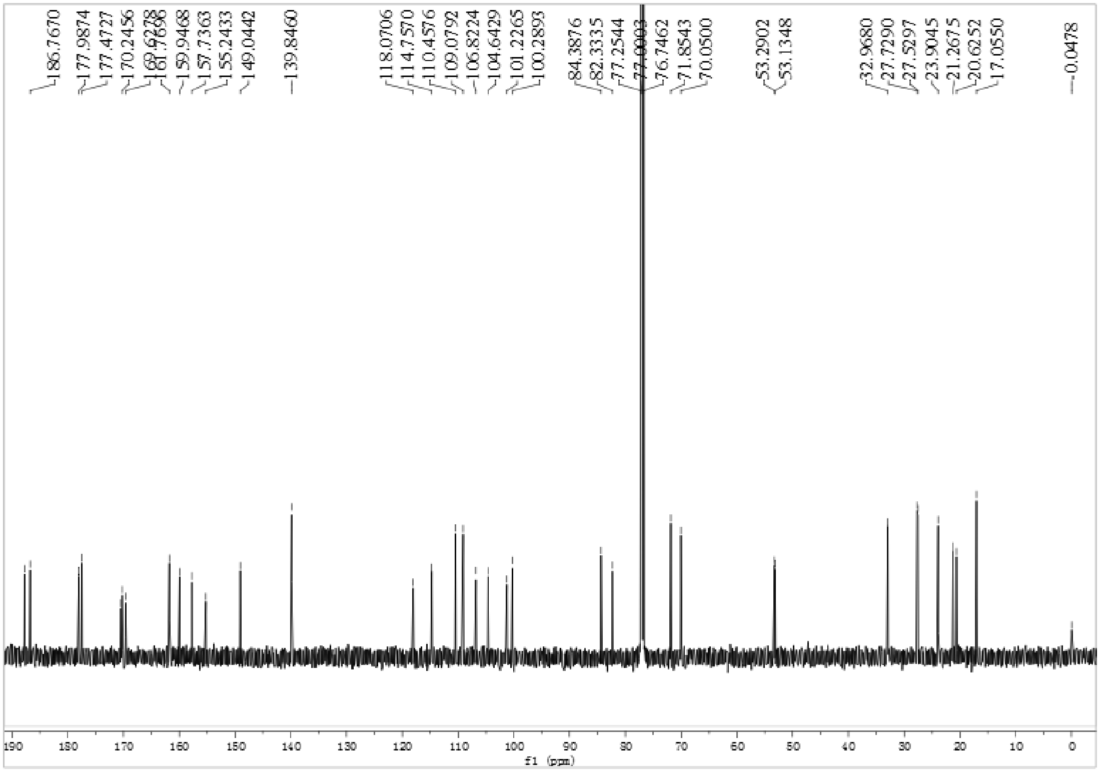

Figure S15. ^{13}C NMR spectrum (125 MHz, $CDCl_3$) of lentulin B (**3**)

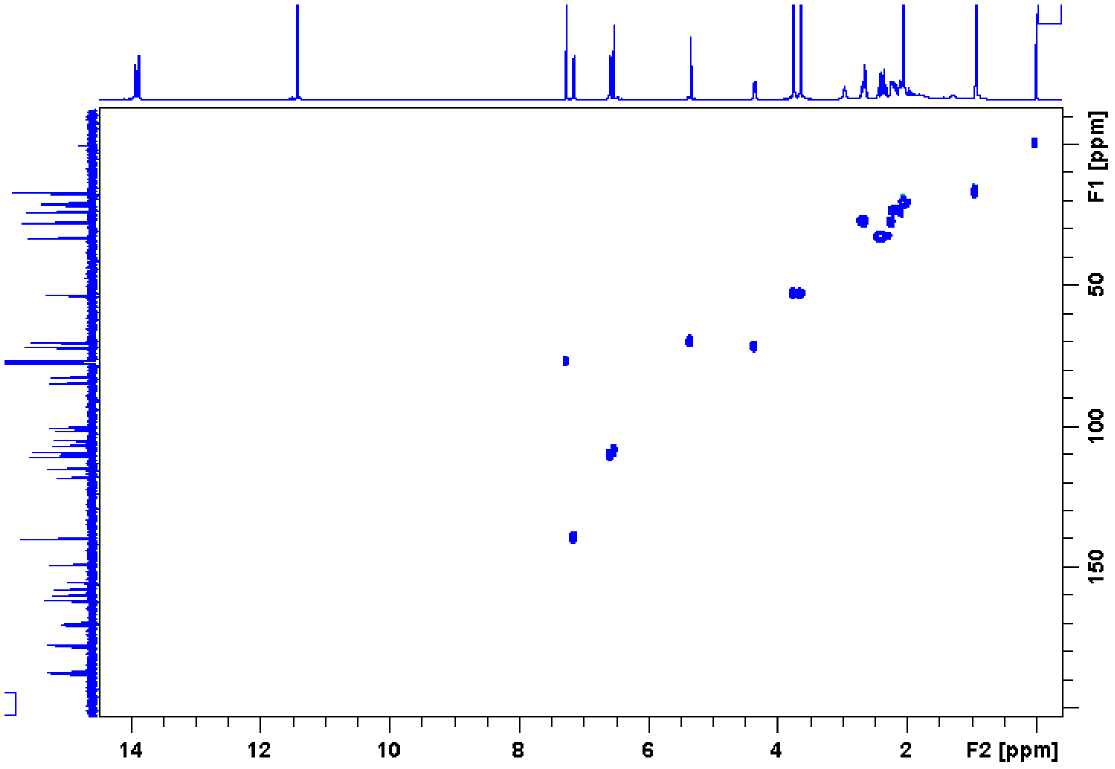

Figure S16. HSQC spectrum ($CDCl_3$) of lentulin B (**3**)

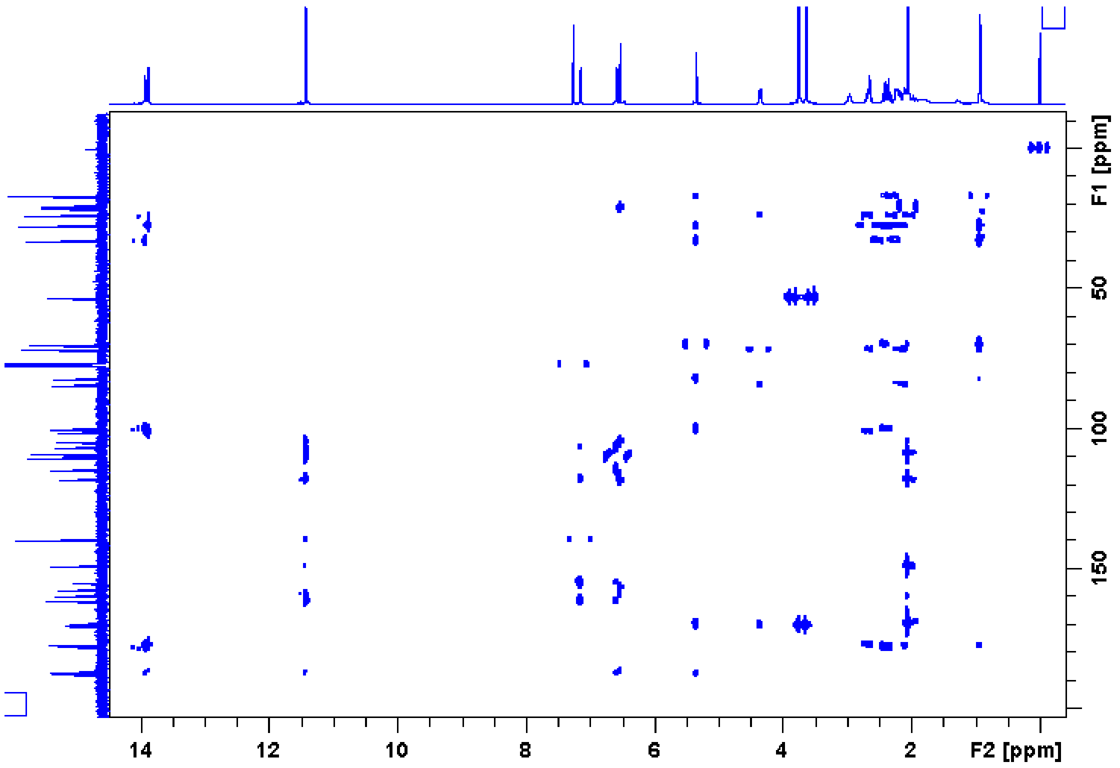

Figure S17. HMBC spectrum ($CDCl_3$) of lentulin B (**3**)

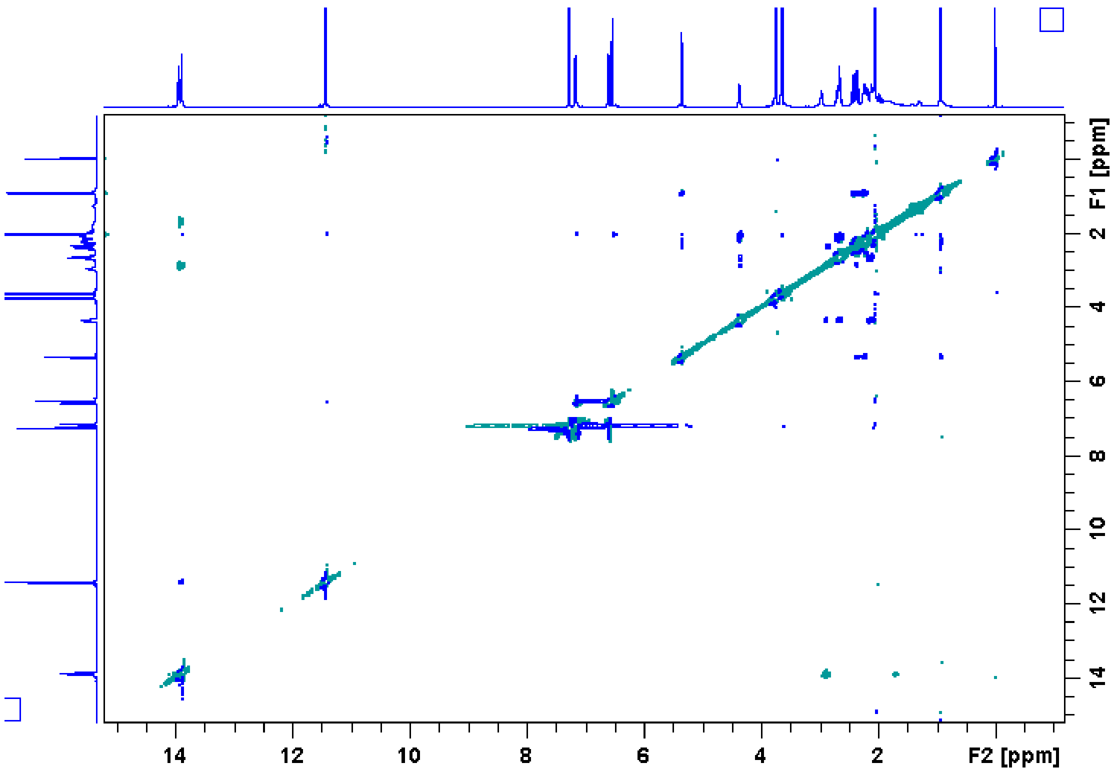

Figure S18. ROESY spectrum ($CDCl_3$) of lentulin B (**3**)

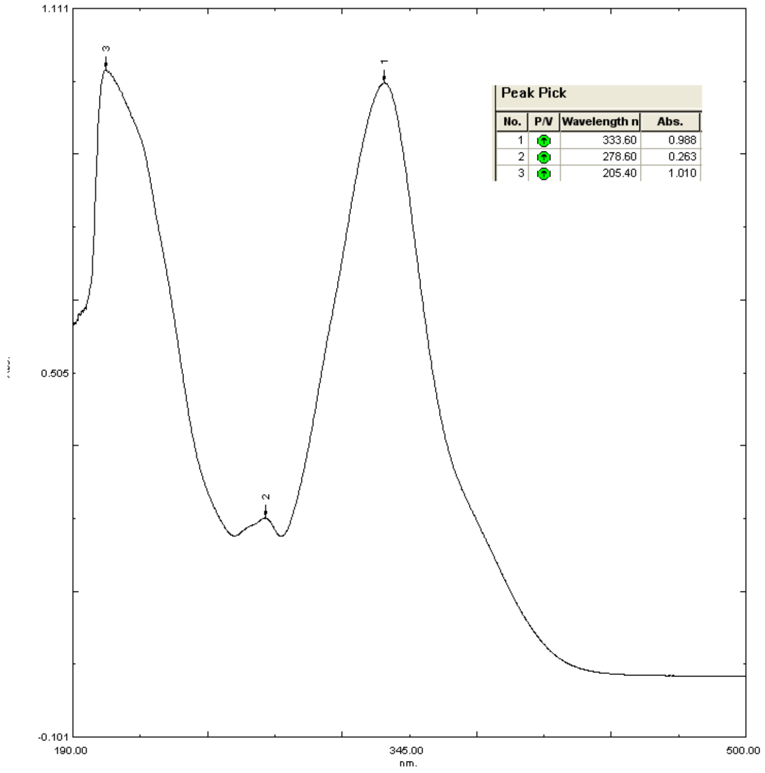

Figure S19. UV spectrum (MeOH) of lentulin B (**3**)

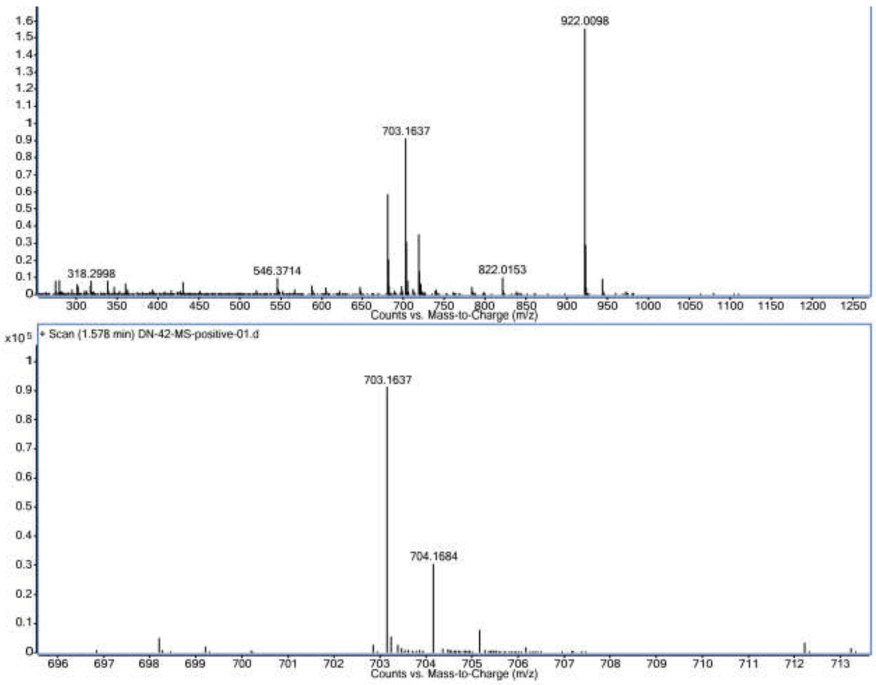

Elemental Composition Calculator

Target m/z:	703.1637	Result type:	Positive ions	Species:	$[M+Na]^+$
Elements:		C (0-80); H (0-120); O (0-30); N(0-10); Na (0-5)			
Ion Formula		Calcalated m/z		PPM Error	
C34H32NaO15		703.1633		-0.44	

Figure S20. HRESIMS spectrum of lentulin C (**4**)

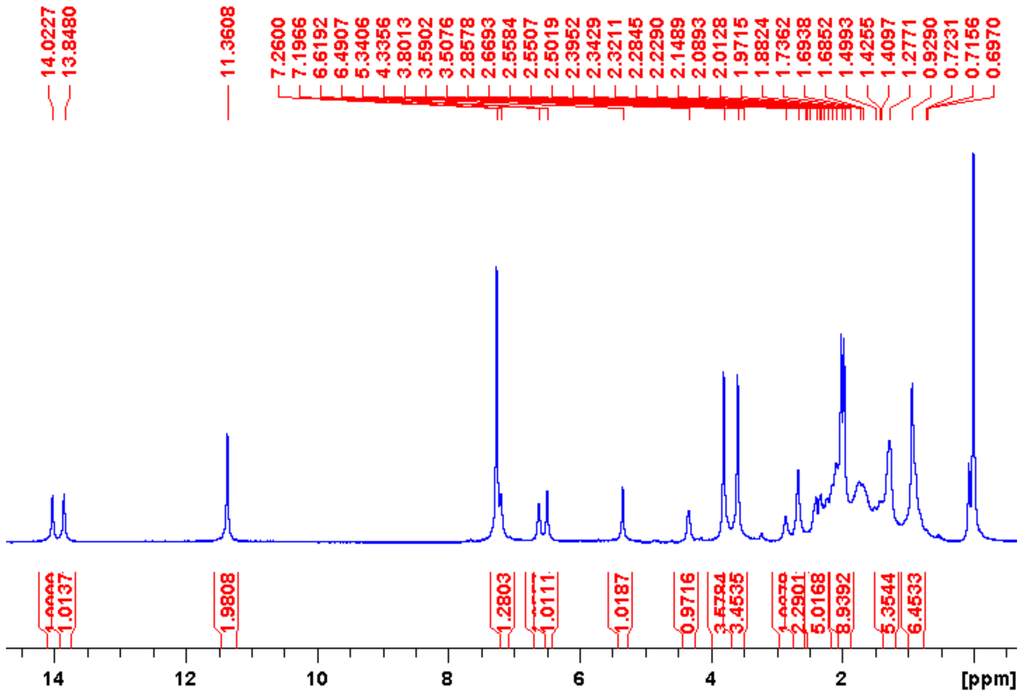

Figure S21. ^{1}H NMR spectrum (500 MHz, $CDCl_3$) of lentulin C (**4**)

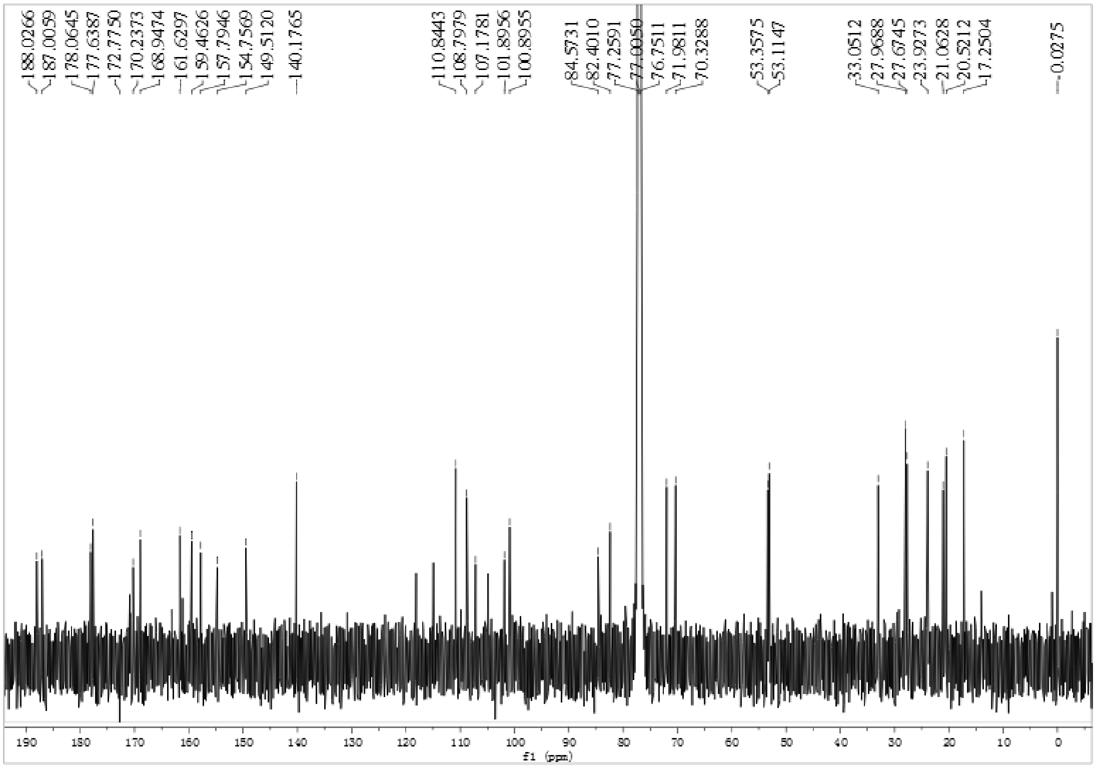

Figure S22. ^{13}C NMR spectrum (125 MHz, $CDCl_3$) of lentulin C (**4**)

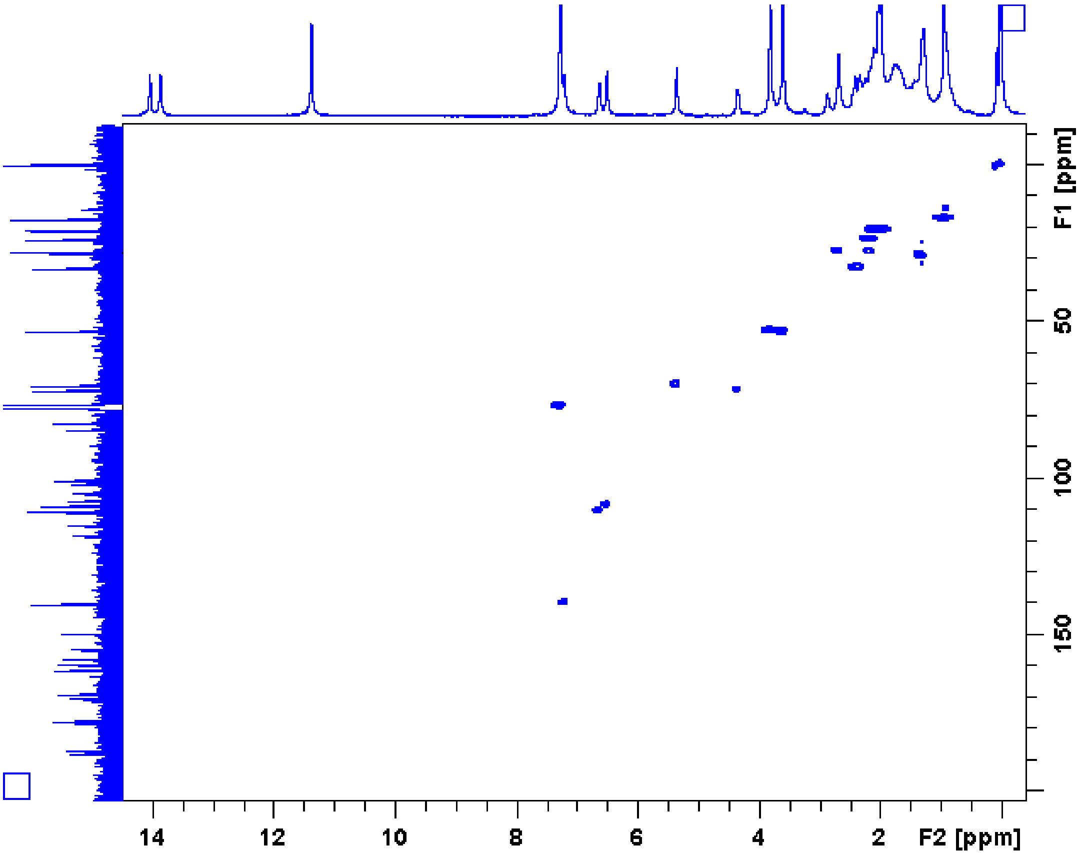

Figure S23. HSQC spectrum ($CDCl_3$) of lentulin C (**4**)

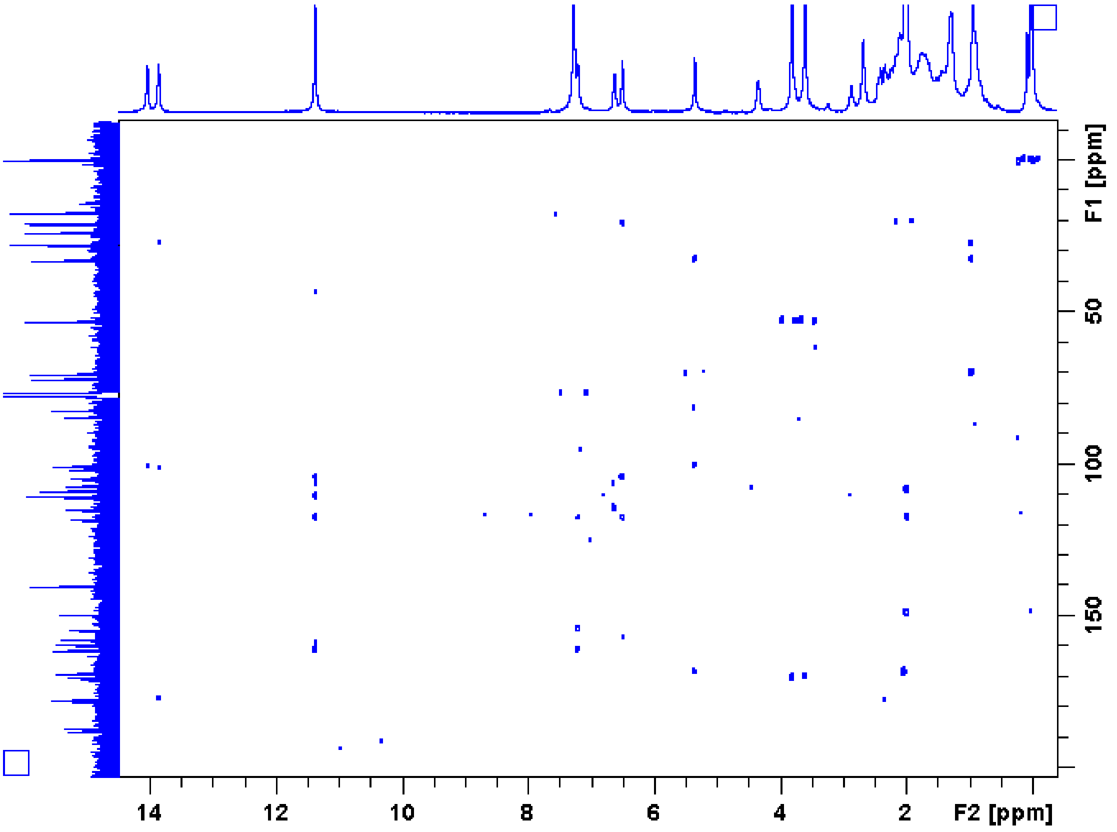

Figure S24. HMBC spectrum ($CDCl_3$) of lentulin C (**4**)

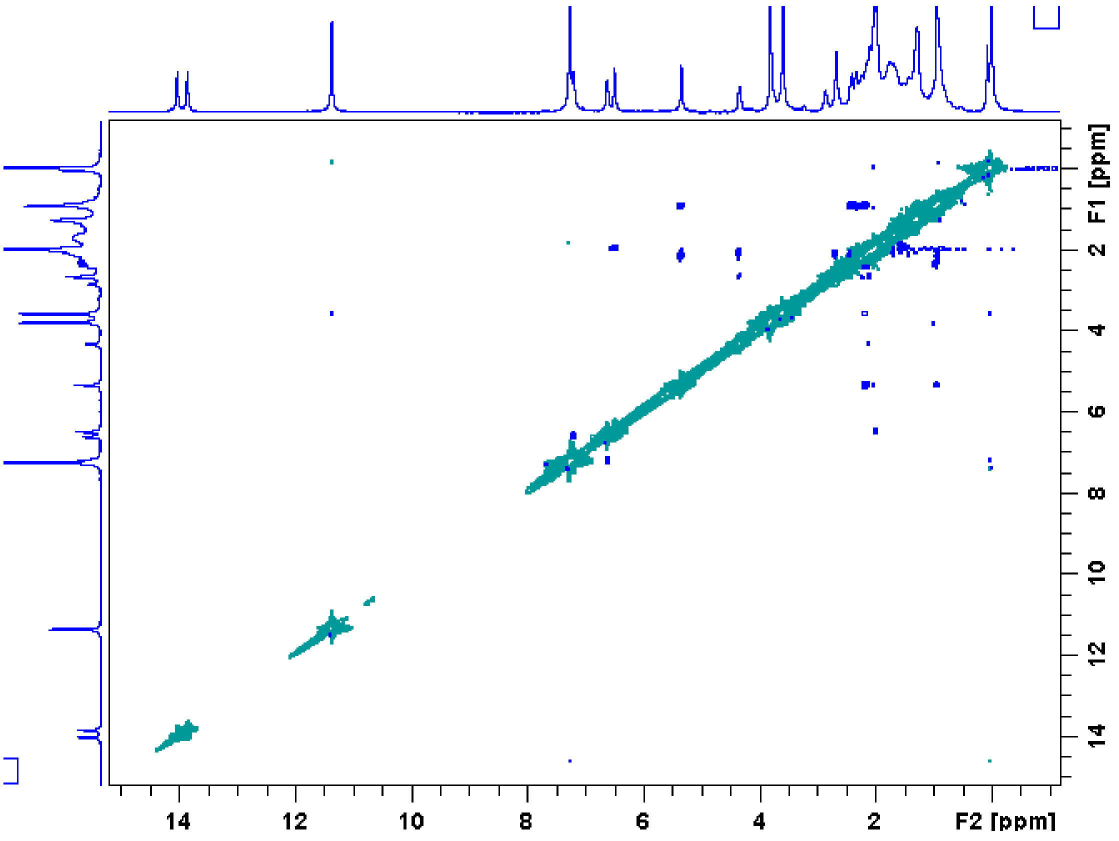

Figure S25. ROESY spectrum ($CDCl_3$) of lentulin C (**4**)

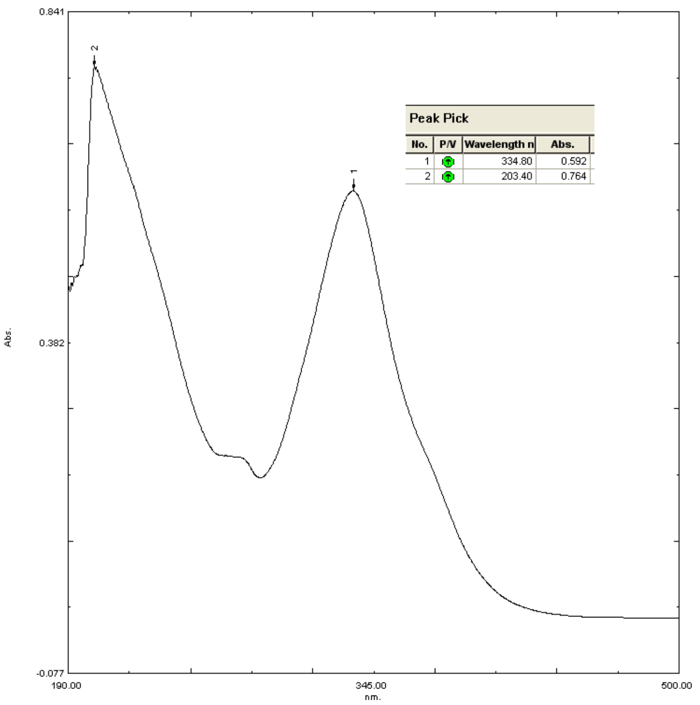

Figure S26. UV spectrum (MeOH) of lentulin C (**4**)

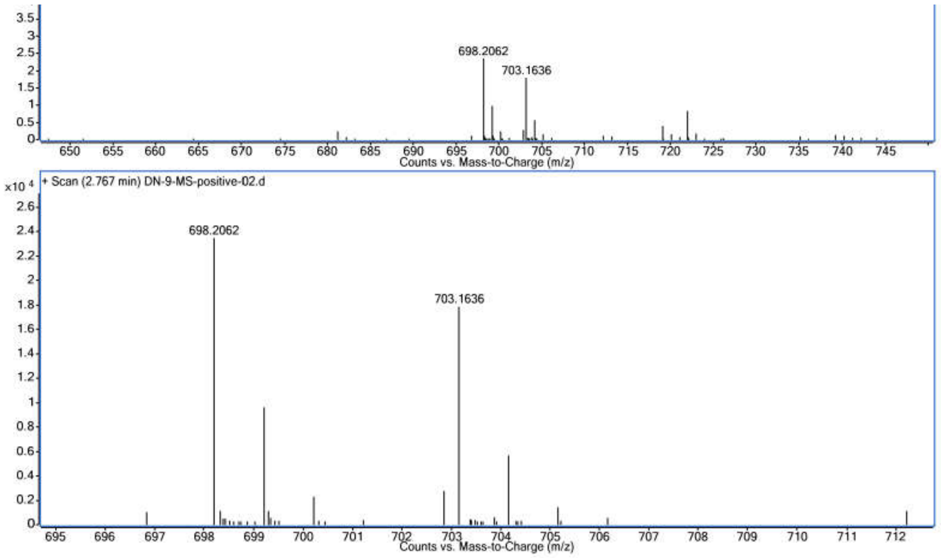

Elemental Composition Calculator

Target m/z:	703.1636	Result type:	Positive ions	Species:	$[M+Na]^+$
Elements:		C (0-80); H (0-120); O (0-30); N(0-10); Na (0-5)			
Ion Formula		Calcalated m/z		PPM Error	
C34H32NaO15		703.1633		-0.38	

Figure S27. HRESIMS spectrum of lentulin D (**5**)

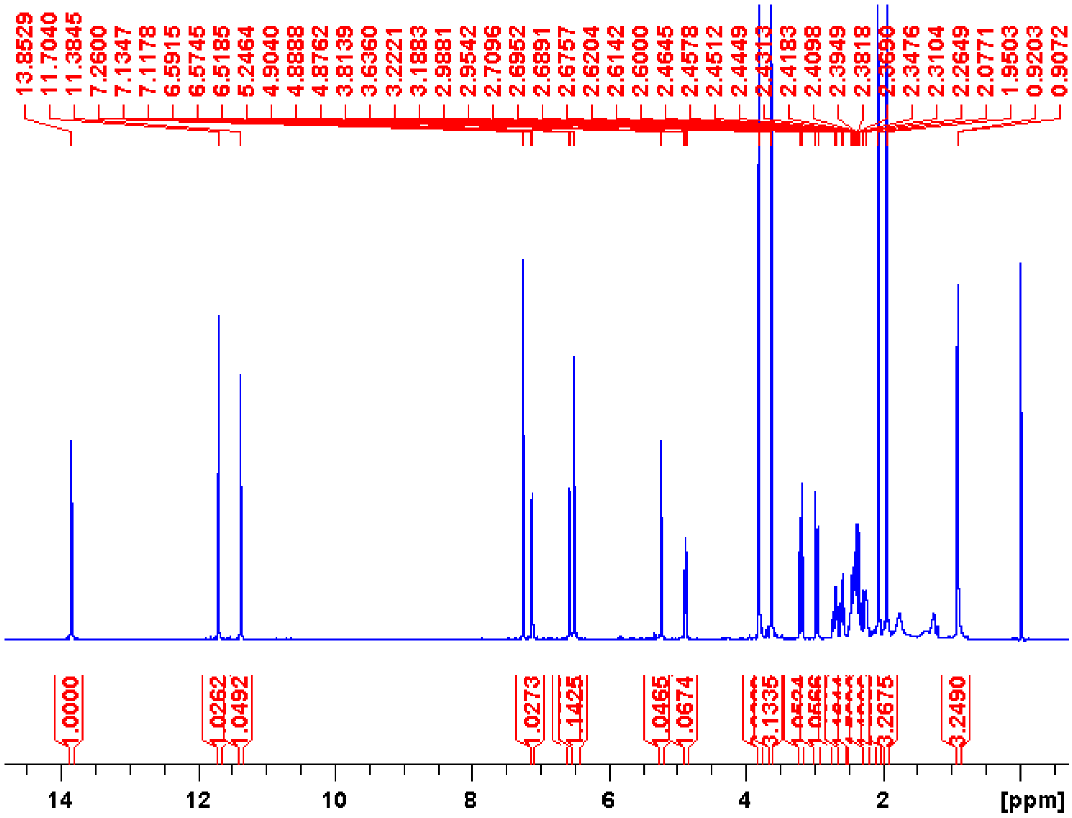

Figure S28. ^{1}H NMR spectrum (500 MHz, $CDCl_3$) of lentulin D (**5**)

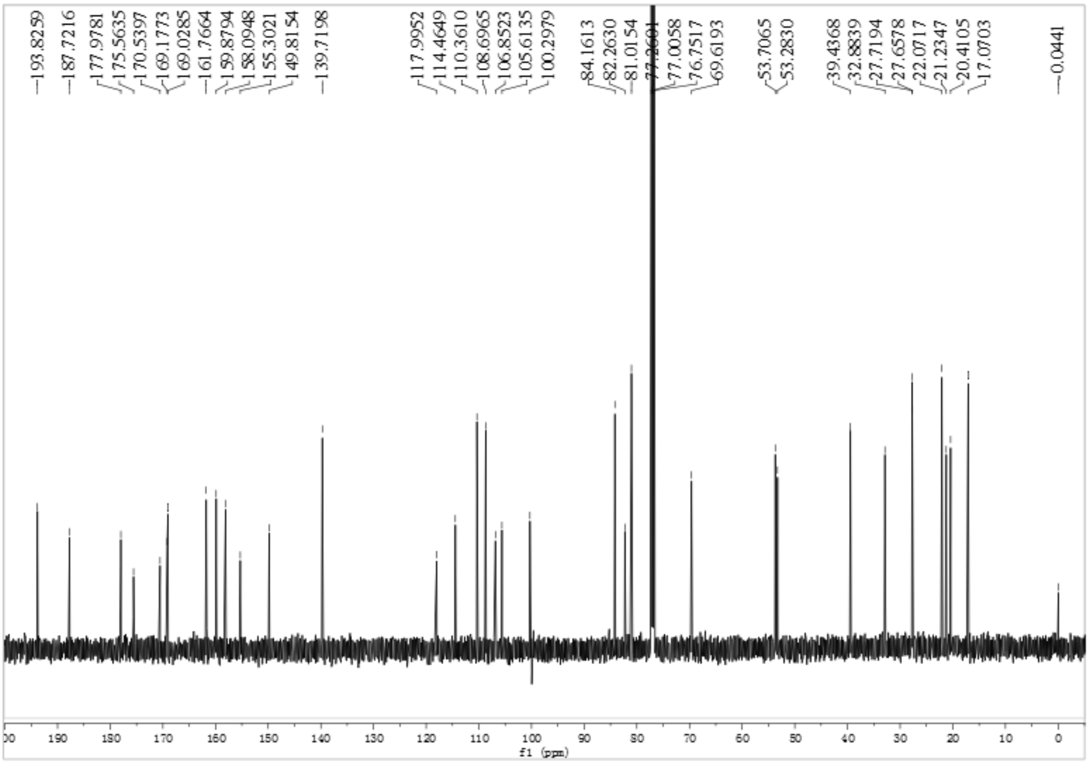

Figure S29. ^{13}C NMR spectrum (125 MHz, $CDCl_3$) of lentulin D (**5**)

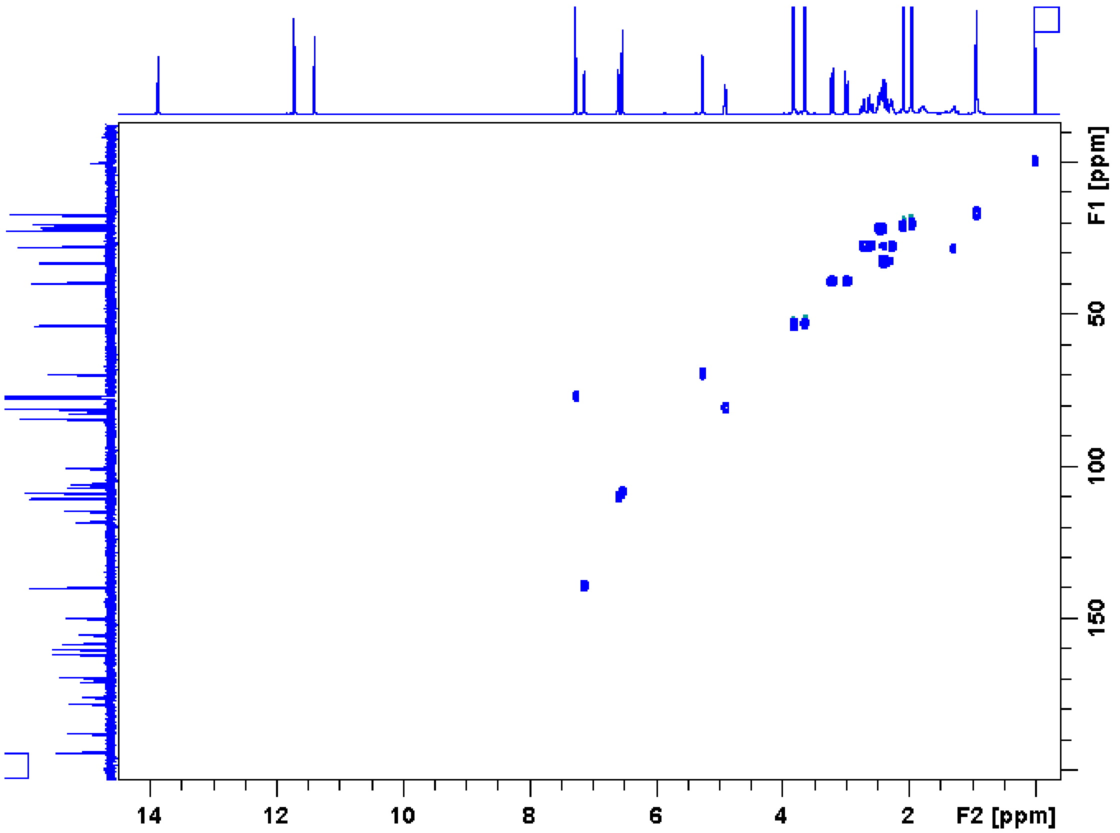

Figure S30. HSQC spectrum ($CDCl_3$) of lentulin D (**5**)

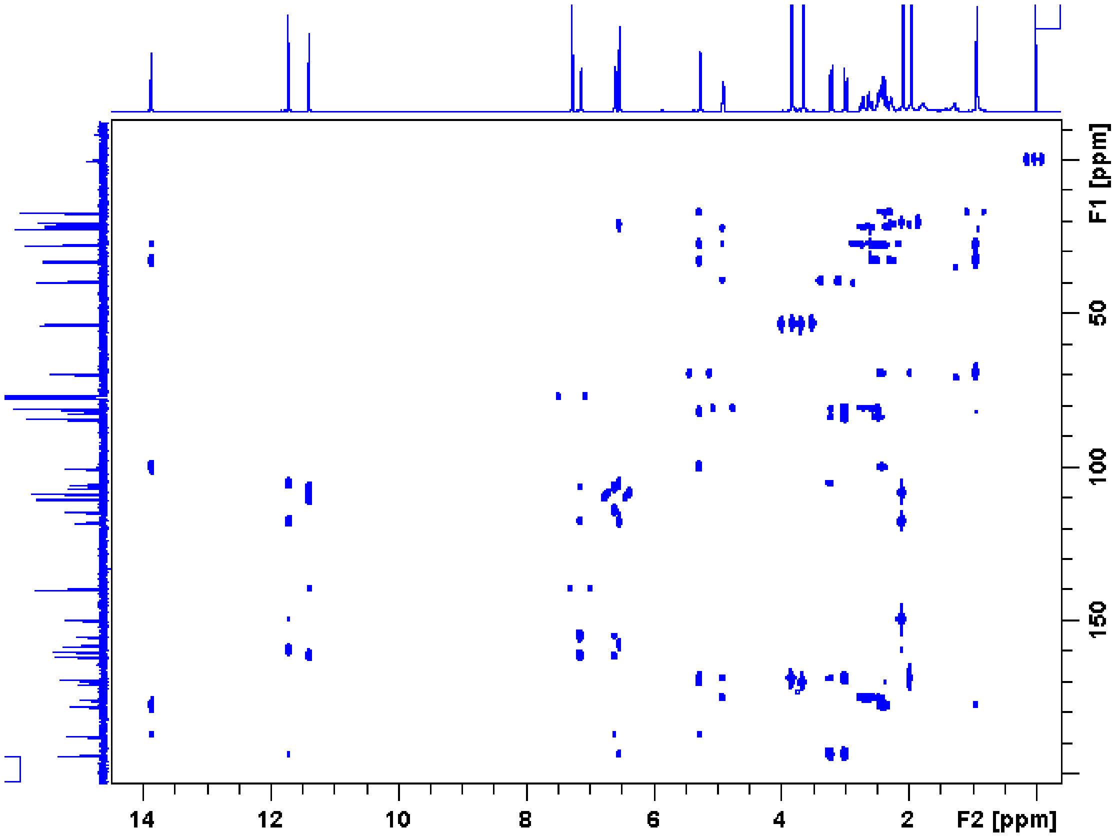

Figure S31. HMBC spectrum ($CDCl_3$) of lentulin D (**5**)

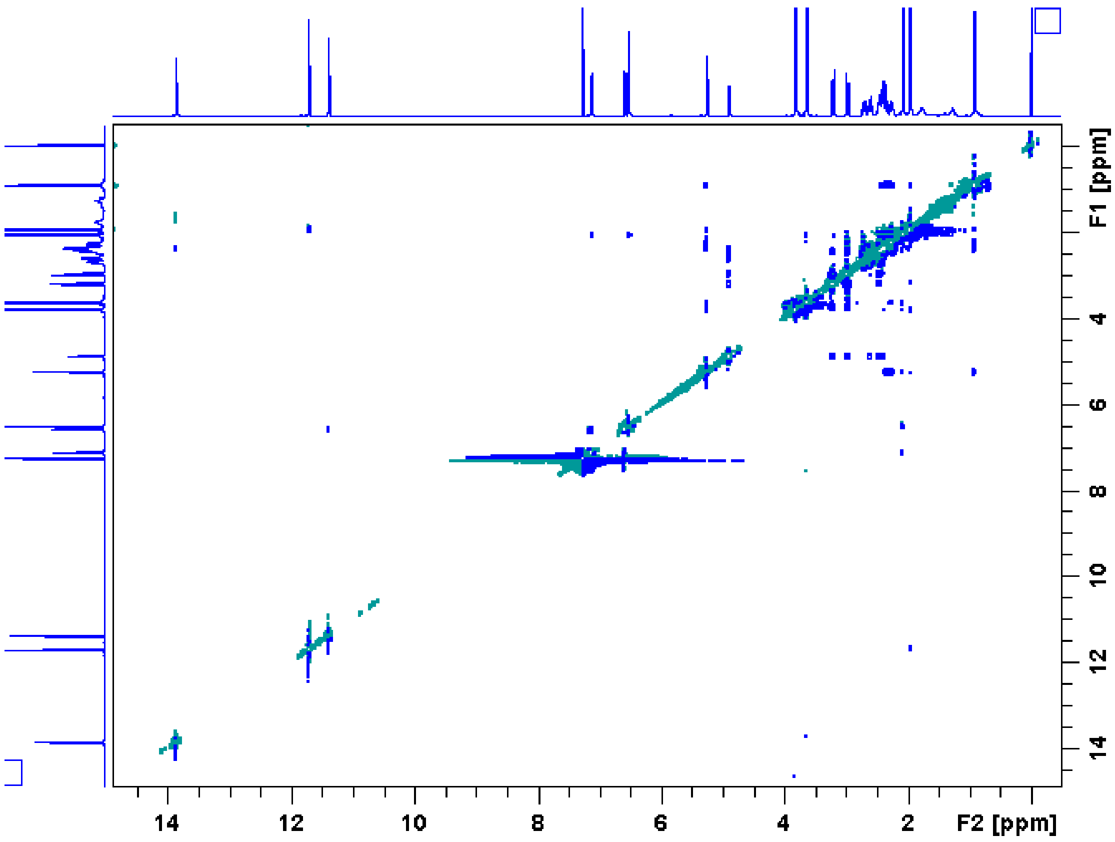

Figure S32. ROESY spectrum ($CDCl_3$) of lentulin D (**5**)

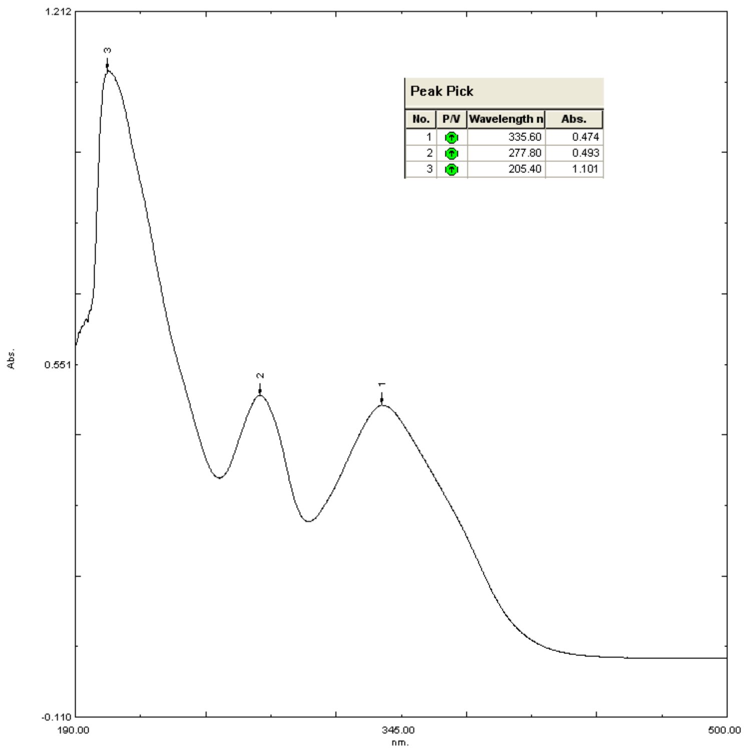

Figure S33. UV spectrum (MeOH) of lentulin D (**5**)

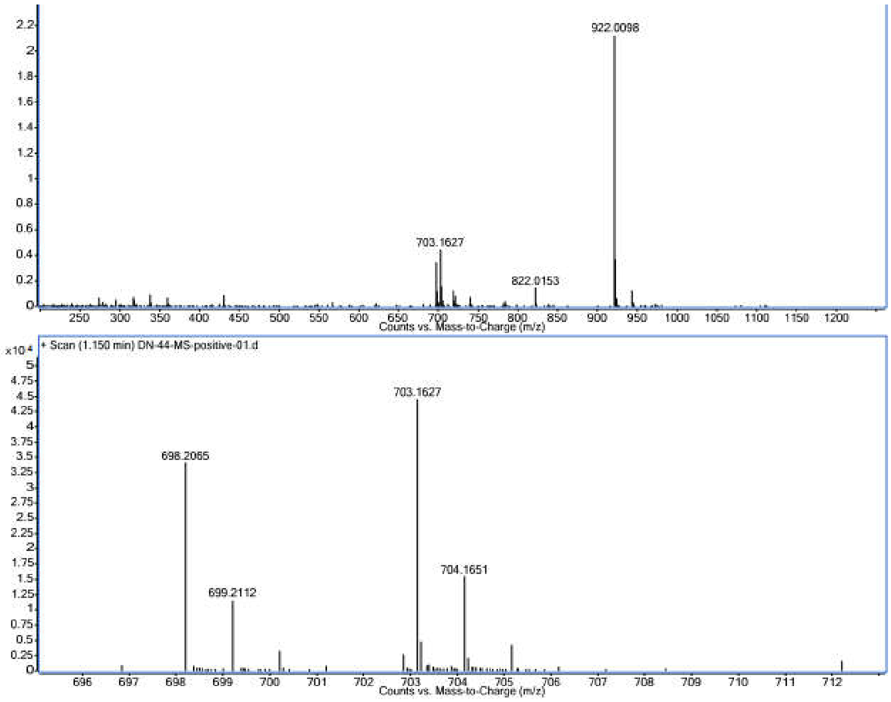

Elemental Composition Calculator

Target m/z:	703.1627	Result type:	Positive ions	Species:	$[M+Na]^+$
Elements:		C (0-80); H (0-120); O (0-30); N(0-10); Na (0-5)			
Ion Formula		Calcalated m/z		PPM Error	
C34H32NaO15		703.1633		0.97	

Figure S34. HRESIMS spectrum of lentulin E (**6**)

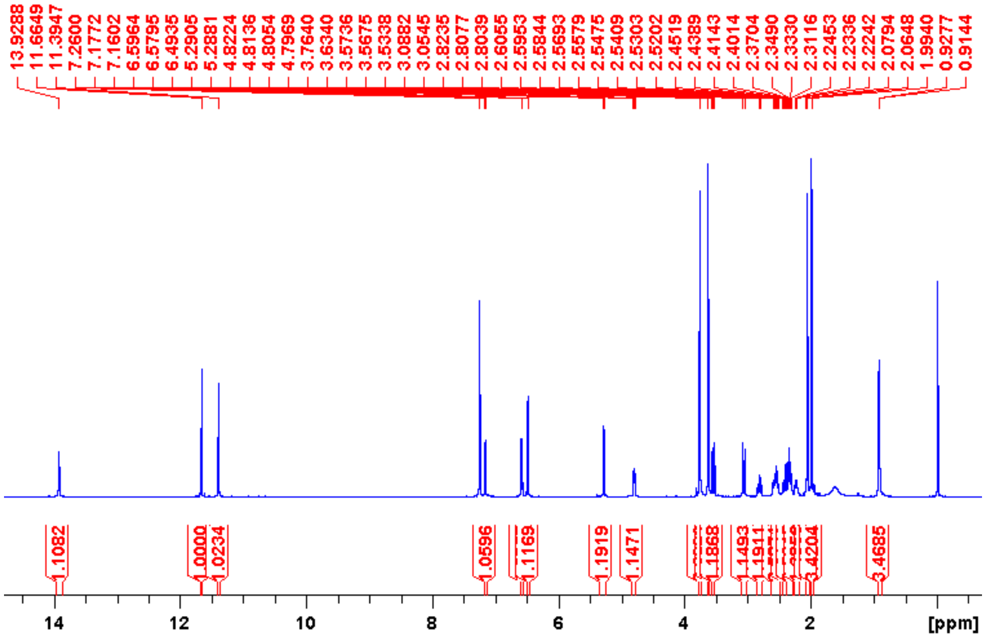

Figure S35. ^{1}H NMR spectrum (500 MHz, $CDCl_3$) of lentulin E (**6**)

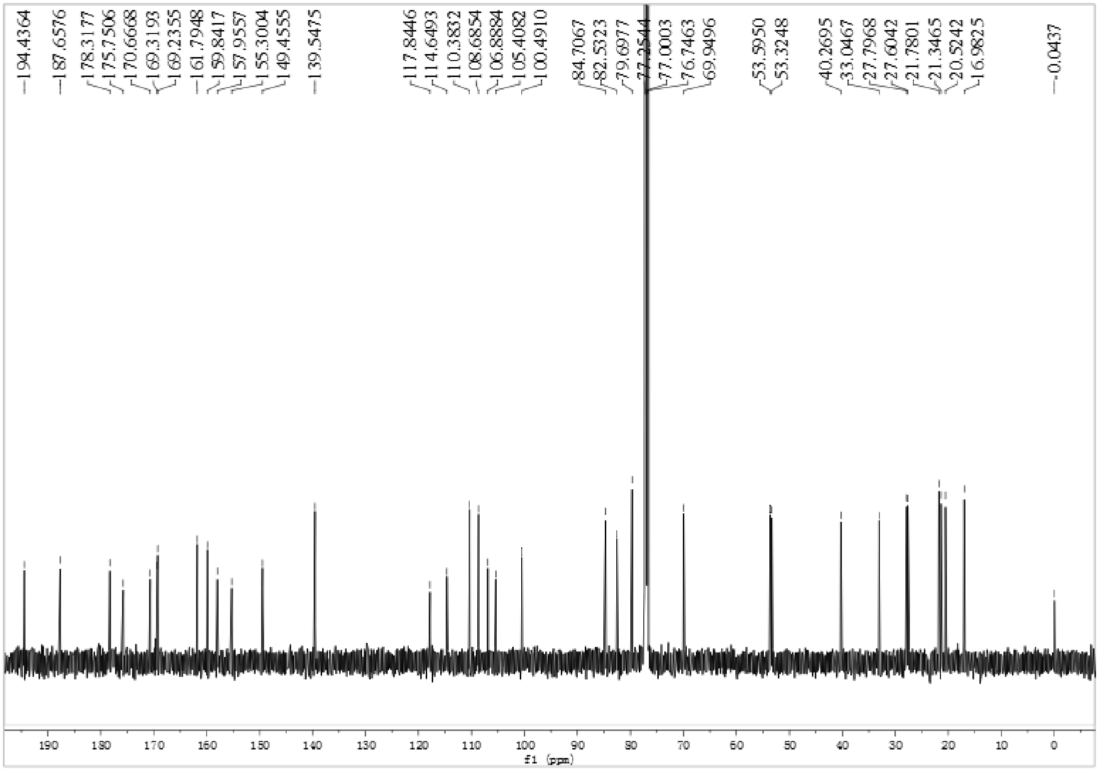

Figure S36. ^{13}C NMR spectrum (125 MHz, $CDCl_3$) of lentulin E (**6**)

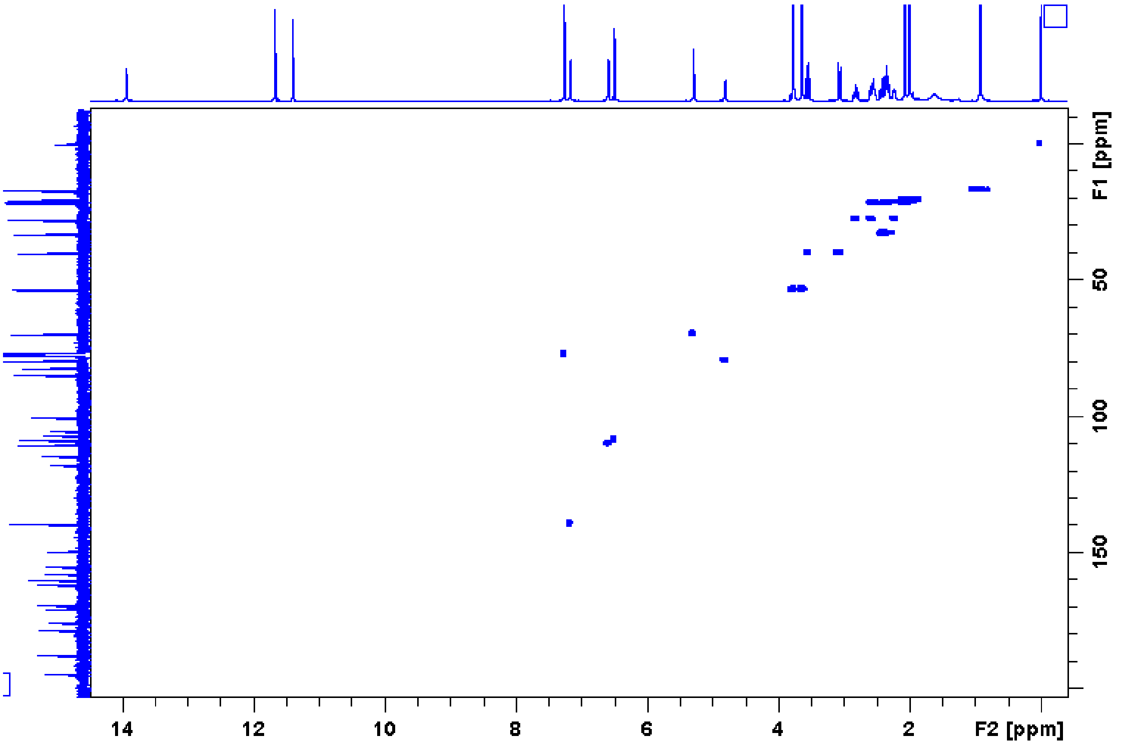

Figure S37. HSQC spectrum ($CDCl_3$) of lentulin E (**6**)

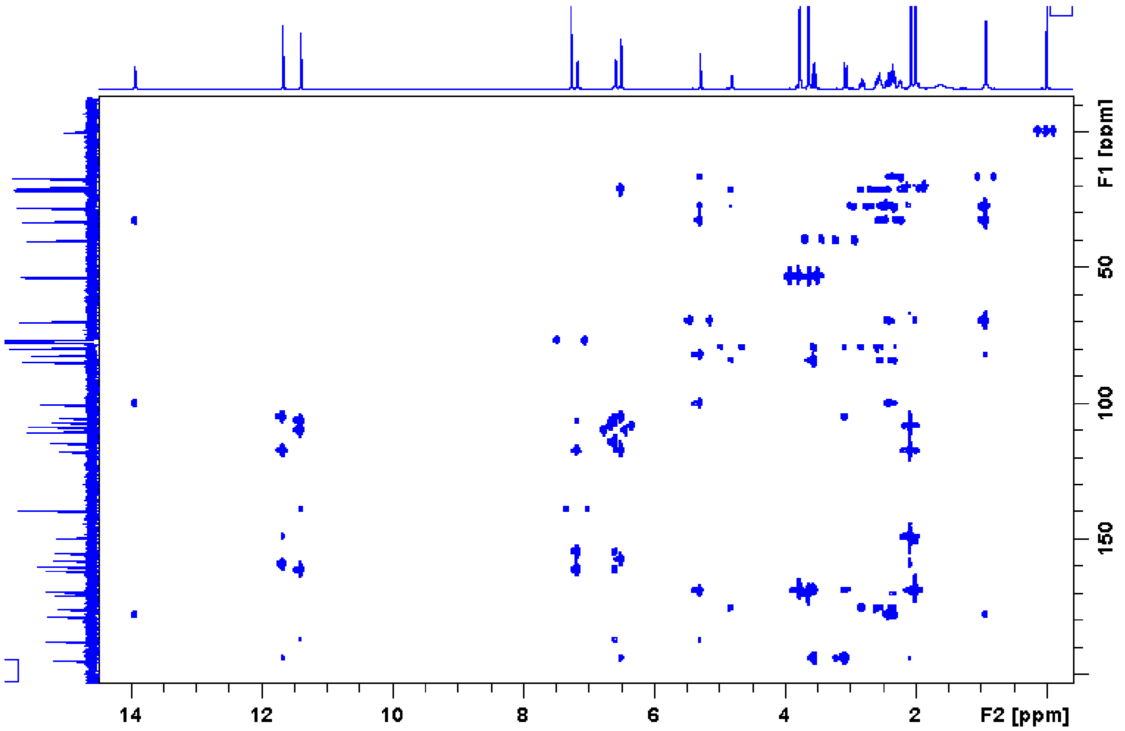

Figure S38. HMBC spectrum ($CDCl_3$) of lentulin E (**6**)

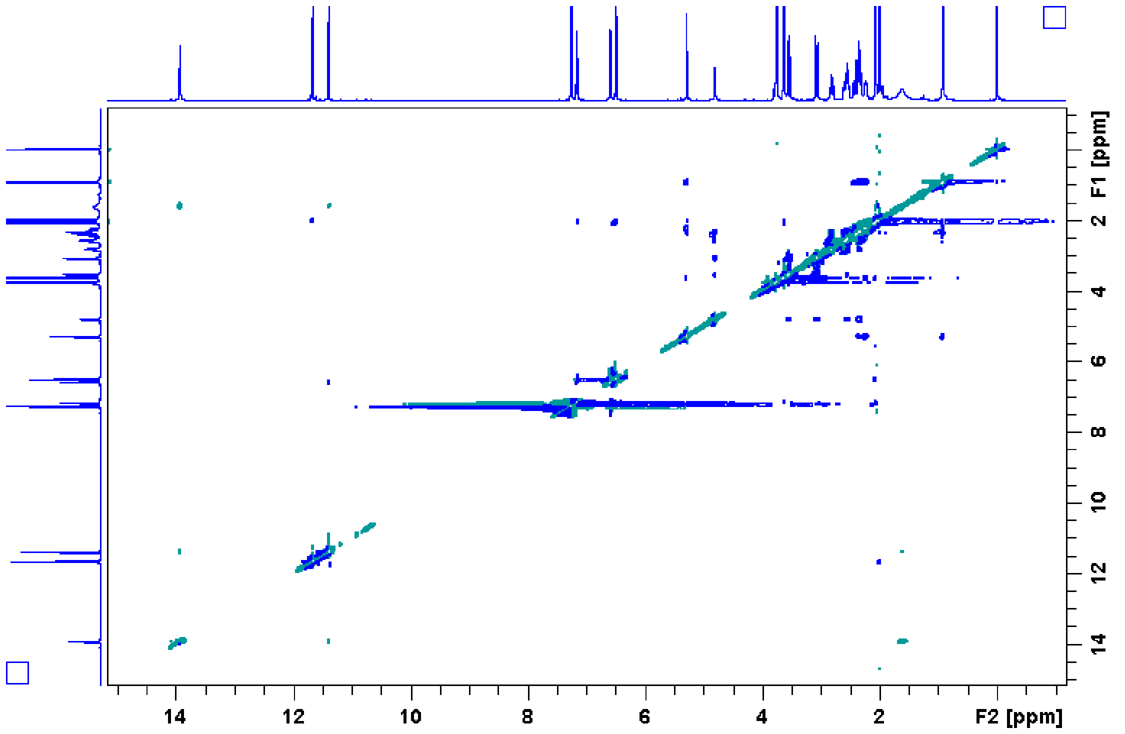

Figure S39. ROESY spectrum ($CDCl_3$) of lentulin E (**6**)

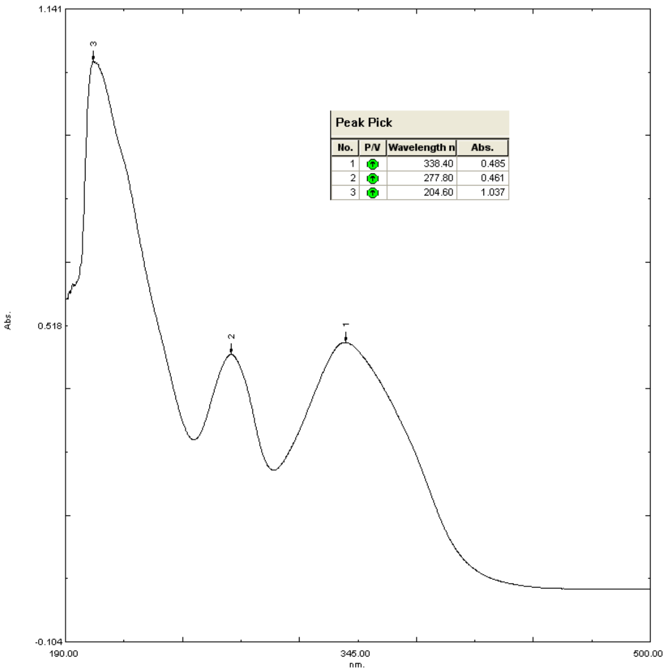

Figure S40. UV spectrum (MeOH) of lentulin E (**6**)

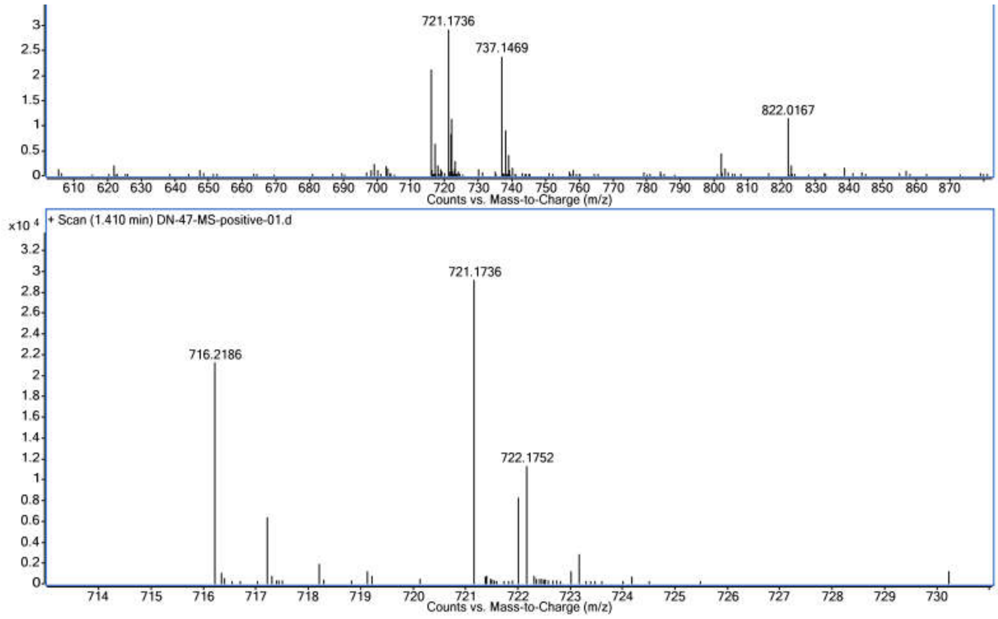

Elemental Composition Calculator

**Target m/z:**	721.1736	**Result type:**	Positive ions	**Species:**	$[M+Na]^+$
**Elements:**		C (0-80); H (0-120); O (0-30); N(0-10); Na (0-5)			
**Ion Formula**		**Calcalated m/z**		**PPM Error**	
C34H34NaO16		721.1739		0.45	

Figure S41. HRESIMS spectrum of lentulin F (**7**)

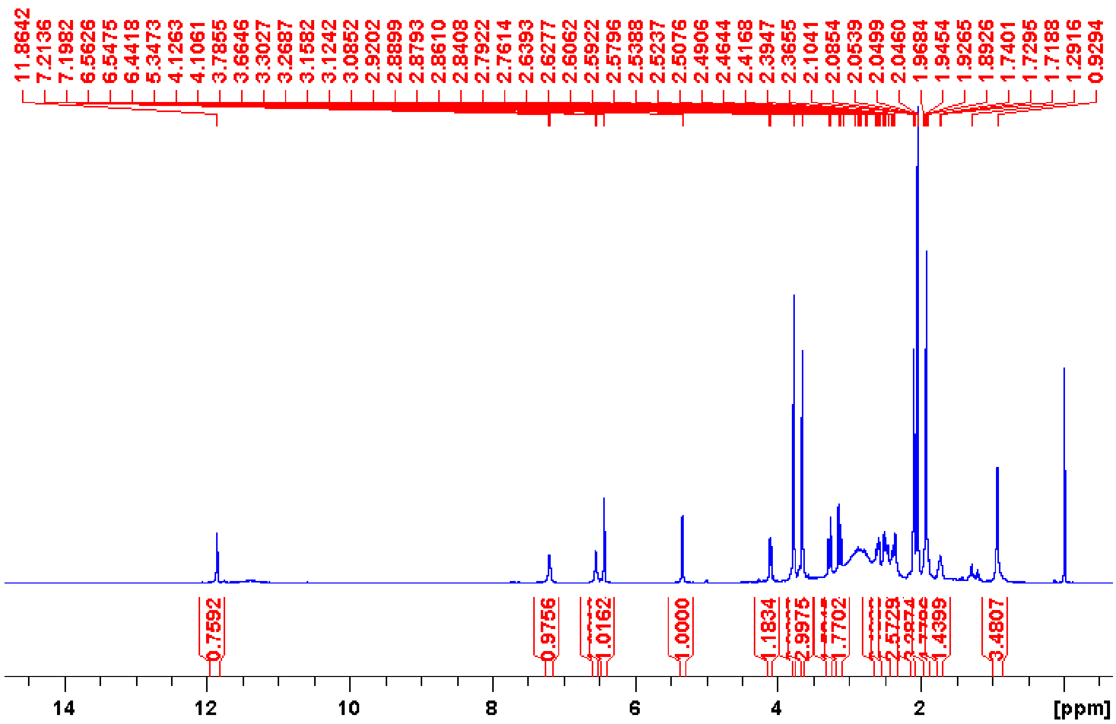

Figure S42. ^{1}H NMR spectrum (500 MHz, Acetone-d_6) of lentulin F (**7**)

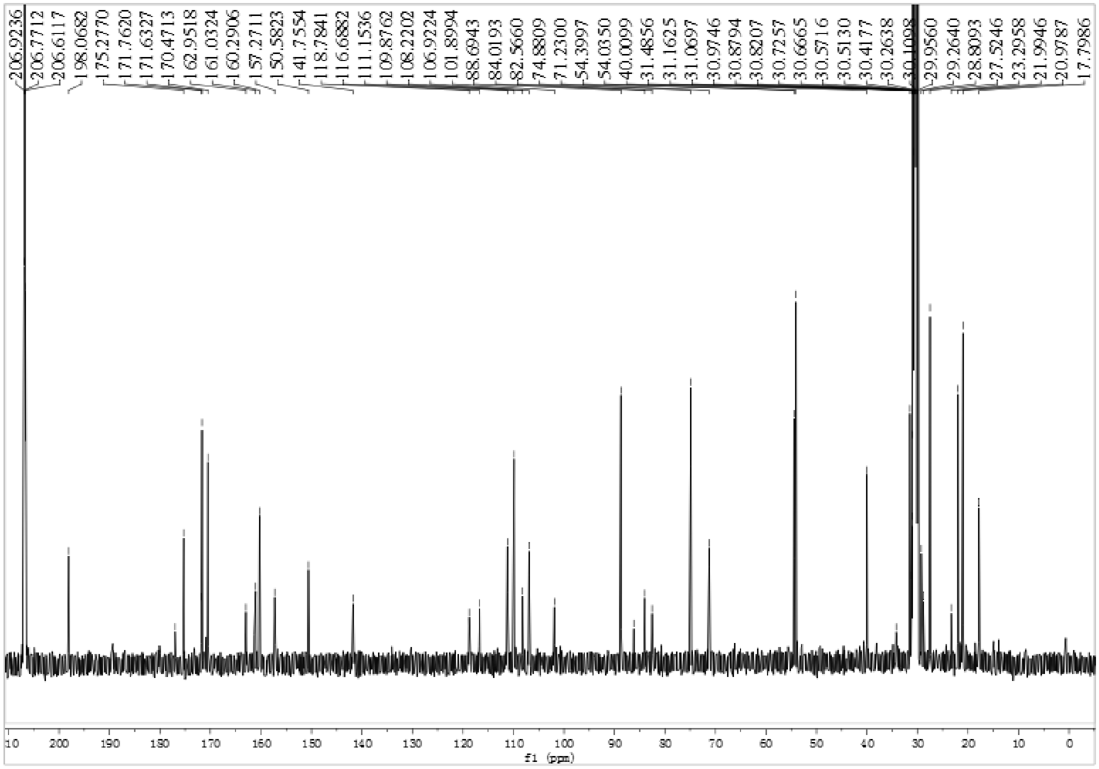

Figure S43. ^{13}C NMR spectrum (125 MHz, Acetone-d_6) of lentulin F (**7**)

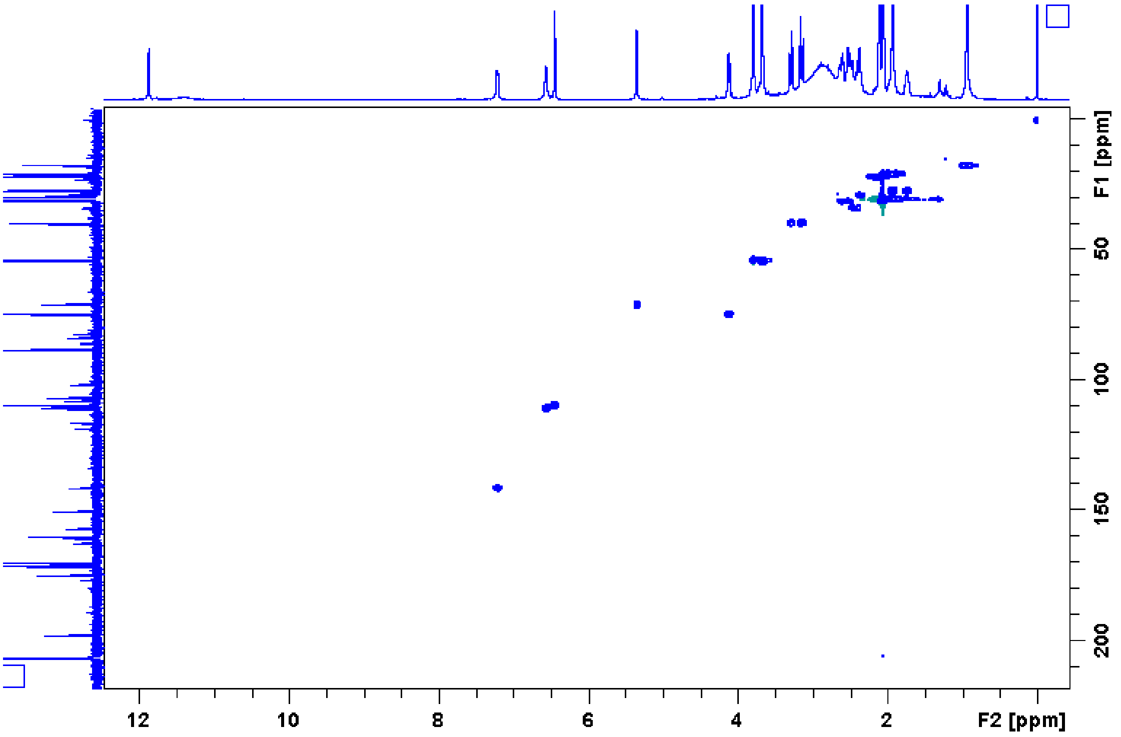

Figure S44. HSQC spectrum (Acetone-d_6) of lentulin F (**7**)

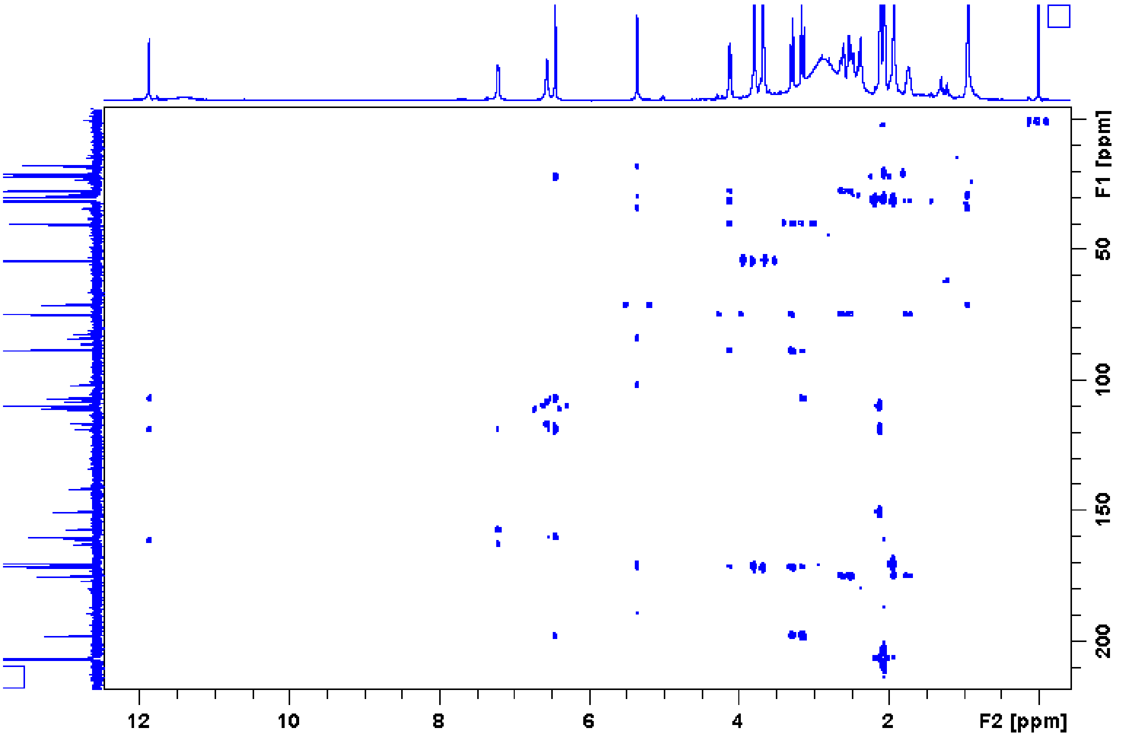

Figure S45. HMBC spectrum (Acetone-d_6) of lentulin F (**7**)

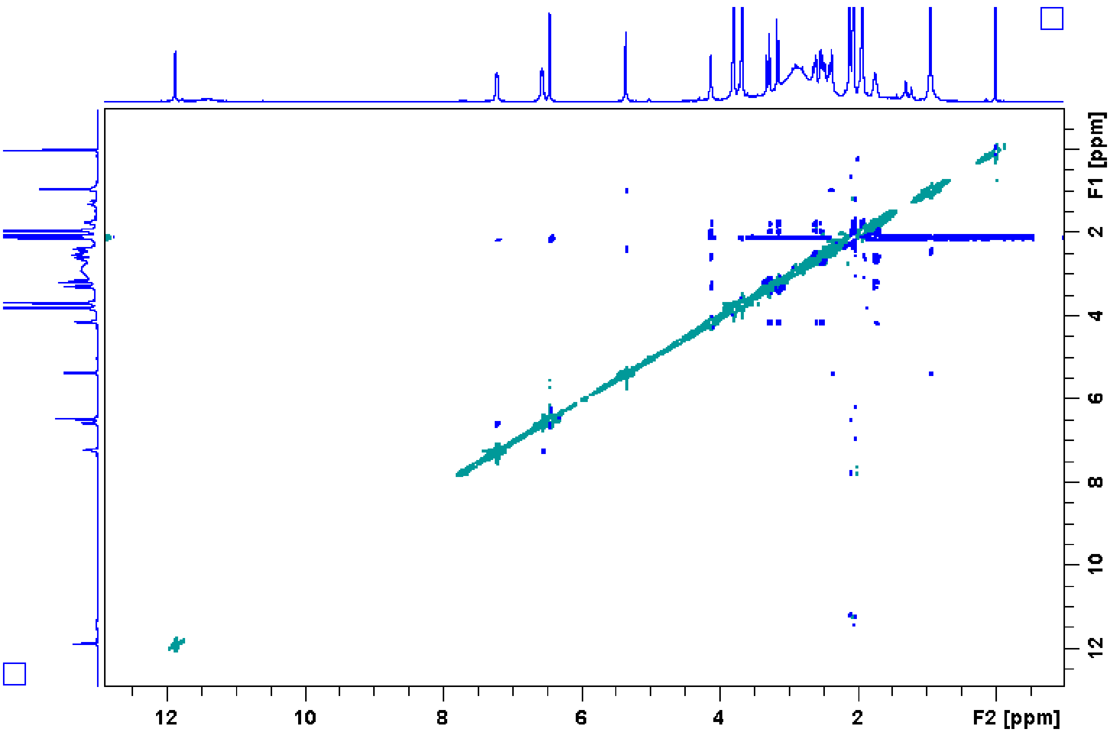

Figure S46. ROESY spectrum (Acetone-d_6) of lentulin F (7)

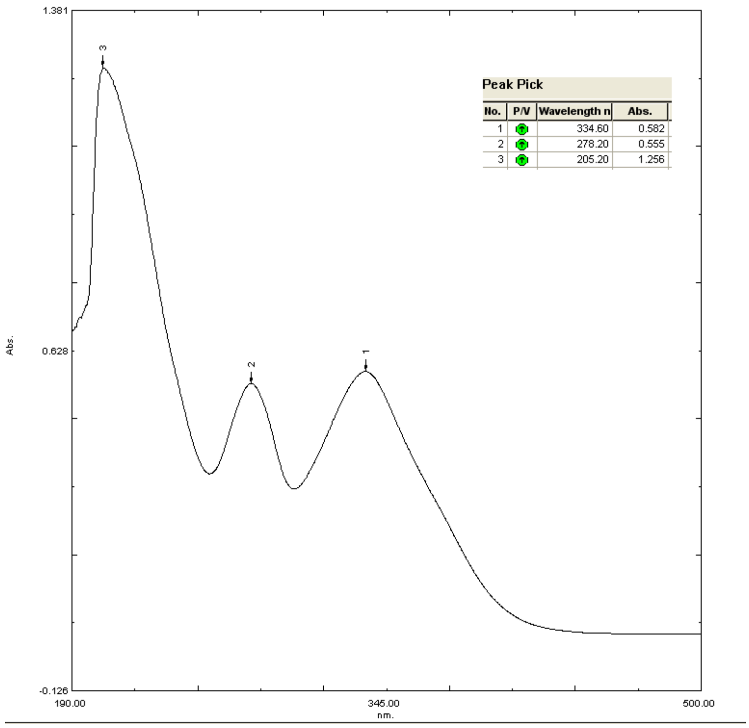

Figure S47. UV spectrum (MeOH) of lentulin F (7)

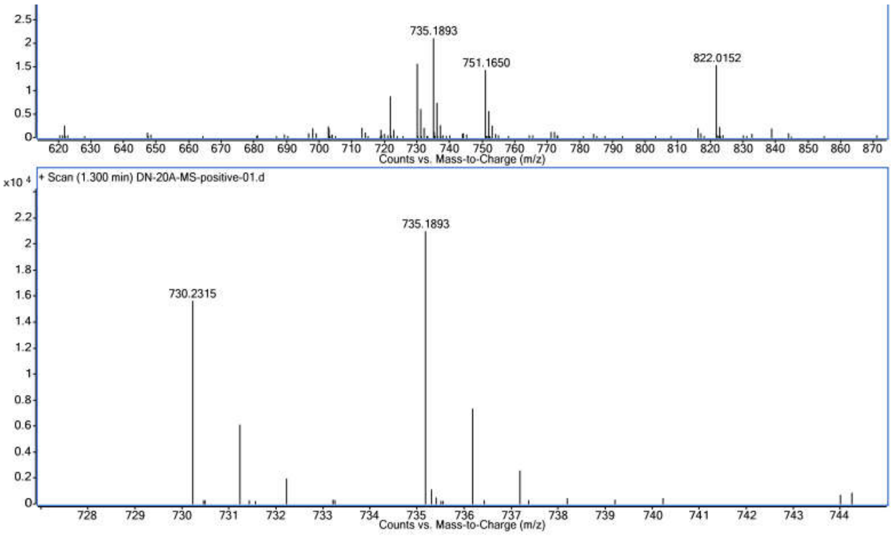

Elemental Composition Calculator

**Target m/z:**	735.1893	**Result type:**	Positive ions	**Species:**	$[M+Na]^+$
**Elements:**		C (0-80); H (0-120); O (0-30); N(0-10); Na (0-5)			
**Ion Formula**		**Calcalated m/z**		**PPM Error**	
C35H36NaO16		735.1896		0.36	

Figure S48. HRESIMS spectrum of lentulin G (**8**)

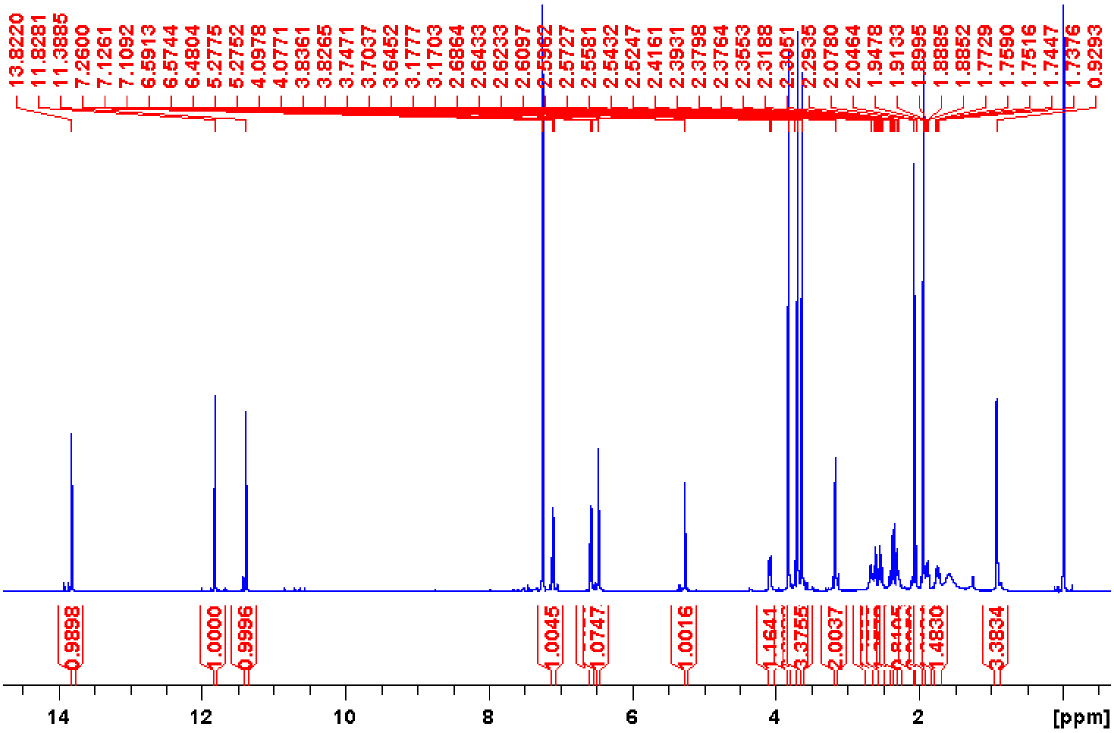

Figure S49. ^{1}H NMR spectrum (500 MHz, $CDCl_3$) of lentulin G (**8**)

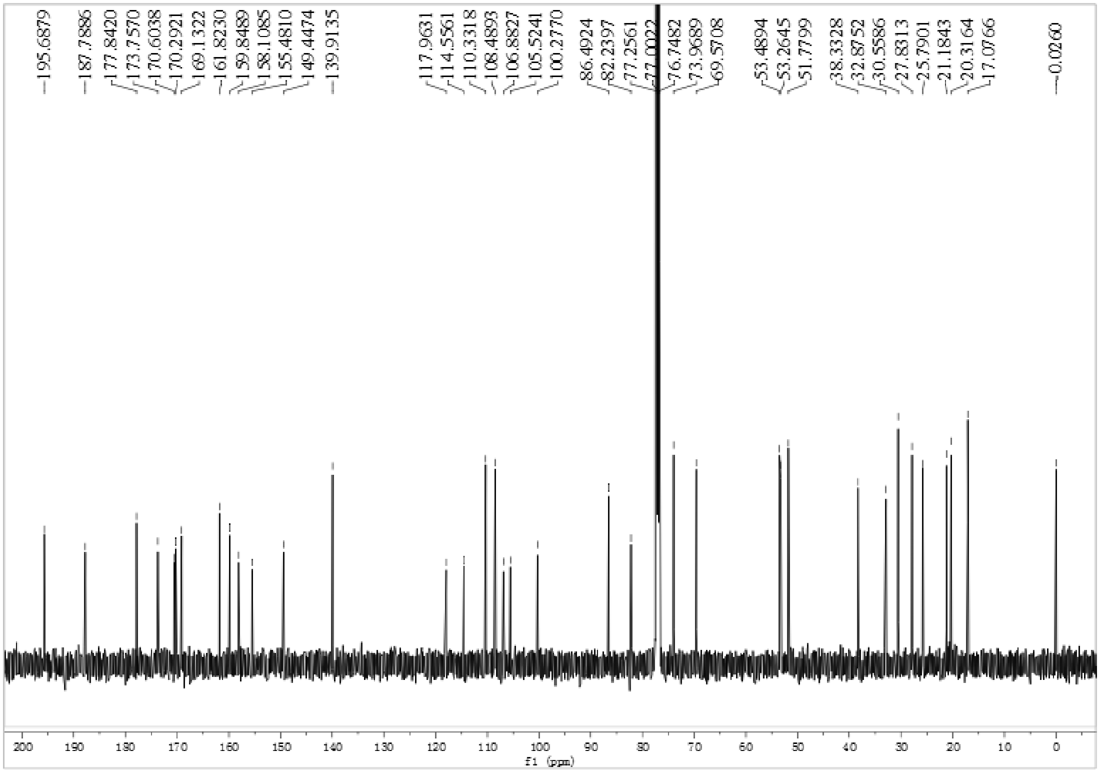

Figure S50. ^{13}C NMR spectrum (125 MHz, $CDCl_3$) of lentulin G (**8**)

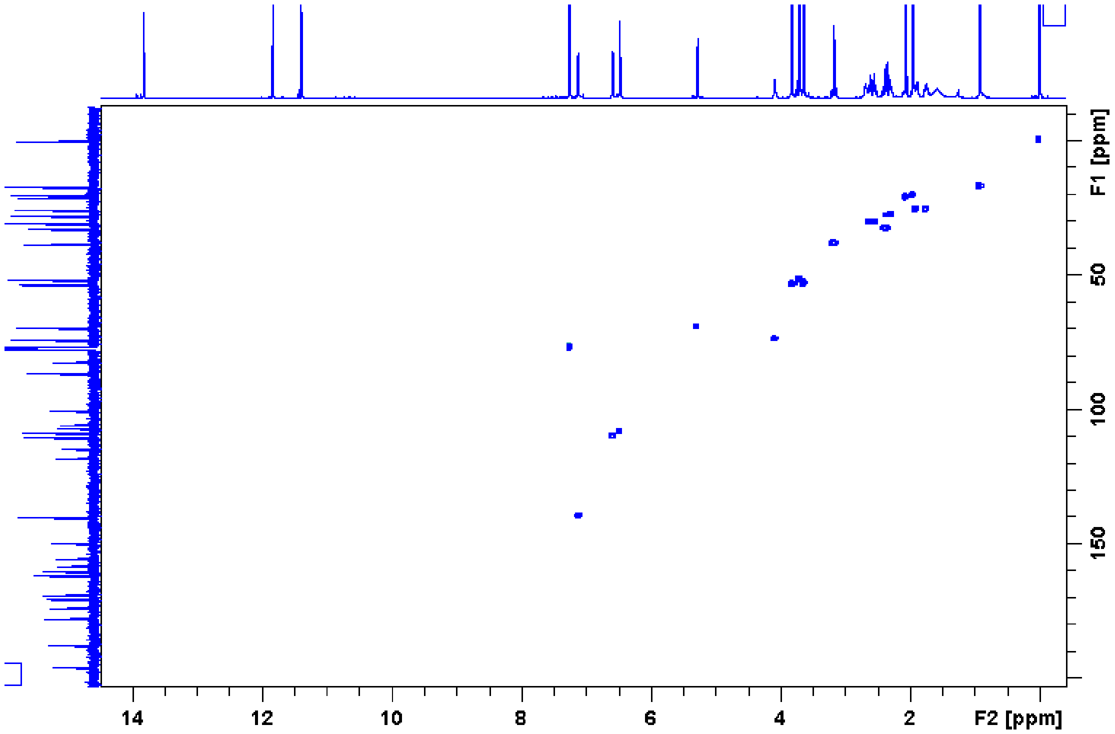

Figure S51. HSQC spectrum ($CDCl_3$) of lentulin G (**8**)

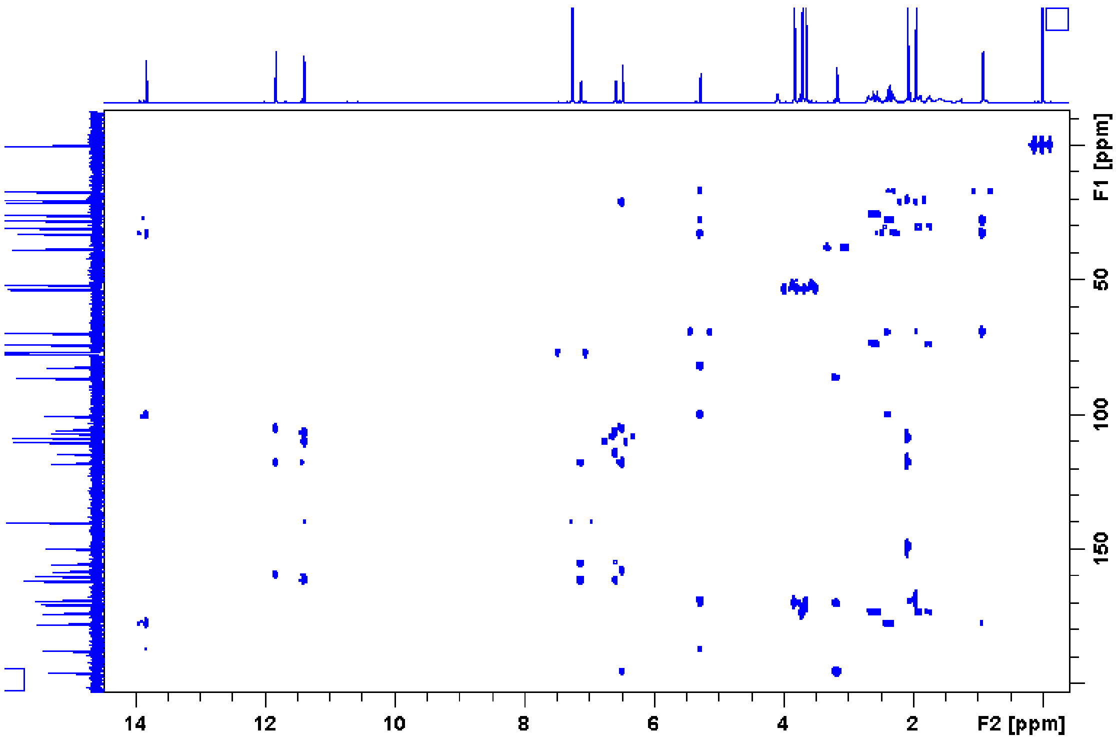

Figure S52. HMBC spectrum ($CDCl_3$) of lentulin G (**8**)

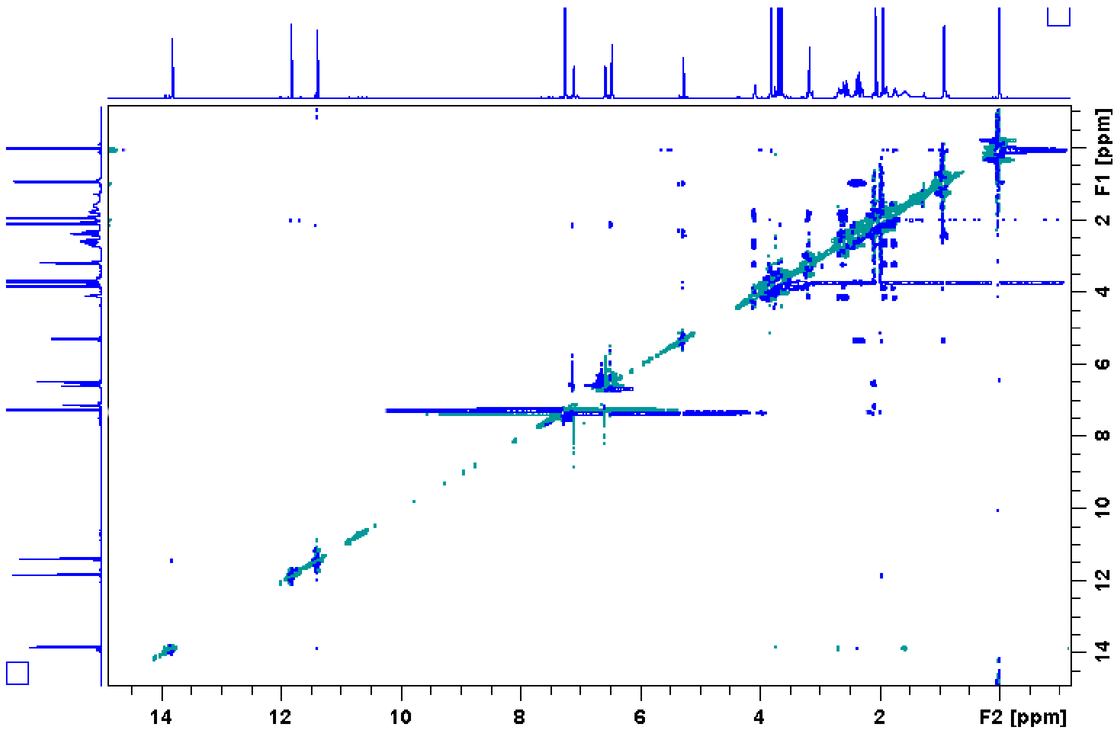

Figure S53. ROESY spectrum ($CDCl_3$) of lentulin G (**8**)

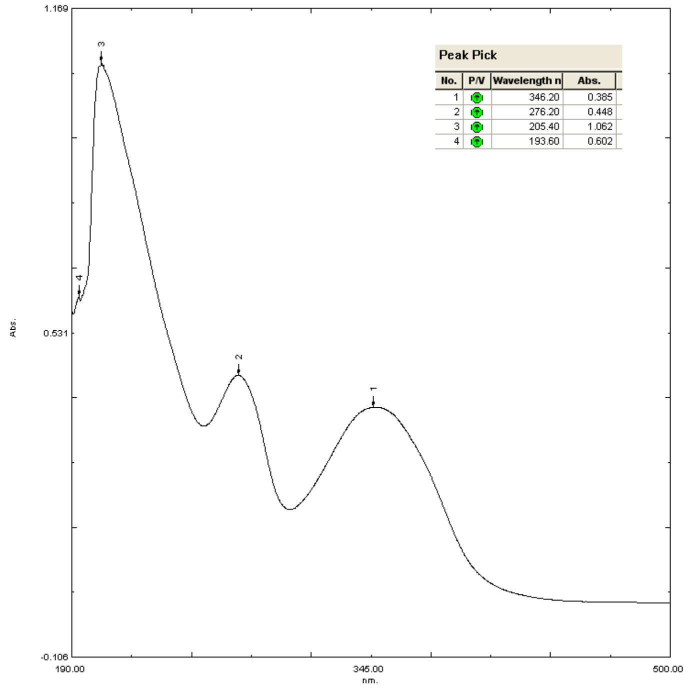

Figure S54. UV spectrum (MeOH) of lentulin G (**8**)

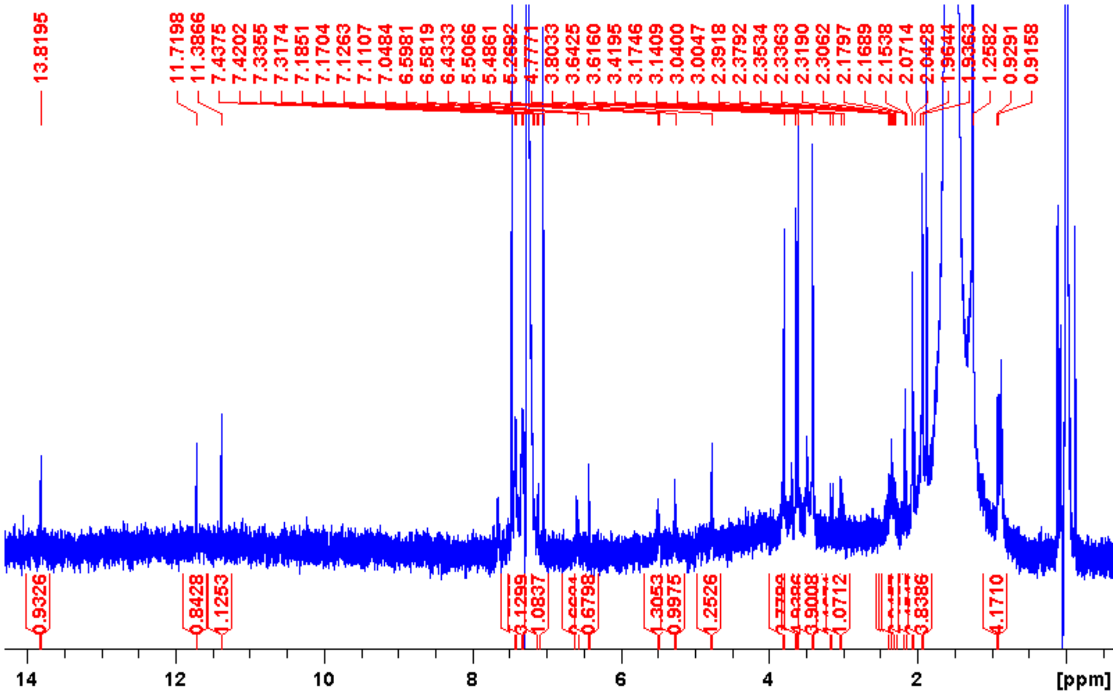

Figure S55. ^{1}H NMR spectrum (500 MHz, $CDCl_3$) of lentulin G

(*R*)-MPA ester **8a**

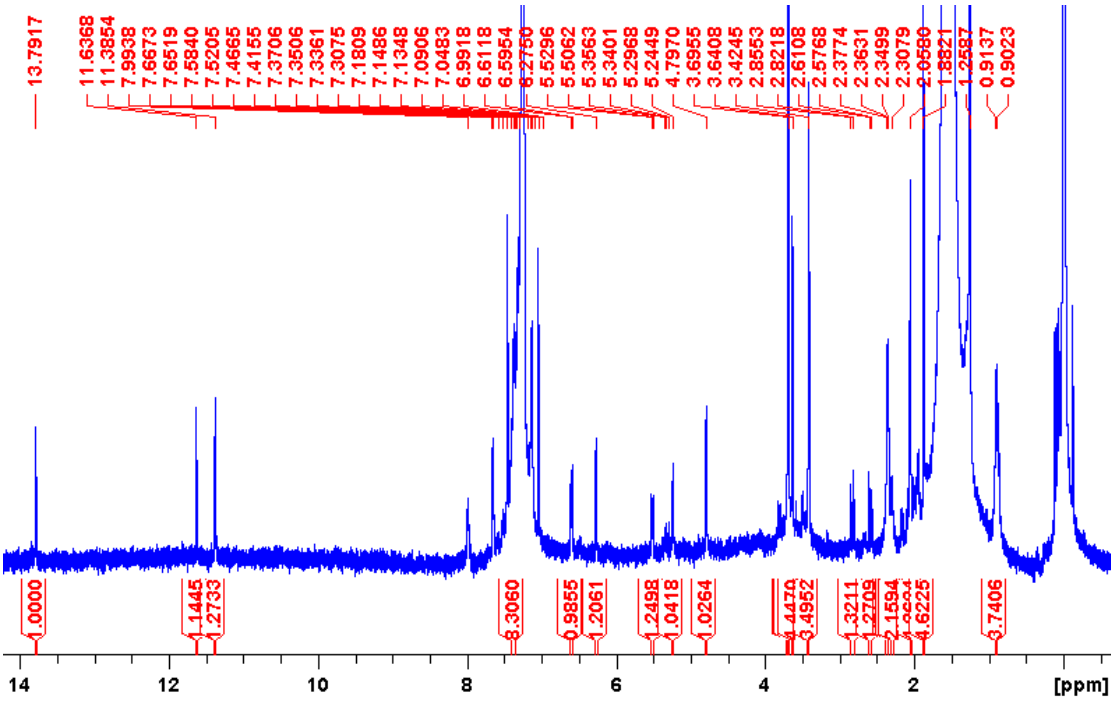

Figure S56. ^{1}H NMR spectrum (500 MHz, $CDCl_3$) of lentulin G (*S*)-MPA ester **8b**

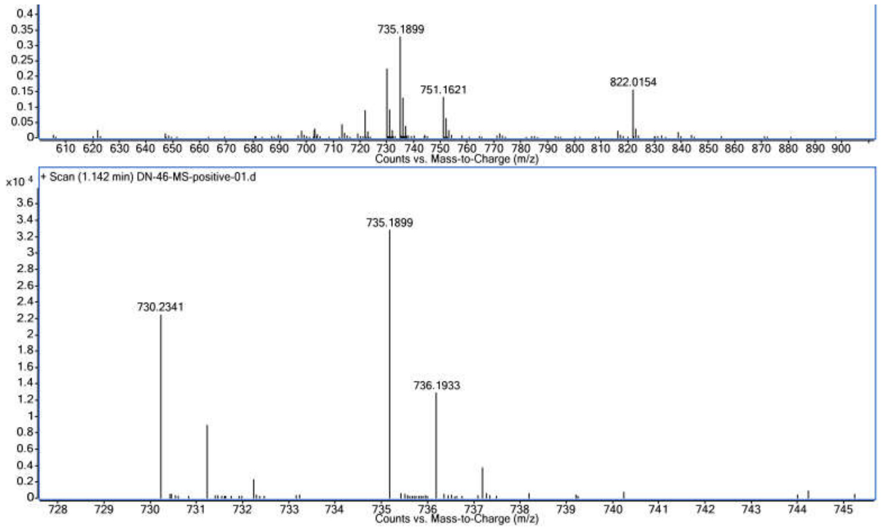

Elemental Composition Calculator

**Target m/z:**	735.1899	**Result type:**	Positive ions	**Species:**	$[M+Na]^+$
**Elements:**		C (0-80); H (0-120); O (0-30); N(0-10); Na (0-5)			
**Ion Formula**		**Calcalated m/z**		**PPM Error**	
C35H36NaO16		735.1896		-0.48	

Figure S57. HRESIMS spectrum of lentulin H (**9**)

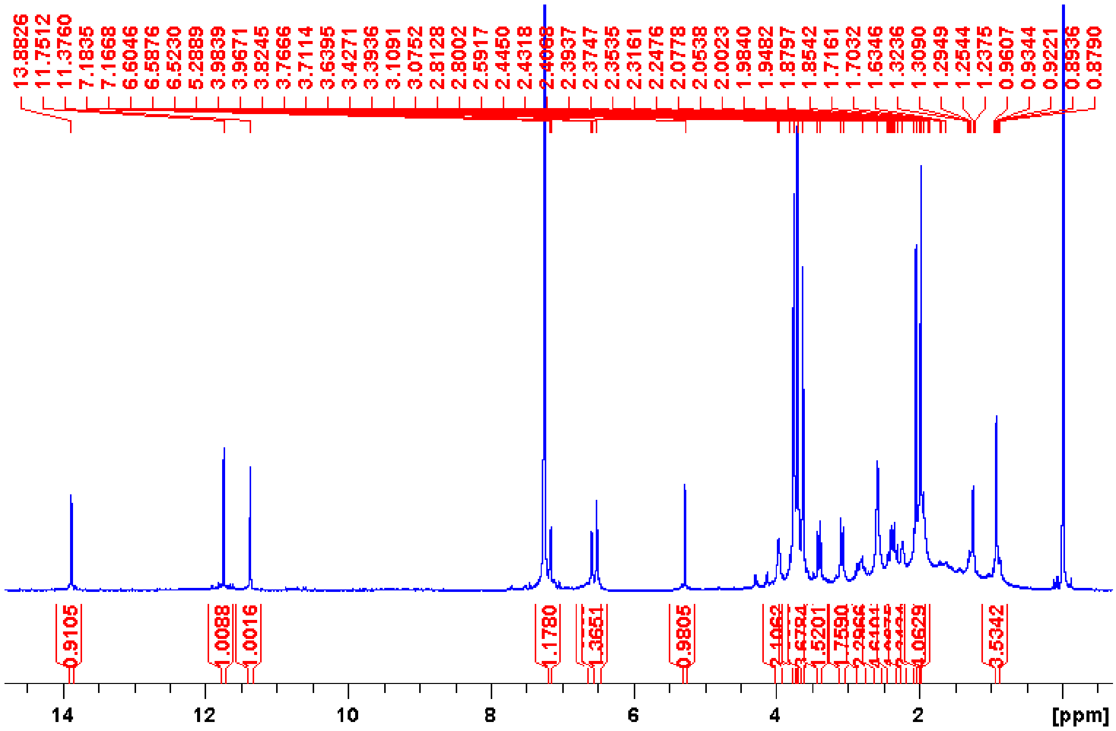

Figure S58. ^{1}H NMR spectrum (500 MHz, $CDCl_3$) of lentulin H (**9**)

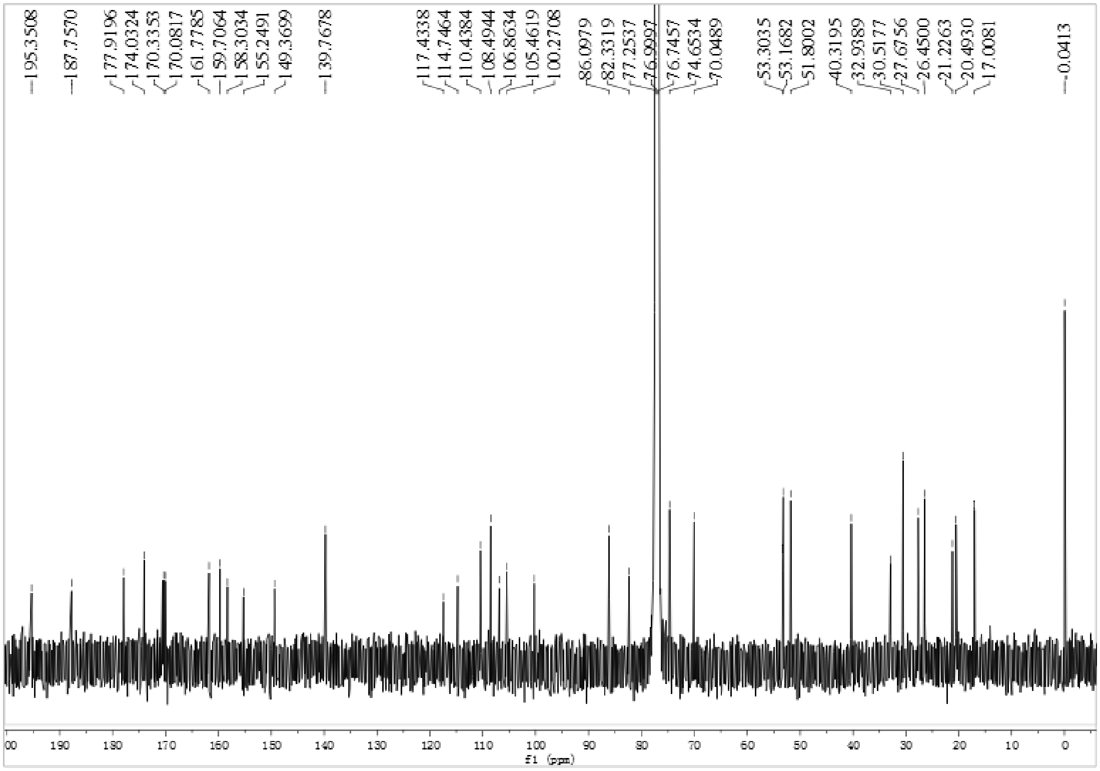

Figure S59. ^{13}C NMR spectrum (125 MHz, $CDCl_3$) of lentulin H (**9**)

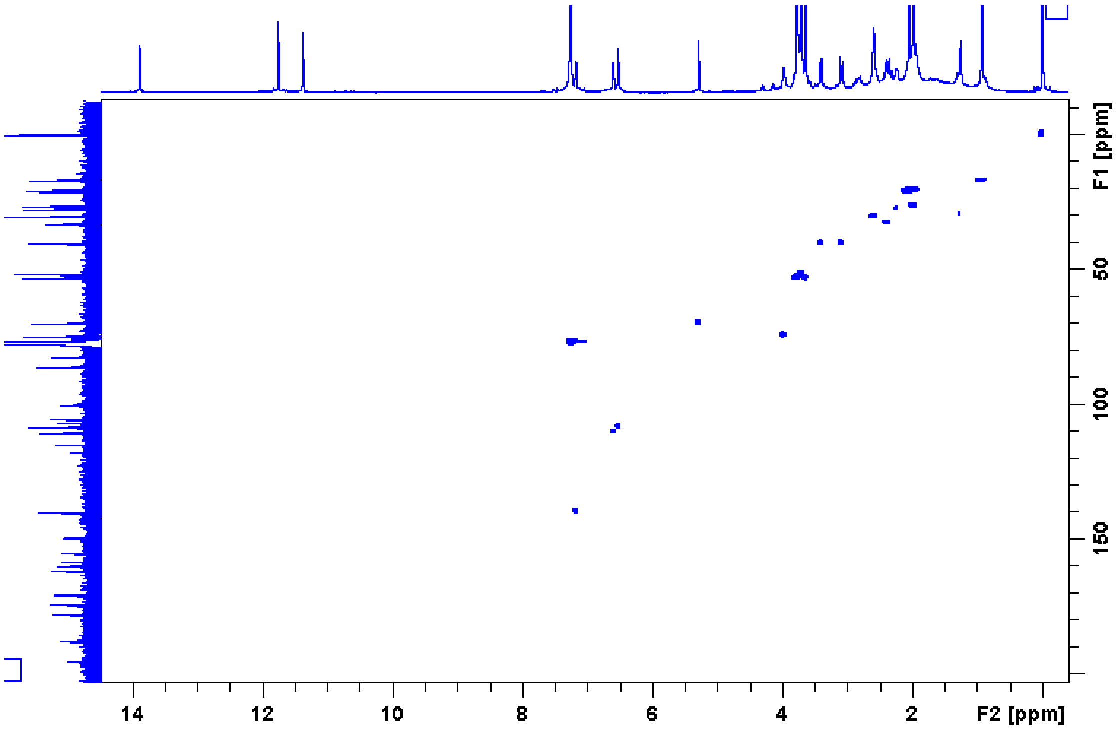

Figure S60. HSQC spectrum ($CDCl_3$) of lentulin H (**9**)

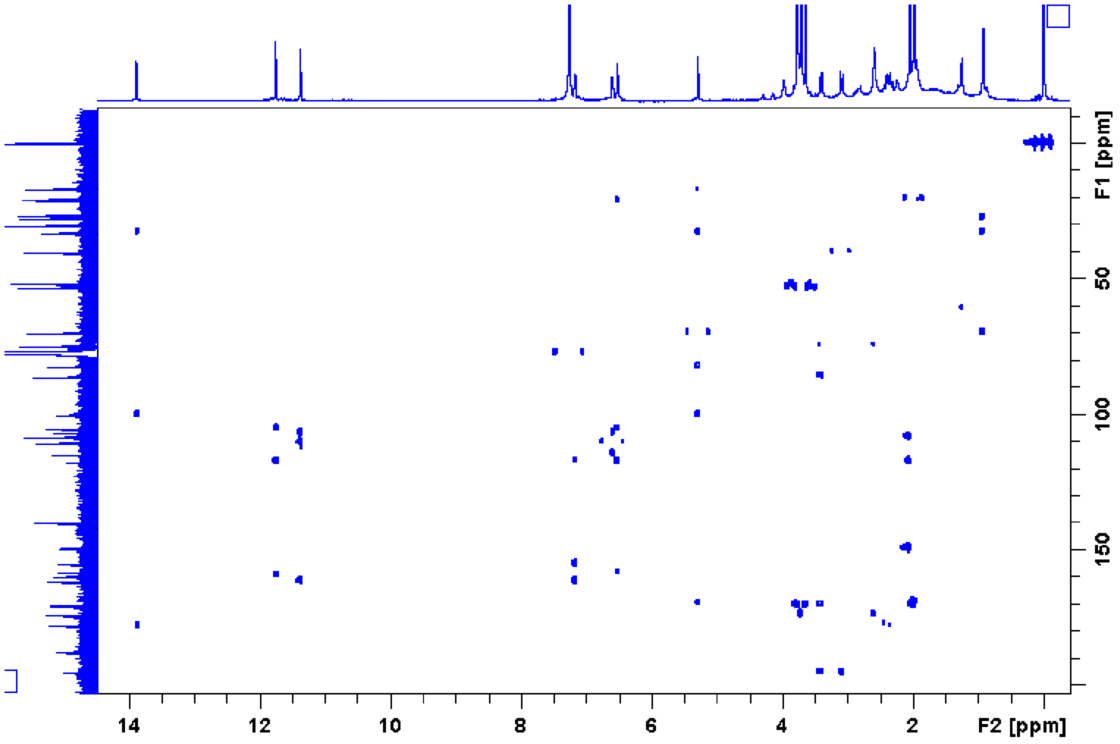

Figure S61. HMBC spectrum ($CDCl_3$) of lentulin H (**9**)

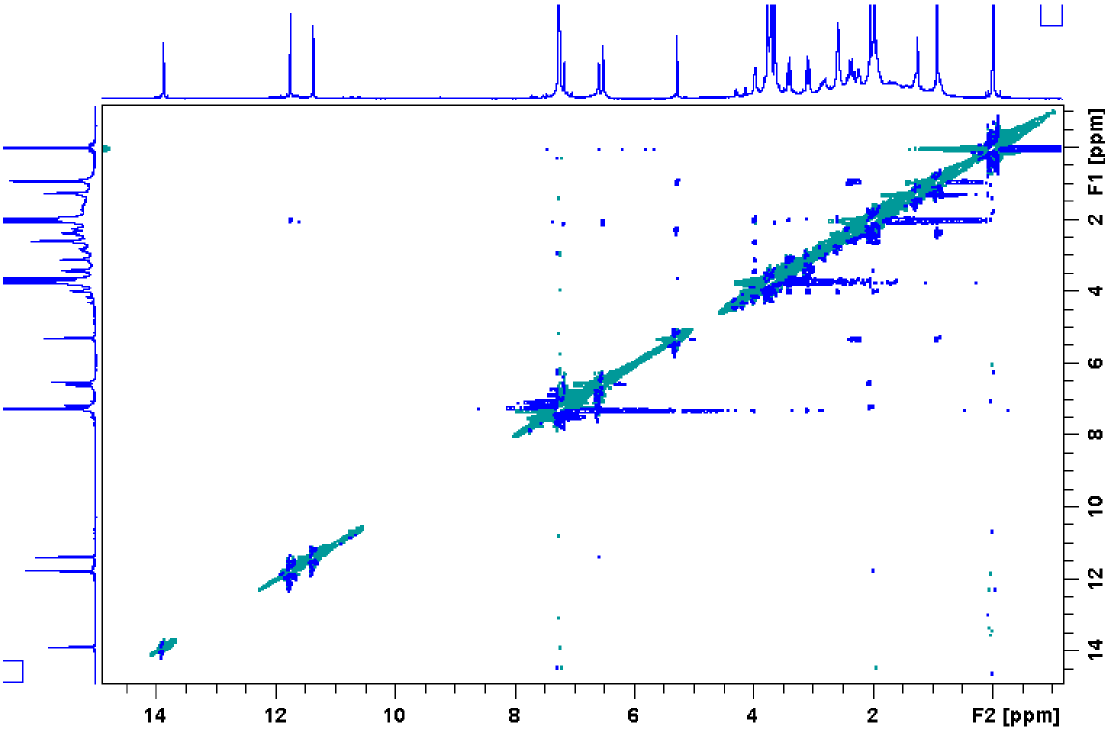

Figure S62. ROESY spectrum ($CDCl_3$) of lentulin H (**9**)

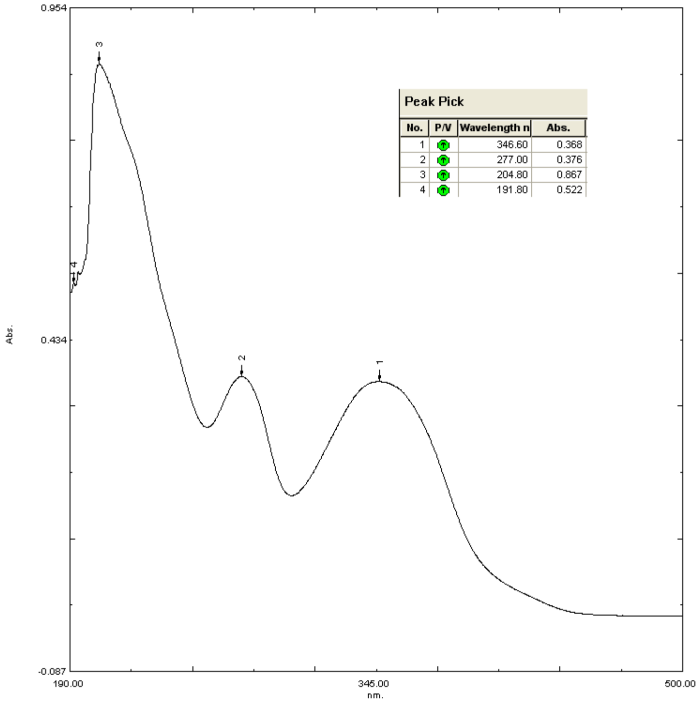

Figure S63. UV spectrum (MeOH) of lentulin H (**9**)

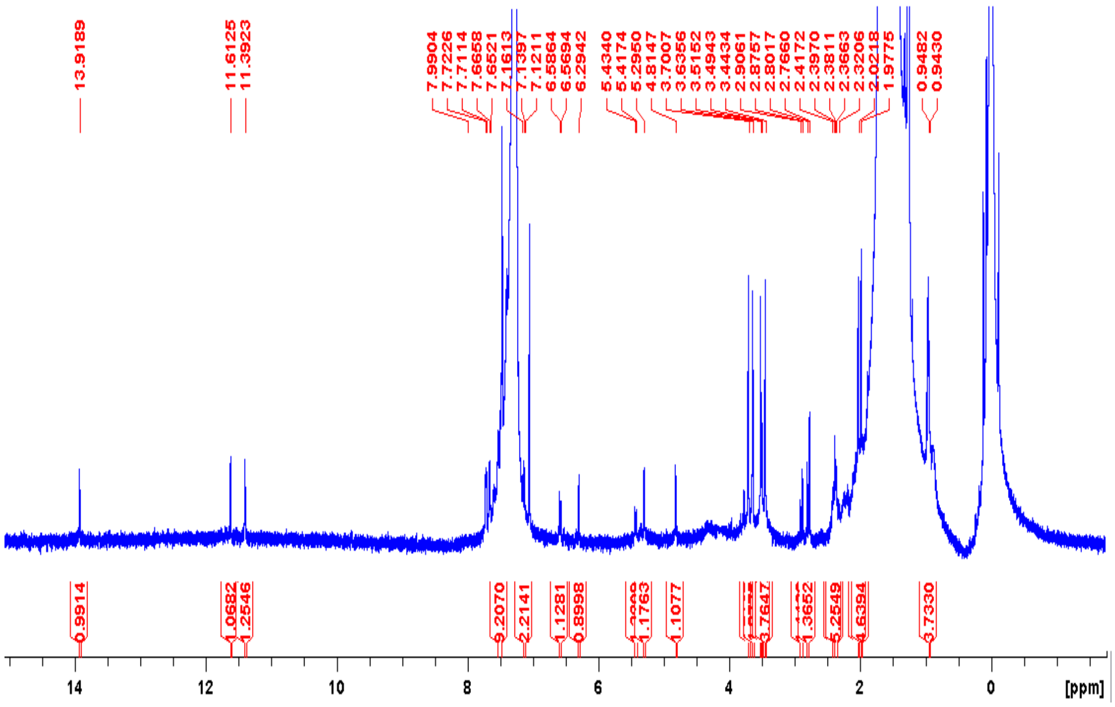

Figure S64. ^{1}H NMR spectrum (500 MHz, $CDCl_3$) of lentulin H (*R*)-MPA ester **9a**

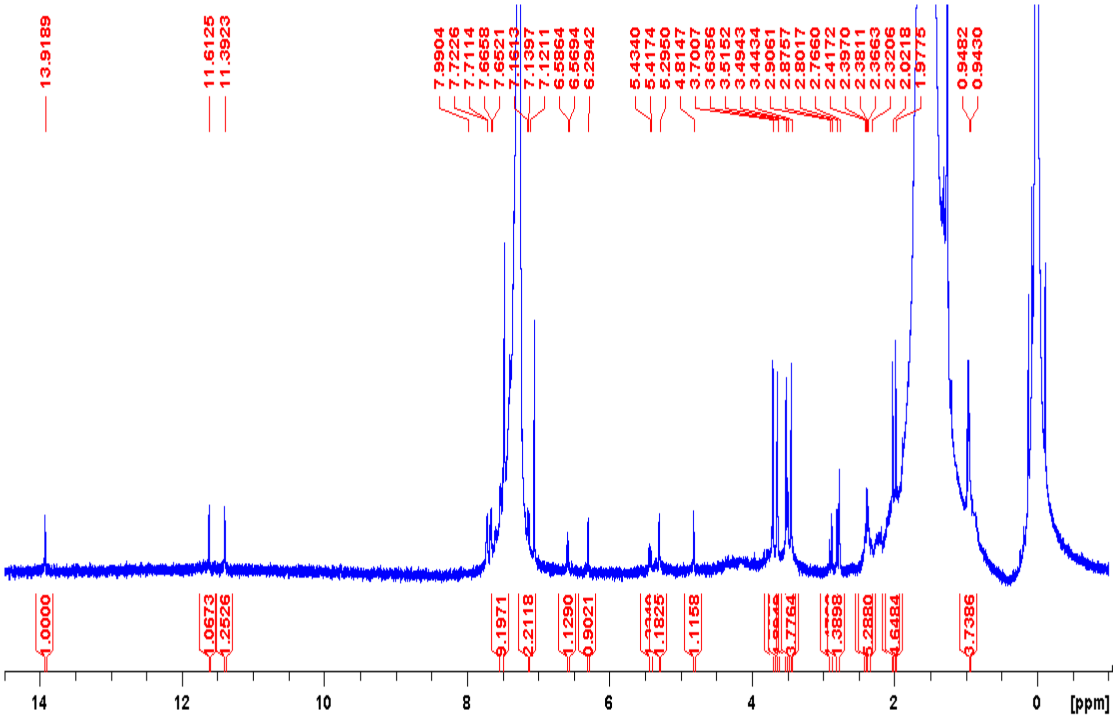

Figure S65. ^{1}H NMR spectrum (500 MHz, $CDCl_3$) of lentulin H (*S*)-MPA ester **9b**
